# Phosphoglycerate dehydrogenase stabilizes *protein kinase C* *delta type* mRNA to promote hepatocellular carcinoma progression

**DOI:** 10.1038/s41392-025-02304-w

**Published:** 2025-07-18

**Authors:** Bin Cheng, Pai Peng, Shi Chen, Rui Liu, Xiaosong Li, Ke Wang, Jing Ma, Kai Wang, Ni Tang, Ailong Huang

**Affiliations:** 1 Department of Infectious Diseases, Key Laboratory of Molecular Biology for Infectious Diseases (Ministry of Education), Institute for Viral Hepatitis, the Second Affiliated Hospital, Chongqing Medical University, Chongqing, China; 2 Clinical Molecular Medicine Testing Center, The First Affiliated Hospital of Chongqing Medical University, Chongqing, China

**Keywords:** Molecular biology, Cell biology

## Abstract

Metabolic reprogramming not only reshapes cellular bioenergetics but also profoundly influences RNA metabolism through metabolite signaling and the RNA-binding activities of metabolic enzymes. Emerging evidence highlights that certain metabolic enzymes act as RNA-binding proteins (RBPs) to regulate gene expression and promote tumor progression. However, the non-catalytic post-transcriptional regulatory functions of metabolic enzymes in hepatocellular carcinoma (HCC) remain largely unexplored. In this study, we performed RNA-protein interactome profiling to identify potential non-canonical RBPs in HCC cells and established phosphoglycerate dehydrogenase (PHGDH) as a functional RBP. We further uncovered a previously unrecognized RNA-binding domain in PHGDH that directly binds cellular mRNAs and plays a key role in HCC cell proliferation. Mechanistically, PHGDH bound directly to the 3’untranslated region (3’UTR) of *protein kinase C delta* *type* (*PRKCD*) mRNA via its RNA-binding domain, thereby stabilizing the transcript and elevating PRKCD protein levels. PHGDH-dependent PRKCD upregulation promoted HCC progression by inducing mitophagy and inhibiting apoptosis. Additionally, decoy oligonucleotides that specifically block the RNA-binding activity of PHGDH markedly impaired its regulation of target genes and suppress HCC cell proliferation. Combination therapy using decoy oligonucleotides or the PRKCD inhibitor sotrastaurin with sorafenib synergistically inhibited HCC progression. Collectively, our findings reveal a non-canonical role of PHGDH in regulating mRNA metabolism and modulating mitophagy. Targeting the RNA-binding activity of PHGDH with decoy oligonucleotides represents a promising therapeutic strategy for HCC.

## Introduction

Hepatocellular carcinoma (HCC) is one of the most common malignancies worldwide and continues to rise in incidence and mortality rates.^1,2^ Early-stage HCC frequently presents asymptomatically, leading to diagnosis at intermediate or advanced stages when curative interventions are limited. Patients with advanced disease face significant challenges, including intrinsic resistance to targeted therapies such as sorafenib and immune checkpoint inhibitors, resulting in an unfavorable prognosis and low survival rates.^3^ Consequently, the identification of novel therapeutic targets and the development of innovative clinical strategies for HCC are urgently needed. Metabolic reprogramming, a fundamental hallmark of cancer cells, not only rewires cellular bioenergetics to supply energy and biomass required for uncontrolled proliferation, but also exerts profound effects on gene expression networks through metabolite-mediated signal transduction and non-canonical functions of metabolic enzymes.^4–7^ Traditionally, metabolic enzymes were thought to solely catalyze biochemical reactions in central metabolic pathways, but emerging evidence over the past decade has uncovered an unexpected role of them. Metabolic enzymes act as RNA-binding proteins (RBPs) that directly regulate RNA metabolism and contribute to the progression of tumors and other diseases.^8,9^ For example, isocitrate dehydrogenase-1 (IDH1) interacts with the GA- or AU-rich motifs of RNAs related to transcriptional regulation, cell cycle and RNA processing to modulate translation.^10^ Enolase-1 facilitates the mRNA degradation of iron regulatory protein 1 as RBP and consequently inhibits mitochondrial iron-induced cell death and promotes tumor progression.^11^ These findings demonstrate that the interplay between metabolic enzymes and RNA metabolism affects gene expression and cellular functions. This paradigm shift has opened new avenues for understanding how cancer cells coordinate metabolic rewiring with transcriptional programs to drive malignant phenotypes, making them potential targets for cancer treatment.

RBPs represent a highly conserved and large family of proteins, which participate in the comprehensive regulation of RNA metabolism, including transcription, translation, splicing, polyadenylation, stability and localization.^12^ In cancer, RBPs are extensively involved in the modulation of gene expression at the transcriptional, post-transcriptional, translational, and post-translational levels, forming intricate and dynamic signaling networks. These regulatory networks profoundly influence multiple cancer hallmarks and the tumor immune microenvironment.^13,14^ Throughout the various stages of RNA processing and maturation, RBPs interact with distinct RNA species to form ribonucleoprotein complexes, thereby regulating RNA splicing, export, localization, translation and stability. RBPs are critical for maintaining cellular homeostasis, and their dysregulation in expression or function severely disrupts cellular physiology and contribute to disease development. Specifically during hepatocarcinogenesis, RBPs mediate transcriptional and post-transcriptional regulation throughout disease progression, from steatosis and fibrosis through cirrhosis to early-stage HCC.^15^ However, the roles of metabolic enzymes that function as RBPs in HCC progression remain largely unknown. Elucidating the RBP functions of metabolic enzymes and their regulatory mechanisms in HCC promises to expand our understanding of tumor metabolism and provide novel therapeutic targets and mechanistic insights for anti-tumor therapy.

It is now well established that various RNA species, including lncRNAs, miRNAs, circRNAs and mRNAs, can interact with RBPs, with mRNAs representing one of the most prominent targets.^16^ Therefore, to identify novel RBPs interacting with mRNA, we applied a UV crosslinking-based RNA interactome capture approach to HCC cells for the first time.^17,18^ We identified phosphoglycerate dehydrogenase (PHGDH) as a potential RBP among the candidate proteins enriched in metabolic pathways. PHGDH, the pivotal enzyme in serine biosynthesis, exhibits high expression in various cancers, thereby facilitating malignant progression of tumors.^19–21^ In addition to its enzymatic function, the non-classical activities of PHGDH contribute to tumor progression.^22–27^ For example, PHGDH facilitates the translation of proteins encoded by mitochondrial DNA through protein-protein interactions in HCC cells.^24^ Decreased expression of the PHGDH protein results in the loss of this interaction with phosphofructokinase, increasing sialylation of integrin αvβ3, thereby enhancing tumor metastasis.^26^ Moreover, increased nuclear localization of PHGDH under glucose deprivation conditions inhibits PARP1 activity, reduces c-Jun transcriptional activity and promotes tumor growth.^25^ Nevertheless, no evidence currently exists for the direct binding of PHGDH to mRNAs or its role in regulating post-transcriptional RNA metabolism, apart from its non-canonical functions via the above-mentioned protein-protein interactions. As a potential target, exploring whether PHGDH promotes HCC progression through non-canonical functions from the perspective of RNA metabolism regulation may provide additional strategies for anti-cancer drugs that target PHGDH.

In this study, we identify PHGDH as an RBP that interacts with mRNA by defining its RNA -binding profile and identifying specific interaction motifs. By mapping the PHGDH-RNA interactome, we report that PHGDH enhances PRKCD protein levels by stabilizing its corresponding mRNA, which in turn facilitates mitophagy to support the malignant progression of HCC. Moreover, decoy oligonucleotides designed and synthesized to block PHGDH’s RNA-binding activity-by mimicking its binding motifs in *PRKCD* mRNA- effectively inhibit HCC progression. Additionally, both decoy oligonucleotides and the PRKCD inhibitor sotrastaurin demonstrate efficacy in vivo and in vitro, exhibiting synergistic effects when combined with sorafenib, the current standard-of-care drug for HCC. Our findings reveal a novel non-canonical function of PHGDH and establish a mechanistic link between cellular metabolism, RNA regulation and mitophagy. This mechanism is strongly associated with the malignant progression of HCC and has potential for developing clinical treatment strategies.

## Results

### RNA-binding activity of PHGDH promotes HCC cell proliferation

**Fig. 1:**
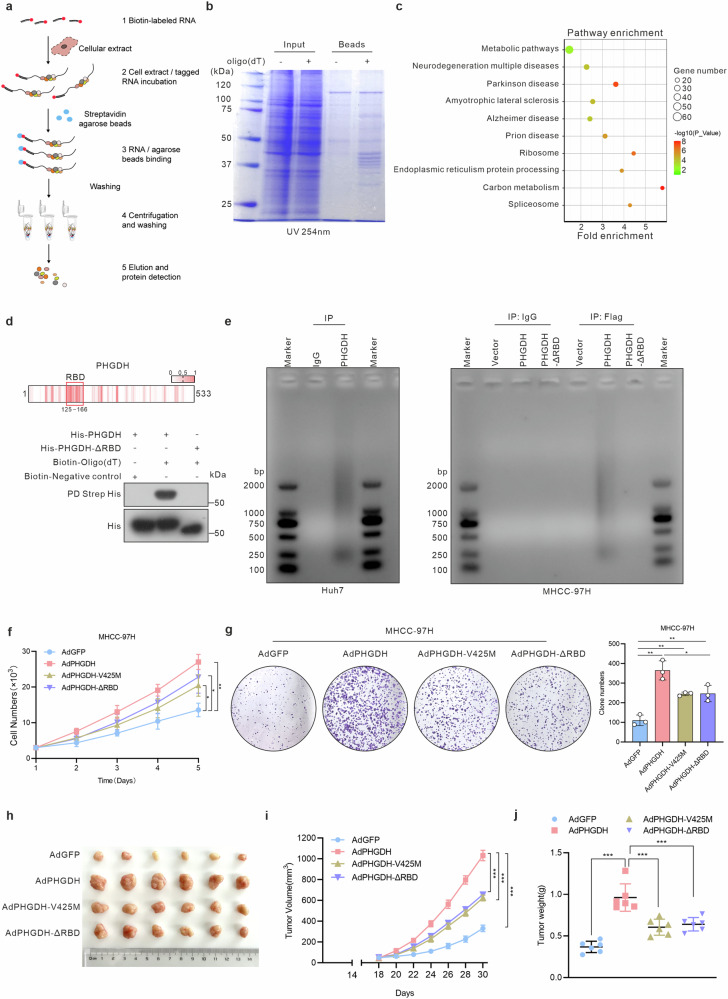
PHGDH binds to mRNAs and promotes the proliferation of HCC cells. **a** Flow chart of RNA pull-down assays. **b** Enriched proteins stained by Coomassie brilliant blue in the RNA pull-down assay. **c** RNA pull-down analysis was conducted on the top 10 significantly downregulated pathway of Kyoto Encyclopedia of Genes and Genomes (KEGG). **d** Prediction of the PHGDH RBD using the RNABindRPlus tool. Western blot analysis of oligo (dT)-bound mRNA pulled down by His-PHGDH or His-PHGDH-ΔRBD. **e** Nucleic acid electrophoresis identification of PHGDH-bound RNA enriched by RNA immunoprecipitation in Huh7 and MHCC-97H cells endogenously expressing PHGDH. **f** Cell proliferation curve of MHCC-97H cells infected with AdPHGDH, AdPHGDH-V425M (a enzymatically inactive PHDGH mutant) or AdPHGDH-ΔRBD (*n* = 3 independent experiments). **g** Colony formation of MHCC-97H cells transfected with PHGDH, PHGDH-V425M or PHGDH-ΔRBD (*n* = 3 independent experiments). **h** Representative images of subcutaneous tumors. MHCC-97H cells were following the indicated treatments and subcutaneously injected into nude mice (*n* = 6 per group). Tumor volume (**i**) and weight (**j**) of implantation tumors. One-way ANOVA followed by Tukey’s test was used in (**f**, **g**, **i**, **j**). Data are represented as the mean ± SD, *P < 0.05, **P < 0.01, ***P < 0.001

To screen for potential RBPs binding to cellular mRNAs in HCC cells, we identified the poly(A) + RNA interactome of Huh7 cells using biotinylated oligo (dT), which can bind to the poly(A) tail of mRNA to pull mRNA-binding proteins out of solution^13,17,18^ (Fig. 1a). Of the approximately 500 mRNA-interacting proteins identified by mass spectrometry, 60 showed significant enrichment in metabolic pathways, as determined by Kyoto Encyclopedia of Genes and Genomes (KEGG) pathway analysis (Fig. 1b, c). A set of metabolic enzymes were included, including IDH1, MDH2, PHGDH and ALDOA (supplementary Table 1). Notably, the RNA-binding function of PHGDH has not previously been reported. Therefore, to further analyze the RNA-binding activity of PHGDH, we used the RNABindRPlus tool to predict RNA-binding domains (RBDs).^13,28^ A predicted RBD was identified between the amino acids 125–166 of PHGDH. To investigate the role of RBD in PHGDH acting as an RBP, we purified the recombinant protein of wild-type (WT) PHGDH and PHGDH mutant lacking the RBD (His-PHGDH-ΔRBD) and performed RNA pull-down assays in vitro. Compared to PHGDH WT, PHGDH-RBD deletion mutant failed to be enriched by oligo (dT) (Fig. 1d). Furthermore, the RNA immunoprecipitation (RIP) results showed that PHGDH-ΔRBD failed to enrich the mRNA like PHGDH WT (Fig. 1e). Collectively, these findings demonstrated, for the first time, the capability of PHGDH to bind to mRNAs.

Previous studies have demonstrated that PHGDH knockdown following extracellular serine supplementation fails to rescue PHGDH-induced cell proliferation,^29–31^ implying a potential role for PHGDH in tumor progression via non-enzymatic mechanisms. Therefore, we investigated the influence of PHGDH RBP function on HCC proliferation. Cellular functional experiments and animal models collectively revealed that the PHGDH enzyme-inactive mutant (V425M)^32^ and the PHGDH-ΔRBD mutant exhibited weaker promotion of HCC cell proliferation compared to the PHGDH WT. However, these mutants also promoted the proliferation of HCC cells (Fig. 1f–j and supplementary Fig. 1a, b). Additionally, PHGDH knockdown significantly inhibited HCC cell proliferation (supplementary Fig. 1c–f). The results above imply that both enzymatic activity and moonlighting function of PHGDH as an RBP can promote HCC progression.

### PHGDH stabilizes *PRKCD* mRNA as an RNA-binding protein

**Fig. 2:**
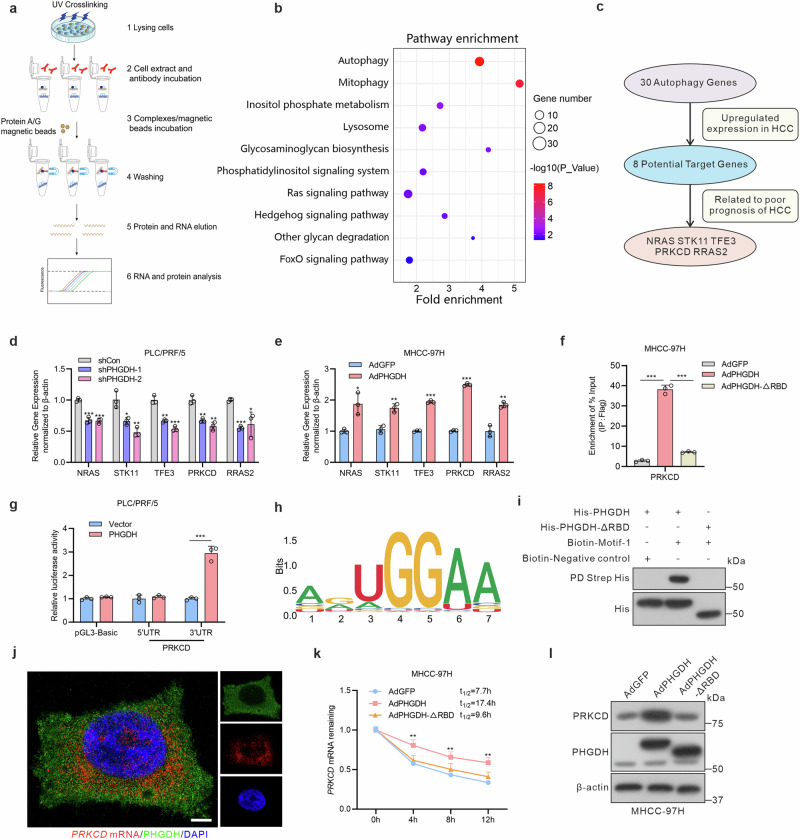
PHGDH enhances *PRKCD* mRNA stability and binds to motif on its 3’UTR. **a** Schematic of RNA immunoprecipitation assays. **b** KEGG pathway analysis of differentially expressed target genes bound to PHGDH by RIP-seq. **c** Workflow analysis of target genes binding to PHGDH that were upregulated in HCC and correlated with unfavorable prognosis. Relative mRNA levels of target genes were assessed in PLC/PRF/5 cells were transfected with shPHGDH or shCon lentivirus (**d**) and MHCC-97H infected with AdPHGDH or AdGFP (**e**) (*n* = 3 independent experiments). **f** RIP-qPCR reveals the interaction between PRKCD transcripts with PHGDH in MHCC-97H cells infected with AdPHGDH or AdPHGDH-ΔRBD (*n* = 3 independent experiments). **g** Relative luciferase activities of *PRKCD* mRNA 5’UTR or 3’UTR with or without PHGDH expression in PLC/PRF/5 cells (*n* = 3 independent experiments). **h** Motif-1 sequence of PRKCD recognized by PHGDH. **i** RNA pull-down assays of purified His-tagged PHGDH protein binding to the biotin-labeled motif. **j** Florescence in situ hybridization experiment of PHGDH (green) and *PRKCD* mRNA (red) in PLC/PRF/5 cells, Scale bar = 5 μm. **k** Half-life of *PRKCD* mRNA was determined in MHCC-97H cells infected with AdPHGDH or AdPHGDH-ΔRBD treated with actinomycin D (the transcription inhibitor, 10 μg/mL) for the indicated durations (*n* = 3 independent experiments). **l** Western blot of PRKCD protein levels in MHCC-97H cells infected with AdPHGDH and AdPHGDH-ΔRBD. P-values were obtained from one-way ANOVA followed by Tukey’s test was used in (**d**, **f**, **k**), whereas an unpaired, two-tailed Student’s *t*-test in (**e, g**). Data are represented as the mean ± SD, *P < 0.05, **P < 0.01, ***P < 0.001

To further understand PHGDH-RNA interactions, we employed RNA immunoprecipitation sequencing (RIP-seq) to identify target genes (Fig. 2a). KEGG pathway analysis of the PHGDH-bound target genes indicated significant enrichment in autophagy and mitophagy pathways (Fig. 2b). Similarly, gene set enrichment analysis revealed notable enrichment in autophagy, selective autophagy and microautophagy when PHGDH was highly expressed (supplementary Fig. 2a–c). Next, by screening candidate genes in the autophagy and mitophagy pathways, we focused on genes that exhibited high expression and closely correlated with poor prognosis in HCC, namely NRAS, STK11, TFE3, PRKCD and RRAS2 (Fig. 2c). Subsequently, we evaluated the modulation of mRNA levels of these candidate genes by PHGDH. The results confirmed that these five genes were significantly upregulated by PHGDH and suppressed by PHGDH knockdown (Fig. 2d, e and supplementary Fig. 2d, e). Furthermore, we observed that both endogenous and exogenous PHGDH significantly enriched *PRKCD* mRNA to the highest extent. In contrast, the other four genes exhibited minimal or negligible enrichment compared to *PRKCD* mRNA (supplementary Fig. 2f, g). In contrast, PHGDH-ΔRBD was ineffective at enriching *PRKCD*, *NRAS* and *STK11* mRNA (Fig. 2f and supplementary Fig. 2h, i).

To identify the specific binding site of PRKCD to PHGDH, we constructed luciferase reporter plasmids containing the 5’ untranslated region (5’UTR) or 3’ untranslated region (3’UTR) of *PRKCD* mRNA. Dual-luciferase assays revealed a significant increase in expression of *PRKCD* mRNA 3’UTR reporter upon PHGDH overexpression in HCC cells (Fig. 2g and supplementary Fig. 2j). Subsequently, we analyzed the potential motif sequences recognized by PHGDH from the RIP-seq data. RIP-seq analysis identified the top 10 motifs based on their frequency in target gene sequences. Alignment with the *PRKCD* mRNA 3’UTR revealed two potential motifs: AGUGGAA (motif-1) and AGAAGGC (motif-2) (Fig. 2h and supplementary Fig. 2k). Biotin-labeled motifs were synthesized to evaluate their binding to purified PHGDH protein using an RNA pull-down assay in vitro. Our results showed that only the motif-1 AGUGGAA binds to PHGDH WT (supplementary Fig. 2l). Meanwhile, we confirmed that the biotin-labeled motif-1 AGUGGAA failed to bind to the His-PHGDH-ΔRBD mutant (Fig. 2i). Additionally, we designed a fluorescent probe to label *PRKCD* mRNA based on the motif-1 AGUGGAA. Fluorescence in situ hybridization assays demonstrated the colocalization of PHGDH and *PRKCD* mRNA in HCC cells (Fig. 2j). These results indicate that PHGDH recognizes *PRKCD* mRNA 3’UTR through a specific motif.

As crucial post-transcriptional regulators, RBPs regulate mRNA stability and influence protein levels. Therefore, we verified the effect of PHGDH on the stability of *PRKCD* mRNA. PHGDH WT enhanced *PRKCD* mRNA stability, whereas the PHGDH-ΔRBD mutant fails to stabilize *PRKCD* mRNA as effectively as the PHGDH WT. In contrast, PHGDH knockdown markedly diminished *PRKCD* mRNA stability (Fig. 2k and supplementary Fig. 2m, n, o). Moreover, upon scrutinizing the protein level of PRKCD, we observed that PHGDH-ΔRBD or PHGDH knockdown was incapable of increasing PRKCD protein expression, unlike PHGDH WT (Fig. 2l and supplementary Fig. 2p, q, r). Overall, our findings suggest that PHGDH, by acting as an RBP, enhances the stability of *PRKCD* mRNA through the RBD, consequently facilitating protein accumulation.

### PHGDH recruits IGF2BP3 to enhance the stability of *PRKCD* mRNA

**Fig. 3:**
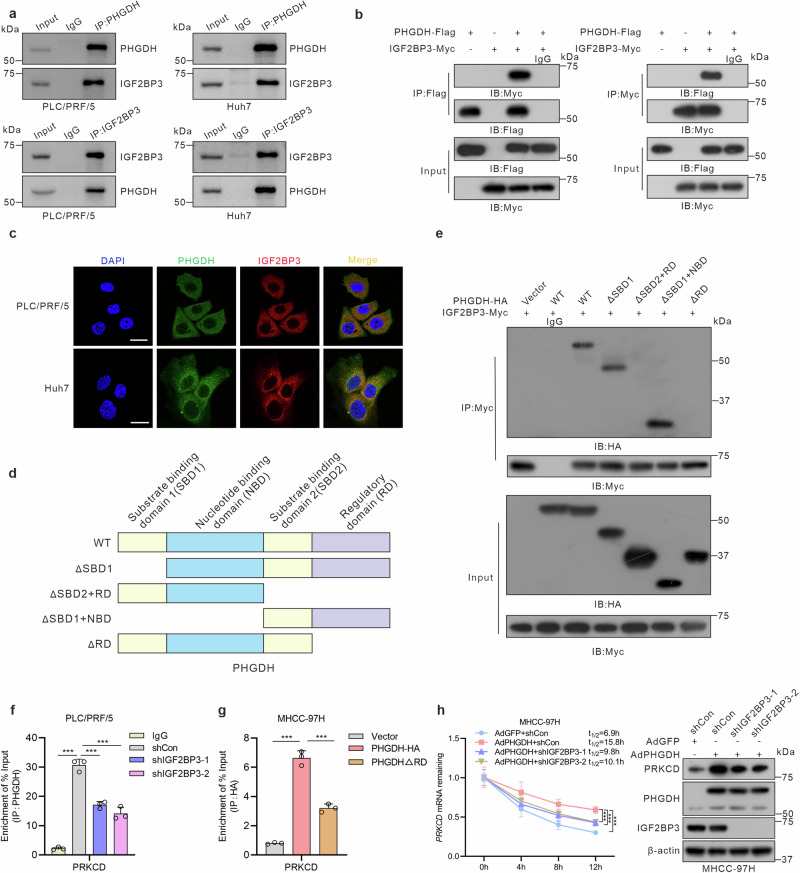
PHGDH interacts with IGF2BP3 through its RD domain and augments the stability *PRKCD* mRNA. **a**, Co-immunoprecipitation (Co-IP) of endogenous PHGDH and IGF2BP3 in PLC/PRF/5 and Huh7 cells. **b** Co-IP of PHGDH-Flag and IGF2BP3-Myc constructs in HEK293 cells. **c** Representative immunofluorescence image for PHGDH and IGF2BP3 in PLC/PRF/5 and Huh7 cells, Scale bar = 25 μm. **d, e** Immunoprecipitation assays of IGF2BP3 and full-length or truncated PHGDH (ΔSBD1, ΔSBD2 + RD, ΔSBD1 + NBD, ΔRD) in HEK293 cells. **f** RIP-qPCR revealed the association of PRKCD transcripts with PHGDH in IGF2BP3-knockdown PLC/PRF/5 cells. **g** RIP-qPCR revealed the association of PRKCD transcripts with PHGDH-HA or PHGDHΔRD in MHCC-97H cells. **h** Half-life of *PRKCD* mRNA in MHCC-97H cells infected with AdPHGDH or AdGFP. Cells were transfected with IGF2BP3 shRNA lentiviral vector (*n* = 3 independent experiments). *P*-values were derived from one-way ANOVA followed by Tukey’s test. Data are represented as the mean ± SD, ***P < 0.001

Given the potential role that RBPs play in RNA regulation by recruiting specific effector molecules,^33–36^ we speculated that PHGDH could recruit certain molecules implicated in the mRNA stability regulation to modulate PRKCD. Initially, through analysis of the BioGRID and HitPredict protein interaction databases, we found that PHGDH may interact with heterogeneous nuclear ribonucleoprotein A1 (HNRNPA1)^37,38^ and insulin-like growth factor 2 mRNA-binding protein 3 (IGF2BP3),^39,40^ which play pivotal roles in regulating mRNA stability. The interaction between PHGDH and IGF2BP3 was confirmed by endogenous Co-immunoprecipitation (Co-IP) in PLC/PRF/5 and Huh7 cells and by exogenous Co-IP in HEK293 and MHCC-97H cells (Fig. 3a, b and supplementary Fig. 3a). However, no interactions were observed between PHGDH and HNRNPA1 (supplementary Fig. 3b). The colocalization of PHGDH and IGF2BP3 was verified using immunofluorescence assays in HCC cells (Fig. 3c). Furthermore, we demonstrated that the PHGDH reductase domain (RD) interacted with the RNA recognition motif domains 1-2 (RRM1-2) of IGF2BP3 (Fig. 3d, e and supplementary Fig. 3c, d).

Subsequently, we explored whether IGF2BP3 influences the modulation of PRKCD mediated by PHGDH. RIP assays indicated that IGF2BP3 knockdown or deletion of the PHGDH RD domain significantly reduced PRKCD enrichment by PHGDH (Fig. 3f, g). To confirm that IGF2BP3 is a critical molecule for regulating mRNA stability, we further examined whether PHGDH requires IGF2BP3 to enhance *PRKCD* mRNA stability. Our results showed that IGF2BP3 knockdown reduced the PHGDH-induced enhancement of *PRKCD* mRNA stability (Fig. 3h). We also observed that IGF2BP3 colocalizes with *PRKCD* mRNA in HCC cells (supplementary Fig. 3e). Moreover, knocking out PHGDH significantly inhibits IGF2BP3-mediated enrichment of *PRKCD* mRNA and reduced the IFG2BP3-induced enhancement of *PRKCD* mRNA stability (supplementary Fig. 3f, g). Collectively, these results suggest that PHGDH recruits IGF2BP3 to stabilize *PRKCD* mRNA.

### PHGDH promotes mitophagy via PRKCD

**Fig. 4:**
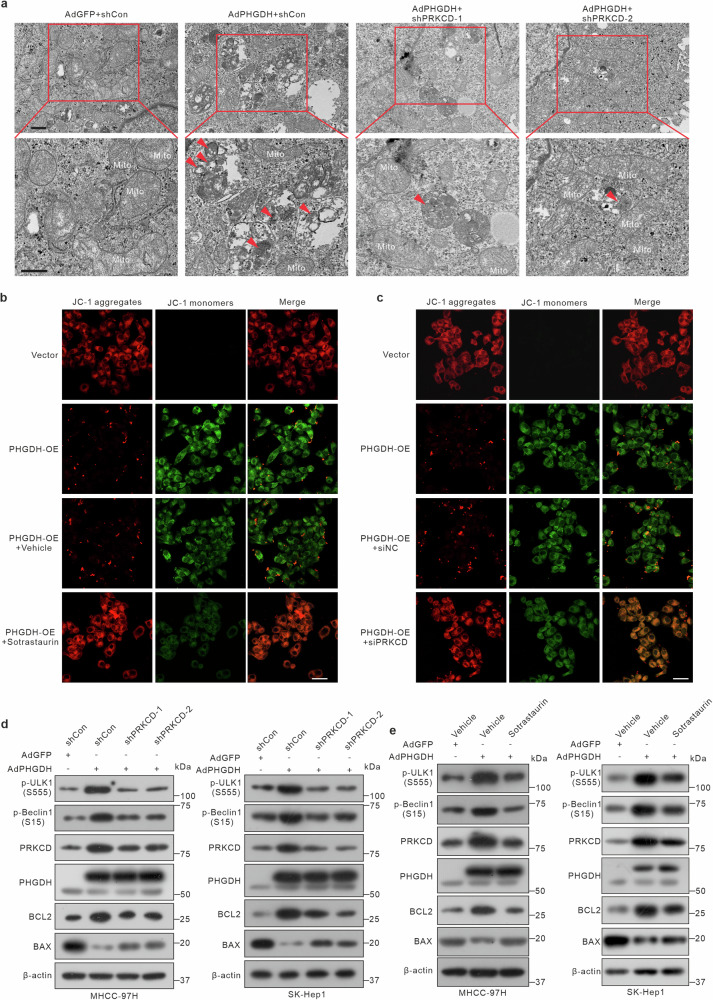
PHGDH activates mitophagy through PRKCD and inhibits apoptosis. **a** Representative transmission electron microscopy snapshots depicting mitophagy in MHCC-97H cells. Cells were infected with AdPHGDH and subsequently transfected with PRKCD shRNA lentiviral vector. The arrows were labeled autophagosomes. Scale bar = 500 nm. **b** Determination of mitochondrial membrane potential in PHGDH-overexpressing MHCC-97H cells supplemented with or without sotrastaurin (2 μM, 24 h). Scale bar = 50 μm. **c** Mitochondrial membrane potential staining assay of PHGDH-expressing MHCC-97H cells transfected with PRKCD siRNA. Scale bar = 50 μm. **d** Western blot of mitophagy and apoptosis makers in PHGDH-expressing MHCC-97H cells transfected with PRKCD shRNA lentiviral vector. **e** Western blot of mitophagy and apoptosis makers in PHGDH-expressing MHCC-97H cells supplemented with or without sotrastaurin (2 μM, 24 h)

PRKCD is regarded as a facilitator of PRKN-independent mitophagy that positively regulates autophagy by recruiting the ULK complex to early autophagic structures.^41^ Therefore, we investigated whether PHGDH affects mitophagy via PRKCD. Transmission electron microscopy revealed that PHGDH increased the accumulation of mitophagosomes, whereas knockdown of PRKCD reduced the number of mitophagosomes (Fig. 4a). Loss of mitochondrial membrane potential is pivotal for initiating mitophagy, which helps clear damaged mitochondria and maintain cellular balance and function.^42–44^ We used the JC-1 fluorescence probe to show that PHGDH resulted in depleted mitochondrial membrane potential. However, treatment with sotrastaurin (a PRKCD inhibitor)^41^ or siRNA in MHCC-97H cells partially restored the membrane potential (Fig. 4b, c). LC3 plays a vital role in forming the autophagosomal membrane. When LC3 aggregates in the mitochondria, it indicates the recruitment of autophagosomes to the mitochondria, which facilitates engulfment. We found that PHGDH significantly increased the aggregation of LC3 in mitochondria, whereas intervention with PRKCD suppressed this effect (supplementary Fig. 4a, b).

The ULK1 complex plays an essential role in the mitophagy initiation process.^45,46^ ULK1 activated by phosphorylation at Ser555 and Beclin1 (the ULK1 substrate) phosphorylation at Ser15 induce mitophagy.^47–49^ Considering the crucial role of PRKCD in recruiting the ULK1 complex to initiate mitophagy, we investigated whether PHGDH activates the ULK1 complex via PRKCD. PHGDH increased the Ser555 phosphorylation of ULK1 and the Ser15 phosphorylation of Beclin1, whereas intervention with PRKCD suppressed this activation (Fig. 4d, e). Mitophagy activation results in the clearance of damaged mitochondria, reducing oxidative stress and suppressing apoptosis and ultimately contributing to cell survival.^50–52^ Concordantly, we noted that PHGDH enhanced the protein level of the anti-apoptotic factor BCL2 while significantly inhibiting the expression of the pro-apoptotic protein BAX (Fig. 4d, e). Meanwhile, Flow cytometry results showed that PHGDH overexpression significantly inhibited apoptosis in HCC cells, and this effect could be reversed by PRKCD intervention (supplementary Fig. 4c). Subsequently, we investigated the effect of PRKCD on PHGDH proliferation in HCC cells. PRKCD knockdown markedly suppressed the PHGDH-induced proliferation of HCC cells (supplementary Fig. 4d–g). Overall, PHGDH activates mitophagy in a PRKCD-dependent manner, while simultaneously suppressing apoptosis.

### Specific binding of decoy oligonucleotides to PHGDH inhibits HCC progression

**Fig. 5:**
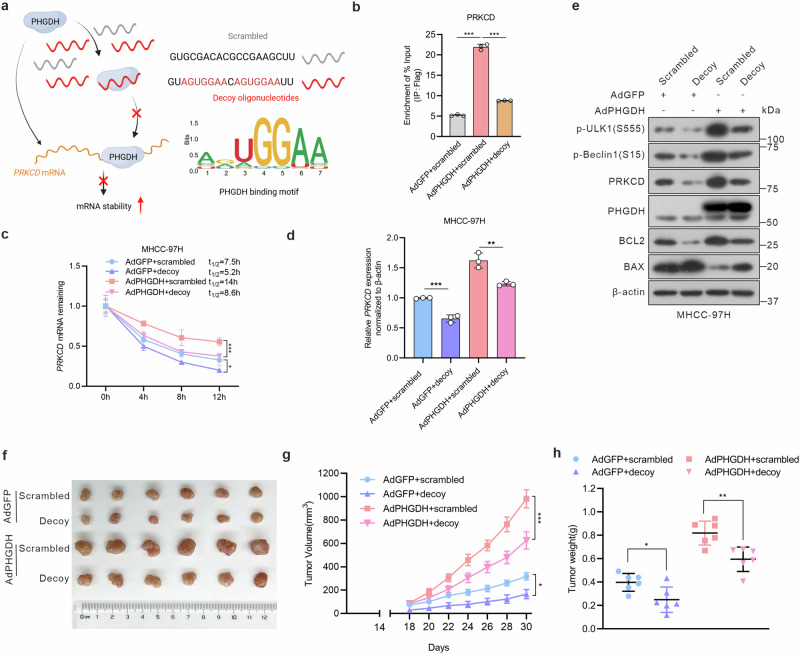
Decoy oligonucleotides inhibit the RNA-binding capacity of PHGDH and its pro-hepatocarcinogenic effect. **a** Schematic representing the suppression of PHGDH RNA-binding activity by decoy oligonucleotides. **b** RIP-qPCR revealed the association of PRKCD transcripts with PHGDH in MHCC-97H cells infected with AdPHGDH or AdGFP and transfected with PHGDH decoy oligonucleotides (*n* = 3 independent experiments). **c**, Half-life of *PRKCD* mRNA in PHGDH-expressing MHCC-97H cells transfected with PHGDH decoy or scrambled oligonucleotides. **d** Relative mRNA levels of PRKCD were determined in MHCC-97H cells following the specified treatments (*n* = 3 independent experiments). **e** Western blot of mitophagy and apoptosis makers in PHGDH-expressing MHCC-97H cells following the specified treatments. **f** Representative images of subcutaneous tumors, MHCC-97H cells were following the indicated treatments and subcutaneously injected into nude mice. Tumor volume (**g**) and weight (**h**) of implantation tumors (*n* = 6 per group). *P*-values were derived from one-way ANOVA followed by Tukey’s test. Data are represented as the mean ± SD, *P < 0.05, **P < 0.01, ***P < 0.001

Decoy oligonucleotides can block the RNA-binding activity of RBPs and inhibit their biological activity both in vitro and in vivo.^53,54^ Therefore, we synthesized RNA decoy oligonucleotides (AGUGGAA) based on the binding motifs of PHGDH target genes (Fig. 5a). RIP-qPCR analysis demonstrated that the decoy oligonucleotides significantly inhibited the enrichment of *PRKCD* mRNA by PHGDH (Fig. 5b). Decoy oligonucleotide treatment therefore decreased PHGDH-induced mRNA stability and protein accumulation in PRKCD and suppressed the protein levels of mitophagy markers while elevating apoptosis (Fig. 5c–e and supplementary Fig. 5a–c). Furthermore, we observed that the decoy oligonucleotides remarkably suppressed the proliferative effect of PHGDH on HCC cells (supplementary Fig. 5d–g). Consistent with in vitro experiments, decoy oligonucleotides significantly suppressed the tumor-promoting effect of PHGDH in a subcutaneous tumor model (Fig. 5f–h). Our results demonstrate that decoy oligonucleotides targeting the RBP function of PHGDH can effectively inhibit the pro-proliferative impact of PHGDH on HCC.

### PHGDH promotes HCC progression by upregulating PRKCD

**Fig. 6:**
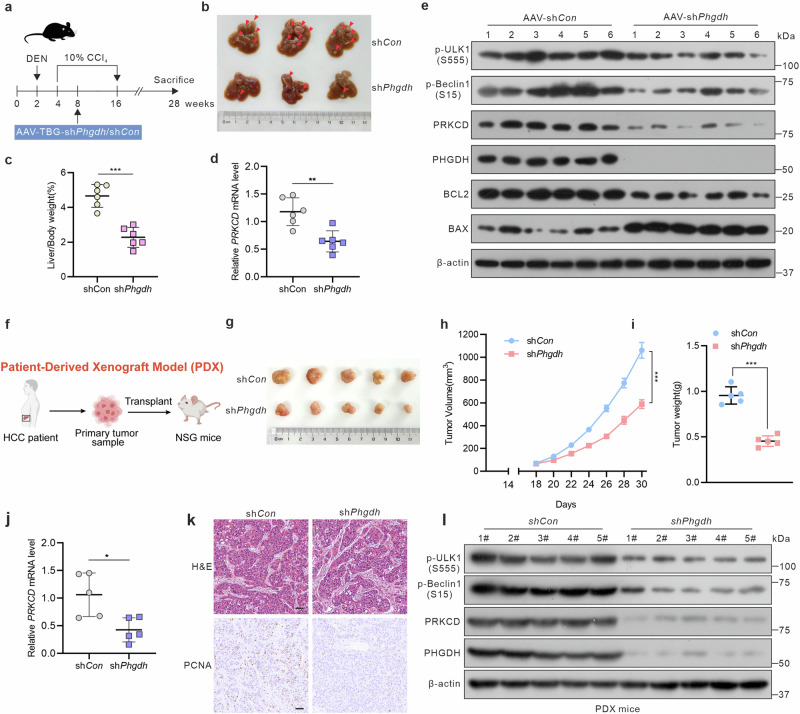
PHGDH promotes HCC proliferation through PRKCD. **a** Diagrammatic representation of a DEN/CCl_4_-induced mouse model of HCC. **b** Representative appearance of liver samples with tumors (*n* = 6 per group). **c** Liver/body weight of DEN/CCl_4_-induced mice. **d** Relative mRNA levels of PRKCD were determined in mice liver samples. **e** Western blot of mitophagy and apoptosis makers in mice liver samples. **f** Diagrammatic representation of Patient-Derived Xenograft (PDX) mice model. **g** Representative appearance of liver tumors in PDX HCC mice model. Tumor volume **(h)** and weight **(i)** of implantation tumors (*n* = 5 per group). **j** Relative mRNA levels of PRKCD were determined in PDX mice liver samples. **k** Representative IHC images of H&E and PCNA in PDX mice, scale bar = 50 μm. **l** Western blot of mitophagy makers in PDX mice liver samples. *P*-values were derived from unpaired two-tailed Student’s *t*-test in (**c**, **d**, **i**, **j**) and unpaired two-tailed multiple Student’s t-test in (**h**). Data are represented as the mean ± SD, *P < 0.05, **P < 0.01, ***P < 0.001

Considering the contribution of PHGDH to promoting HCC proliferation via the PRKCD-mitophagy pathway in cell models, we further investigated whether inhibiting the PHGDH-PRKCD axis would impede HCC progression. Specifically, we achieved liver-specific knockout of PHGDH in diethylnitrosamine and carbon tetrachloride (DEN/CCl_4_)-induced mouse HCC models by intravenous injection of TBG-AAV-sh*Phgdh* or the TBG-AAV-control vector into the tail vein (Fig. 6a). AAV-mediated knockout of PHGDH decreased the number of tumors and liver/body weight ratio in mice (Fig. 6b, c). Consistent with in vitro results, *PRKCD* mRNA levels were significantly reduced in mice following PHGDH knockout (Fig. 6d). Immunoblotting and immunohistochemistry assays revealed a notable reduction in the protein levels of PRKCD and mitophagy molecules but enhanced apoptosis in the mice (Fig. 6e and supplementary Fig. 6a). To further validate the role of the PHGDH-PRKCD axis in HCC progression and the importance of PDX models in this context, we established PDX models and achieved PHGDH knockdown via intratumoral injections of AAV-sh*Phgdh*. The results demonstrated that PHGDH knockdown significantly inhibited PDX tumor growth, reduced *PRKCD* mRNA and protein levels and decreased the expression of mitophagy-related molecules (Fig. 6f–l). These findings in the PDX models are consistent with the results from the DEN/CCl_4_-induced HCC models. Moreover, the tumor-promoting effect of PHGDH was markedly reversed by shRNA- or sotrastaurin-mediated intervention targeting PRKCD (supplementary Fig. 6b–d). These results indicate that PHGDH promotes HCC progression in a PRKCD-dependent manner.

### Targeting the RBP function of PHGDH as a novel strategy for HCC combination therapy

**Fig. 7:**
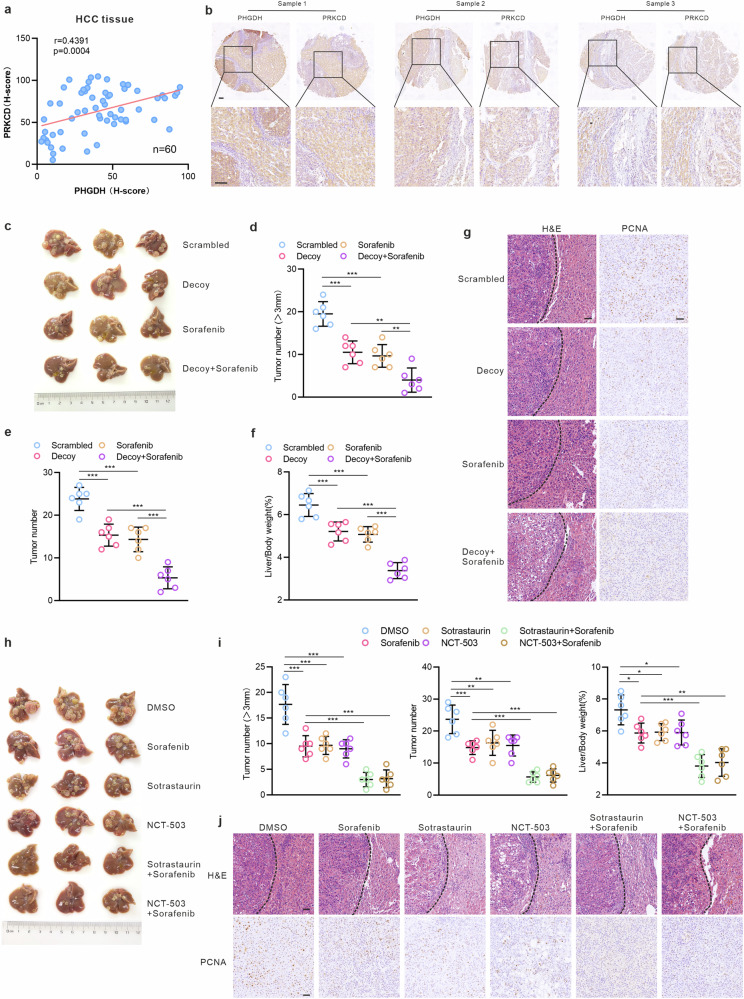
Targeting RBP function of PHGDH as a novel strategy for HCC combination therapy. Correlation analysis (**a**) and representative IHC images (**b**) of PHGDH and PRKCD protein level in clinical HCC tissues (*n* = 60 per group). All markers within the same sample were stained on consecutive sections. Scale bar = 100 μm. **c** Representative appearance of liver tumors under treatments as shown (*n* = 6 per group). **d**, **e** Quantification of number of liver tumors in mice. **f** Liver/body weight of hydrodynamic injection mice with combined treatment by decoy and sorafenib. **g**, Representative IHC images of H&E and PCNA in hydrodynamic injection mice, scale bar = 50 μm. **h** Representative appearance of liver tumors under the treatment shown in the figure (*n* = 6 per group). **i** Quantification of number of liver tumors and liver/body weight of hydrodynamic injection mice. **j** Representative IHC images of H&E and PCNA in hydrodynamic injection mice, scale bar = 50 μm. *P*-values were derived from one-way ANOVA followed by Tukey’s test. Data are represented as the mean ± SD, *P < 0.05, **P < 0.01, ***P < 0.001

To delve deeper into the clinicopathological significance and prognostic value of our results in HCC, we investigated the association between PHGDH, PRKCD and the clinical prognosis of HCC using the Cancer Genome Atlas database (TCGA). The results revealed a strong association between elevated PHGDH/PRKCD expression and poor prognosis in HCC, with patients showing co-upregulation of both genes exhibiting the poorest prognosis (supplementary Fig. 7a–c). Notably, PHGDH exhibited a significant and positive correlation with PRKCD (supplementary Fig. 7d). Consistent with TCGA database findings, the protein levels of PHGDH were significantly and positively associated with PRKCD in HCC tissues (Fig. 7a, b). Additionally, PHGDH and PRKCD were significantly positively correlated with mitophagy-related molecules (supplementary Fig. 7e, f). In summary, clinical patient data indicate that PHGDH and PRKCD play crucial roles in the malignant progression of HCC. Thus, PHGDH and PRKCD may be useful markers of poor prognosis in patients with HCC.

To explore the clinical significance of targeting PHGDH’s non-canonical RBP function in HCC treatment, we assessed the combined effects of decoy oligonucleotides and sorafenib. In the hydrodynamic injection mouse model of HCC, both decoy and sorafenib monotherapies inhibited tumor growth, while their combination showed a significantly enhanced inhibitory effect (Fig. 7c–g). This indicates that combining decoy oligonucleotides, which block PHGDH’s RBP function, with sorafenib is a promising therapeutic strategy. Sotrastaurin (a PRKCD inhibitor) is currently used to treat autoimmune diseases and tumors,^55–58^ but its therapeutic effect on HCC has not yet been observed. We also evaluated the therapeutic synergy of sorafenib with sotrastaurin (a PRKCD inhibitor) and NCT-503 (a canonical PHGDH enzymatic inhibitor). Both combinations reduced tumor burden more effectively than monotherapy, confirming that dual targeting of PHGDH’s enzymatic and RBP functions is a viable combination strategy for HCC (Fig. 7h–j).

## Discussion

**Fig. 8:**
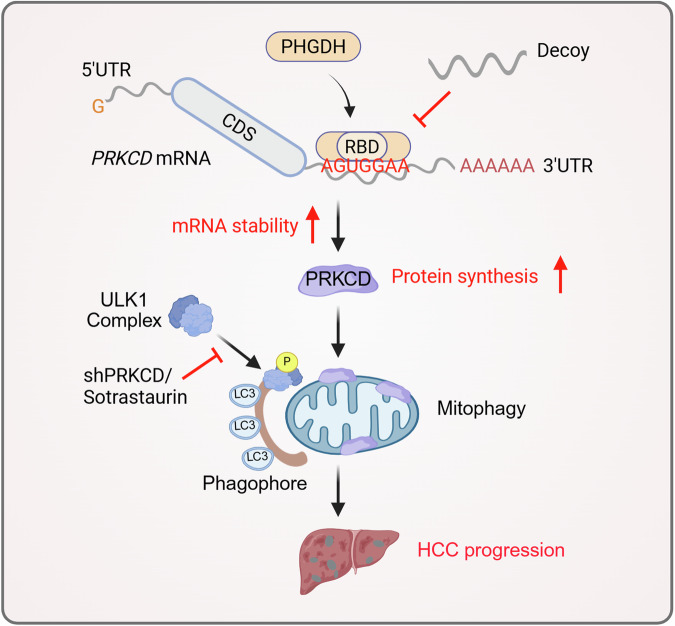
Mechanism diagram of this study. Phosphoglycerate dehydrogenase stabilizes *protein kinase C delta type* mRNA to promote hepatocellular carcinoma progression. Created in BioRender. Cheng, B. (2025) https://BioRender.com/xugxpy6

Changes of RBP expression and function can serve as pivotal oncogenic events. Emerging evidence suggests that metabolic enzymes can function as RBPs that participate in post-transcriptional regulation, potentially amplifying the roles of cancer-driving factors.^59^ The RNA interactomes of numerous canonical human RBPs have been meticulously mapped using the Encyclopedia of DNA Elements (ENCODE) project.^60^ Nonetheless, the association of RNAs with non-canonical RBPs and their implications in cancer and disease remain poorly understood. Our research provides new insights into the non-canonical functions of PHGDH in the post-transcriptional regulation of HCC. PHGDH moonlights as an RBP to recognize and bind a specific motif (AGUGGAA) on the 3’UTR of *PRKCD* mRNA, and PHGDH-*PRKCD* mRNA interaction subsequently promotes *PRKCD* mRNA stability (Fig. 8). Previous studies have suggested that the nucleotide-binding domain of PHGDH exhibits RNA-binding activity.^19,61^ In our study, the precisely identified RBD, which is crucial for facilitating the RNA-binding activity of PHGDH, encompasses this domain. In addition, we identified numerous other metabolic enzymes with potential RNA-binding activity, warranting further investigation into the broader roles of metabolic enzymes as moonlighting RBPs in metabolic regulation and RNA dynamics. Our findings suggest that metabolic dysfunction is deeply embedded in the pathogenesis of HCC and underscore the importance of exploring this interface to uncover novel vulnerabilities in HCC.

As key regulators of post-transcriptional gene expression, RBPs can enhance RNA stability by interacting with specific regions of target transcripts and inducing structural conformations that prevent nuclease-mediated degradation. Our findings suggest that PHGDH may exert similar post-transcriptional control over *PRKCD* mRNA, potentially contributing to its stabilization under certain conditions, such as activated metabolic or stress responses. Future studies aimed at identifying the precise binding sites, in combination with structural approaches such as X-ray crystallography or molecular dynamics simulations, could provide valuable insights into whether PHGDH stabilizes *PRKCD* mRNA through direct structural shielding. Alternatively, RBPs also engage in dynamic regulatory networks by recruiting effector proteins that modulate RNA stability. For example, ENO1 has been shown to facilitate the degradation of *IRP1* mRNA via recruitment of the CCR4-NOT complex component CNOT6.^11^ This raises the possibility that PHGDH, beyond stabilizing *PRKCD* mRNA directly, may also participate in broader RNA regulatory circuits by interacting with decay or stabilization factors. Through analysis of protein interaction databases and Co-IP assays, we identified an interaction between PHGDH and IGF2BP3. This interaction enhances the ability of PHGDH to modulate *PRKCD* mRNA stability. As a regulator of mRNA stability, IGF2BP3 forms stable RNA-protein complexes by specifically targeting the 3’UTR of the mRNA, protecting them from degradation by ribonucleases and thus enhancing their stability.^62,63^ Notably, our results demonstrate that PHGDH still significantly promotes *PRKCD* mRNA stability even upon IGF2BP3 depletion, underscoring a direct regulatory role of PHGDH as an RNA-binding protein. However, additional experimental validation is critical to clarify the regulatory mechanisms of PHGDH, IGF2BP3 and *PRKCD* mRNA.

PHGDH is crucial for mitochondrial function due to its pivotal role in serine synthesis. PHGDH suppression reduces NADPH levels, increases ROS levels and causes mitochondrial dysfunction.^64^ Additionally, PHGDH can influence mitochondrial function through non-enzymatic mechanisms. Subcellular localization of PHGDH within the mitochondrial inner membrane can enhance the translation of mitochondrial DNA, thereby optimizing the efficiency of mitochondrial nucleoid circulation.^24^ Our study reveals a notable correlation between the transcriptional regulation of PHGDH and mitochondrial quality control. PHGDH is pivotal in promoting the formation of autophagy initiation complexes via PRKCD, which ultimately promotes PRKN-independent mitophagy and inhibits apoptosis. Integrating prior findings with those of our study confirms that PHGDH contributes to the mitochondrial function and homeostasis through both conventional enzymatic and non-enzymatic activities. Enhanced mitophagy aids in clearing damaged mitochondria, thus diminishing the release of apoptotic signaling factors like cytochrome C, consequently inhibiting apoptosis and promoting tumor progression.^65^ Our findings align with prior research, indicating that PHGDH suppresses apoptosis and promotes tumor progression. Additionally, under conditions of glucose deprivation, decreased levels of the glycolytic metabolite 3-phosphoglycerate facilitate the binding of PHGDH to p53, thereby activating apoptosis.^66^ These findings indicate that PHGDH multifunctionality is pivotal for tumor progression. Moreover, PHGDH potentially governs a broader array of target genes beyond PRKCD, such as NRAS and STK11. Thus, subsequent work is imperative to unravel the intricate mechanisms by which PHGDH modulates diverse target genes and their consequential implications for tumor progression.

As RBPs interact with and regulate multiple genes involved in the initiation and progression of tumors, RNA interference-based oligonucleotides targeting RBPs are a potential strategy for effectively suppressing their RNA-binding activity while preserving other biological functions.^53,54^ For example, decoy oligonucleotides designed against splicing factors such as RBFOX1/2, SRSF1, and PTBP1 have been shown to specifically bind and inhibit their splicing functions and biological activities both in vitro and in vivo.^53^ In addition, tailored RNA decoy oligonucleotides featuring the CAUC sequence have been shown to effectively target YB-1, inhibiting its RNA-binding activity and suppressing tumor growth in vitro and in vivo.^54^ Similarly, our results reveal that RNA decoy oligonucleotides targeting PHGDH substantially suppress PHGDH-mediated mitophagy and HCC progression. Moreover, the combination of these decoy oligonucleotides with sorafenib significantly enhances the inhibitory effect on HCC tumor growth. Compared to conventional drug design, RNA decoy oligonucleotides offer the advantage of directly targeting the RNA-binding sites of proteins, thereby providing higher specificity and selectivity. Since RNA decoys do not interfere with other biological activities of the proteins, they reduce the likelihood of adverse effects and side effects, making them as a safer and more effective therapeutic option for cancer treatment. Considering the recent approval of certain oligonucleotide drugs for clinical use^67^ and the absence of clinically available drugs targeting PHGDH,^19,20^ RNA decoy oligonucleotides targeting PHGDH have substantial potential for clinical applications. Additionally, although decoys may bind nonspecifically to nontarget proteins, they primarily function by inhibiting the RNA-binding activity of target proteins. We will further evaluate the specificity of PHGDH-decoy oligonucleotides in future studies.

In summary, we reveal a novel non-classical function of PHGDH. Functioning as an RBP, PHGDH stabilizes PRKCD transcripts to activate mitophagy, ultimately promoting HCC progression. This indicates that post-transcriptional regulation arising from metabolic reprogramming can be hijacked by cancer cells for cancer development advantages.^68,69^ Despite the applicability of the RNA-binding activity of PHGDH to other cancers, targeting this moonlighting function offers a distinctive therapeutic strategy for HCC treatment, especially in patients resistant to conventional treatments. This opens an avenue for further research on the clinical applications of decoy oligonucleotides targeting cancer metabolism.

## Materials and methods

### Cell culture and treatment

PLC/PRF/5 and SK-Hep1 cell lines were obtained from the American Type Culture Collection (ATCC, VA, USA). MHCC-97H, Huh7, HEK293 and HEK293T cell lines were obtained from the Cell Bank of the Chinese Academy of Sciences (Shanghai, China). Cells were cultured in Dulbecco’s Modified Eagle’s Medium (HyClone) supplemented with streptomycin (100 μg/mL, HyClone) and penicillin (100 units/mL, HyClone) and 10% fetal bovine serum in 5% CO_2_ atmosphere at 37 °C.

### Ethics statements

The research involving clinical patient samples received ethical clearance from the Medical Ethics Committee of Chongqing Medical University (permit number: 2023075). Animal experiments were conducted in compliance with standards set by the Institutional Animal Care and Use Committee of Chongqing Medical University (permit number: 20230186). All mice were maintained in a pathogen-free environment at the Laboratory Animal Center of Chongqing Medical University.

### mRNA decay assays

Cells were treated with actinomycin D (5 μg/mL, Selleck, USA) and harvested at the specified time. RNA extraction was performed using TRIzol reagent. Subsequently, the PrimeScript™ RT Reagent Kit with gDNA Eraser was employed to reverse-transcribe 1 μg of RNA into cDNA. Gene-specific primers were utilized in RT-qPCR to quantify mRNA levels and extrapolate mRNA half-life (t_1/2_).^70^

### RNA immunoprecipitation and sequencing

Cells underwent two washes with cold phosphate-buffered saline (PBS) and cross-linked twice with 400 mJ/cm^2^ of 254 nm UV. The cells underwent lysis utilizing a buffer comprising a protease inhibitor cocktail and an RNase inhibitor (100U/ml, MCE, USA) and sonicated at 25% amplitude. Following centrifugation, the cell lysates were subjected to an overnight incubation with matching antibodies and Protein A/G Magnetic Beads at 4 °C. Following washing with RIP washing buffer, proteinase K was added and the samples were incubated at 55 °C with shaking for 30 min to digest the proteins. Following incubation, the tubes were briefly centrifuged, and the supernatant was transferred to a new tube. TRIzol was used for the extraction of total RNA. Target gene enrichment was assessed using RT-qPCR and normalized to the input. Clean data suitable for downstream analyses in RIP sequencing were obtained by employing fastp software to eliminate reads containing poly-N sequences, adapter sequences and low-quality reads from the raw data (version 0.20.1). Ribosomal RNA was removed using SortMeRNA software (version 2.1). Subsequently, clean reads were aligned to the genome using hisat2 (version 2.1.0). Peaks were identified by MACS2 (version 2.2.7.1), and the called peaks from the RIP-seq samples were visualized using IGV software. After annotation of the called peaks using BEDTools (version 2.29.2), de novo and known motifs were identified by HOMER (v4.11.1, http://homer.ucsd.edu/homer/download.html). Using a threshold of *P* < 0.05 and a fold change of >2 or <0.5, differential expression analysis was conducted. Enrichment analyses of Gene Ontology (GO) and KEGG pathways for differentially expressed genes were conducted using R software, employing a hypergeometric distribution. All RIP-seq experimental procedures and subsequent data analysis were performed by LC-Bio Technology Co., Ltd.

### PHGDH expression and purification

Wild-type (NM_006623.4) and RBD deletion mutant (125–166 amino acid) genes were inserted into the pET-28a plasmid. The N-terminal 6×His-tagged pET28a-PHGDH and pET28a-RBD deletion constructs were introduced into BL21 *Escherichia coli* (DE3) through transformation. Culture was incubated at 37 °C until it reached an optical density of approximately 0.6 at 600 nm. Following this, induction was initiated by adding 1 mM isopropyl β-D-1-thiogalactopyranoside and the culture was then incubated overnight at 16 °C. After harvesting the bacteria, the pellets were sonicated to lyse in a lysis buffer composed of 300 mM NaCl, 20 mM Tris-HCl (pH 8.0) and 5% glycerol. The purification of fusion proteins was conducted using an Ni-NTA affinity column (GE Healthcare, USA). The elution and concentration of recombinant proteins were performed using Ultrafree-15 centrifugal filters (Millipore, USA).

### RNA pull-down assay

The cells were lysed, followed by centrifugation to collect the supernatant (or the purified prokaryotic protein was added to the cell lysate for pull-down in vitro). Then, 1 μg of biotin-tagged oligo (dT) (20 T-repeats oligonucleotide)^13^ containing 100 U/ml RNase inhibitor was added and allowed to incubate at 25 °C for a duration of 30 min. Subsequently, the proteins bound to the mRNA were isolated through pull-down using streptavidin agarose beads and subsequently detected via western blot or mass spectrometry (the results of metabolic enzymes enriched in the mass spectrometry data were shown in supplementary Table 1).

### Immunofluorescence

After fixing with 4% paraformaldehyde for 25 min, the cells were treated with 0.2% Triton X-100. Following the blocking non-specific sites with 5% BSA, cells were incubated with mouse anti-PHGDH or rabbit anti-IGF2BP3 at 4 °C overnight. Following two washes with PBS, cells were stained with Alexa Fluor 594 or Alexa Fluor 488 secondary antibodies. DAPI was utilized for nuclear counterstaining. Laser-scanning confocal microscope (Leica Microsystems, Germany) was used to capture immunofluorescence images.

### Immunoprecipitation

PHGDH-HA/ PHGDH-Flag and IGF2BP3-Myc were co-transfected into HEK293 cells. The collected cells were washed with cold PBS and then lysed at 4 °C for 30 min with cold NP-40 lysis buffer (Beyotime Biotechnology, China) supplemented with 1×phosphatase inhibitor cocktail and 1× protease inhibitor. In exogenous immunoprecipitation, the cell lysate was subjected to incubation with anti-HA /anti-Flag /anti-Myc antibody overnight at 4 °C. For endogenous immunoprecipitation, the cell lysate underwent overnight incubation at 4 °C with the specified antibody, followed by a 4 h incubation with Protein A/G Magnetic beads (MedChemExpress, USA) at the same temperature. The beads were then washed with PBST, boiled, and analyzed using Western blot.

### Animal studies

At two weeks of age, C57BL/6 J mice received intraperitoneal injections of DEN (75 mg/kg), afterwards, the mice received repeated intraperitoneal administrations of 10% CCl_4_ (2 ml/kg, twice per week for 12 weeks). At eight weeks of age, mice were injected with pAAV8-TBG-shcontrol or pAAV8-TBG-sh*phgdh* (Gene ID: 236539, OBiO Technology, Corp., Ltd. China) via the tail vein (1.5 × 10^11^ genome copies per mouse). At 28 weeks of age, the mice were euthanized. BALB/c nude mice (male, four weeks old) were used to construct the xenograft implantation models. Specifically, 2 × 10^6^ MHCC-97H cells treated with AdPHGDH or shCon/shPRKCD lentiviruses were suspended in PBS and then injected subcutaneously into nude mice. AdGFP was used as a control. Mice aged 4-6 weeks received intraperitoneal injections of the PRKCD inhibitor sotrastaurin (30 mg/kg, MCE, USA) three times a week. After seven weeks, the mice were euthanized for analysis. In the YAP-induced hydrodynamic injection mouse HCC model, six-week-old C57BL/6 J mice received injections of 50 μg pT3-EF1aH Yap S127A (#86497, Addgene, USA) and 1 μg pCMV (CAT) T7-SB100 plasmids (#34879, Addgene, USA). The plasmid solution, constituting 10% of the mouse’s body weight, was administered intravenously via the tail vein within a period of 5 to 7 seconds.^71^ Sorafenib (30 mg/kg, TargetMol, USA), sotrastaurin (30 mg/kg, HY-10343, MCE, USA) and NCT-503 (40 mg/kg, TargetMol, USA) were injected intraperitoneally into mice three times a week at 8–12 weeks of age.

### Patient-derived xenograft (PDX) mouse HCC model

The patient-derived xenograft (PDX) mouse model was sourced from the BEIJING IDMO. To establish the PDX model, tumor fragments obtained from patients were implanted subcutaneously into 6-week-old male NSG mice (NOD.Cg-*Prkdc*^scid^-*IL2rg*^tm1Wjl^*/SzJ*). When the tumor volume reached approximately 80–120 mm³, the mice were randomly divided into groups, with each group containing 5 mice. In PDX model, intratumoral injections of pAAV8-*shPhgdh* or pAAV8-shcontrol were administered.

### Immunohistological staining

Human and mouse liver tissue sections were pretreated with deparaffinization, hydration and antigen retrieval to optimize antigen detection. After blocking non-specific binding, sections were left to incubate at 4 °C with the respective antibody overnight. On the following day, sections were subjected to incubation with a secondary antibody, visualization was carried out using 3,3′-diaminobenzidine. Tissue sections were scanned and quantitatively analyzed using the Pannoramic Scan 250 Flash or MIDI system and Pannoramic Viewer 1.15.2 (3DHISTECH Kft., Hungary). Quantitative scoring criteria for the tissue sections were applied as previously described in ref.^72^

### Statistical analysis

GraphPad Prism 8.0 (GraphPad Software, Inc.)^73^ used to perform statistical analyses. Experimental data, comprising at least three biological replicates, are presented as mean ± standard deviation (SD). Two-tailed Student’s *t*-tests, either unpaired or paired, were utilized to compare two groups, while multiple groups compared by one-way ANOVA. The Kaplan-Meier method was utilized to assess overall survival, followed by analysis with the log-rank test. Linear correlations were evaluated using Pearson’s correlation coefficient. Statistical significance was defined as *P* < 0.05 (*P < 0.05, **P < 0.01 and ***P < 0.001).

## Supplementary information

Supplementary material-revised

Reporting-summary

Unprocessed Western blots

## Data Availability

All pertinent information is provided within the manuscript and its supplementary materials. All raw and processed RIP-Seq data have been deposited in the Gene Expression Omnibus (http://www.ncbi.nlm.nih.gov/geo) under accession number GSE268485. The mass spectrometry proteomics data have been deposited to the ProteomeXchange Consortium (https://proteomecentral.proteomexchange.org) via the iProX partner repository^74,75^ with the dataset identifier PXD063943. The representative metabolic enzymes enriched by RNA pull-down mass spectrometry were available in supplementary Table 1. The Cancer Genome Atlas (TCGA) PanCancer Atlas dataset cited in the study is accessible through a public repository hosted on the cBioPortal website (https://www.cbioportal.org/).
