# Supplementary material for: Phosphoglycerate dehydrogenase stabilizes protein kinase C delta type mRNA to promote hepatocellular carcinoma progression
Source: Signal Transduct Target Ther. 2025 Jul 18;10:236. doi: 10.1038/s41392-025-02304-w (PMC12274589; doi:10.1038/s41392-025-02304-w)
Supplement: Supplementary file 1 — Supplementary material-revised [file 41392_2025_2304_MOESM1_ESM.docx]

Supplementary Materials for

Phosphoglycerate dehydrogenase stabilizes *protein kinase C, delta* mRNA to promote hepatocellular carcinoma progression

Bin Cheng^#^, Pai Peng^#^, Shi Chen^#^, Rui Liu, Xiaosong Li, Ke Wang, Jing Ma, Kai Wang*, Ni Tang*, Ailong Huang*

Correspondence to: ahuang@cqmu.edu.cn

**This PDF file includes:**

Materials and Methods

Supplementary Figures. S1 to S7

Supplementary Tables. S1 to S4

Materials and Methods

**Adenoviruses and plasmids**

Genes of full-length PHGDH and RBD-deficient mutants were cloned into the pAdTrack-TO4 plasmid (a gift from Dr. T-C He, University of Chicago, USA) to generate adenoviral recombinant AdPHGDH and AdPHGDH RBD deletions via the AdEasy system^74^. The control utilized was AdGFP, an analogous adenovirus expressing solely green fluorescent protein. Truncated PHGDH mutants (SBD1, SBD2+RD, SBD1+NBD, and RD) were subcloned into the pBU-3HA plasmid. Truncated mutants of IGF2BP3 (KH1-2, KH3-4 and RRM1-2) were constructed by our research group in the early stages. The 5'UTR and 3'UTR of PRKCD (NM_006254.4) were amplified from human genome DNA and inserted into pGL3-Basic vector (Promega, USA) using the *KpnI* and *HindIII* sites. shRNA of PHGDH and PRKCD was designed by Life Technologies using the Invitrogen Block-iT RNAi Designer (https://rnaidesigner.thermofisher.com/rnaiexpress/sort.do) and was subsequently cloned into the LentiLox 3.7 vectors. Lentivirus was generated by co-transfecting HEK293T cells with shPHGDH and shPRKCD, envelop plasmid pMD2.G, and packaging plasmid psPAX2 using Lipofectamine 3000 (Invitrogen, USA).

**Cell proliferation and colony formation assays**

Cells were seeded in 96-well plates at a density of 1×10^3^/well for the cell proliferation assay. Cell numbers were monitored for five consecutive days to assess their growth dynamics. In the colony formation assay, cells were distributed in 6-well culture plates at a density of 1×10^3^ cells/well and incubated for 10 days. After fixing with 4% paraformaldehyde, the colonies were stained with crystal violet and then photographed.

**Western blot**

Cell lysis buffer (Beyotime Biotechnology, China) supplemented with a phosphatase inhibitor cocktail (TargetMol, China) and protease (TargetMol, China) was employed to extract total proteins. After centrifugation and denaturation, the protein samples underwent separation via SDS-PAGE and electrically transferred to PVDF membranes. Membranes were then blocked with 5% skim milk and subjected to overnight incubation at 4°C with specific antibodies. The membranes were then incubated with the corresponding secondary antibodies after being washed with TBST. Clarity Western ECL Substrate (Bio-Rad) was utilized for visualizing protein bands.

**Quantitative real-time PCR (RT-qPCR)**

Total RNA was extracted from cells or tissue using TRIzol reagent (Invitrogen, USA). Subsequently, the PrimeScript™ RT Reagent Kit with gDNA Eraser (TaKaRa, Japan) was employed to reverse-transcribe RNA into cDNA according to the manufacturer’s protocol. Specific primers were utilized for RT-qPCR to detect various genes, employing SYBR Green qPCR Master Mix (Bio-Rad, USA). Data normalization was conducted using β-actin as an internal control, and quantification was performed utilizing the 2^−ΔΔCt^ method. Primer sequences can be found in Supplementary Table S2.

**Luciferase reporter assay**

pGL3-PRKCD 5'UTR/3'UTR and Flag-PHGDH/Myc-IGF2BP3 were co-transfected into MHCC-97H and PLC/PRF/5 cells using Lipofectamine 3000 (ThermoFisher Scientific, USA). pRL-TK plasmid served as an internal control. Harvesting of cells was conducted at 36 hours post-transfection, and firefly and Renilla luciferase activities were measured using the Dual-Luciferase Reporter Assay System (Promega, USA).

**Supplementary Figures and Figure legends**


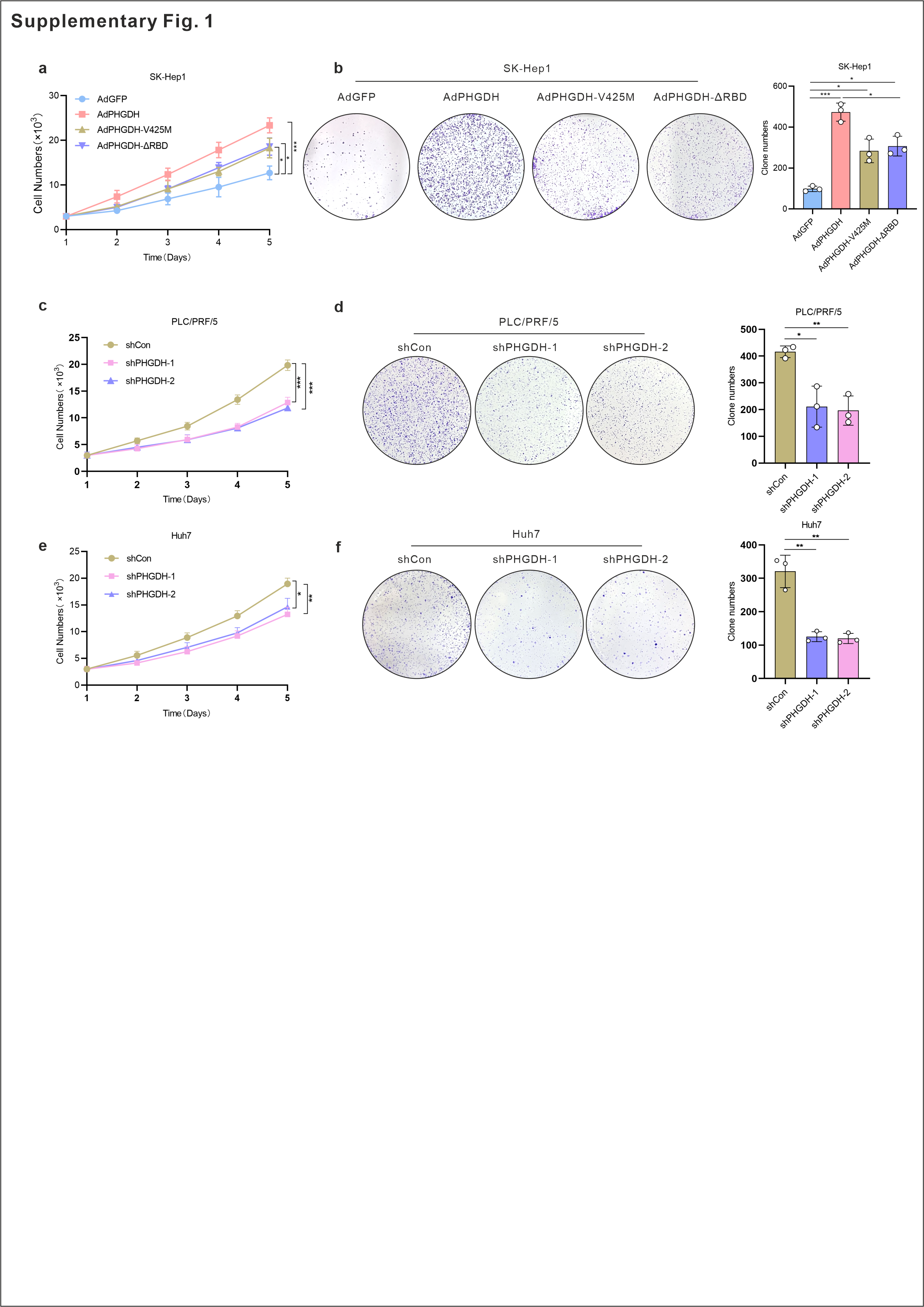


**Supplementary Fig. 1** **|** **PHGDH knockdown suppresses HCC cells proliferation.** **a, b** Cell proliferation curve **(a)** and colony formation **(b)** of SK-Hep1 cells infected with AdPHGDH, AdPHGDH-V425M (enzymatically inactive PHDGH mutant) or AdPHGDH-ΔRBD (n = 3 independent experiments). **c, e,** Cell proliferation ability of PLC/PRF/5 **(c)** and Huh7 **(e)** cells treated with a PHGDH shRNA lentiviral vector (n = 3 independent experiments). **d, f,** Colony formation capacity of PHGDH-knockdown PLC/PRF/5 **(d)** and Huh7 **(f)** cells (n = 3 independent experiments). P-values were derived from one-way ANOVA followed by Tukey’s test. Data are represented as the mean ± SD, *P < 0.05, **P < 0.01, ***P < 0.001.


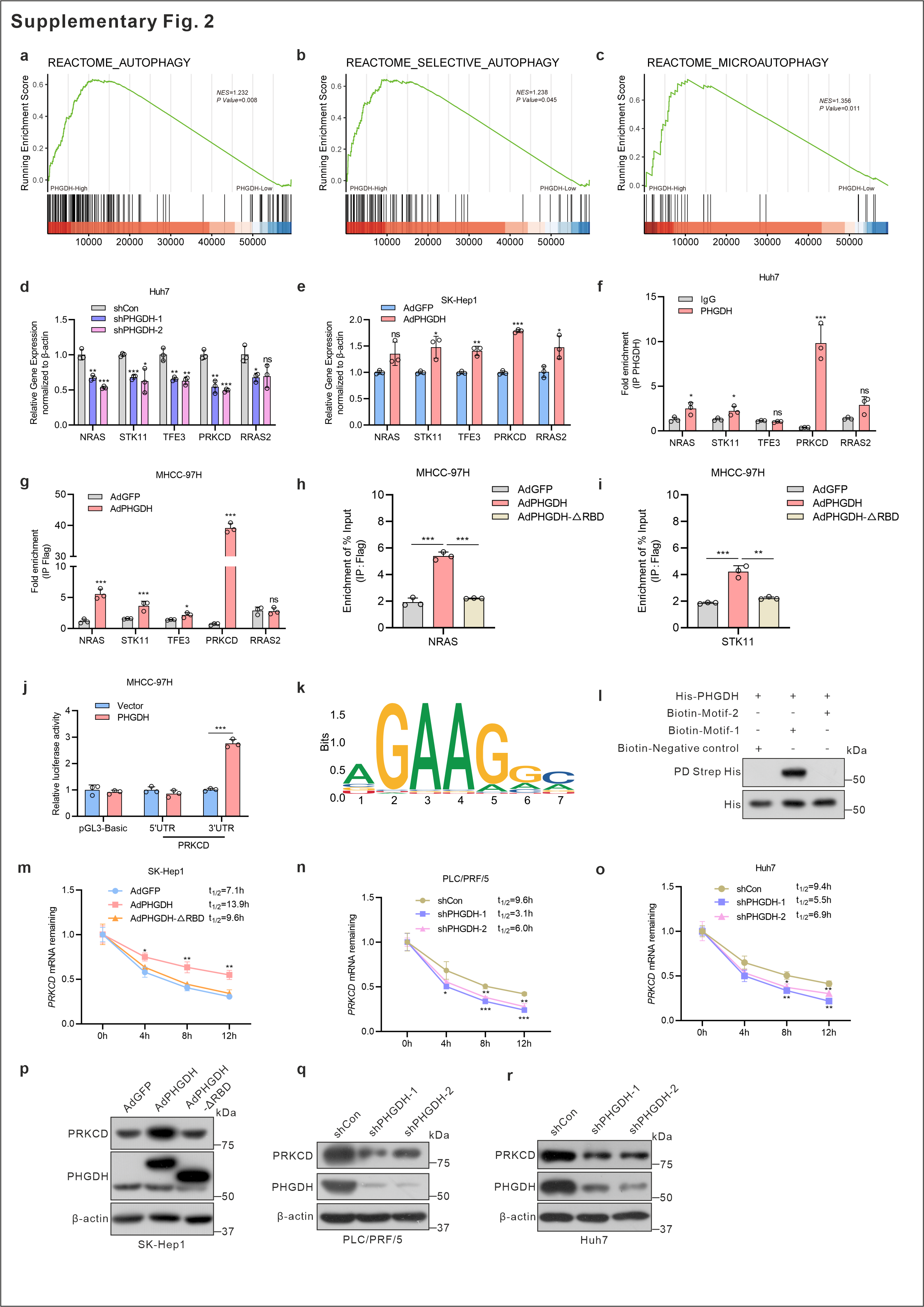


**Supplementary Fig. 2** **| PHGDH enhances the stability of *PRKCD* mRNA and facilitates its protein accumulation. a–c,** Correlation of autophagy (**a**), selective autophagy (**b**) and microautophagy (**c**) with PHGDH by gene set enrichment analysis. **d, e,** Relative mRNA expression of target genes was measured in Huh7 cells treated with a PHGDH shRNA lentiviral vector (**d**) and SK-Hep1 cells infected with AdPHGDH (**e**) (n = 3 independent experiments). **f, g,** RIP-qPCR revealed the association of target transcripts with endogenous PHGDH in Huh7 cells (**f**) and exogenous PHGDH in MHCC-97H cells infected with AdPHGDH (**g**) (n = 3 independent experiments). **h, i,** RIP-qPCR reveals the interaction between NRAS (**h**) and STK11 (**i**) transcripts with PHGDH in MHCC-97H cells infected with AdPHGDH or AdPHGDH-ΔRBD (n = 3 independent experiments). **j,** Relative luciferase activities of *PRKCD* mRNA 5'UTR or 3'UTR with or without PHGDH expression in MHCC-97H cells. **k,** Candidate Motif-2 sequence of PRKCD recognized by PHGDH. **l,** RNA pull-down assays of purified His-tagged PHGDH protein binding to the candidate biotin-labeled motif-1 and motif-2. **m,** Half-life of *PRKCD* mRNA in SK-Hep1 cells infected with AdPHGDH or AdPHGDH-ΔRBD treated with actinomycin D (5 μg/mL) following the specified times (n = 3 independent experiments). **n, o,** Half-life of *PRKCD* mRNA in PLC/PRF/5 cells (**n**) and Huh7 (**o**) cells treated with a PHGDH shRNA lentiviral vector (n = 3 independent experiments). **p,** Western blot of PRKCD protein in SK-Hep1 cells infected with AdPHGDH and AdPHGDH-ΔRBD. **q, r,** Western blot analysis of PRKCD in PHGHD-knockdown PLC5/PRF/5 (**q**) and Huh7 (**r**) cells. P-values were derived from one-way ANOVA followed by Tukey’s test in (**d, h, i, m, n, o**) and unpaired two-tailed Student’s t-test in (**e, f, g, j**). Data are represented as the mean ± SD, *P < 0.05, **P < 0.01, ***P < 0.001.


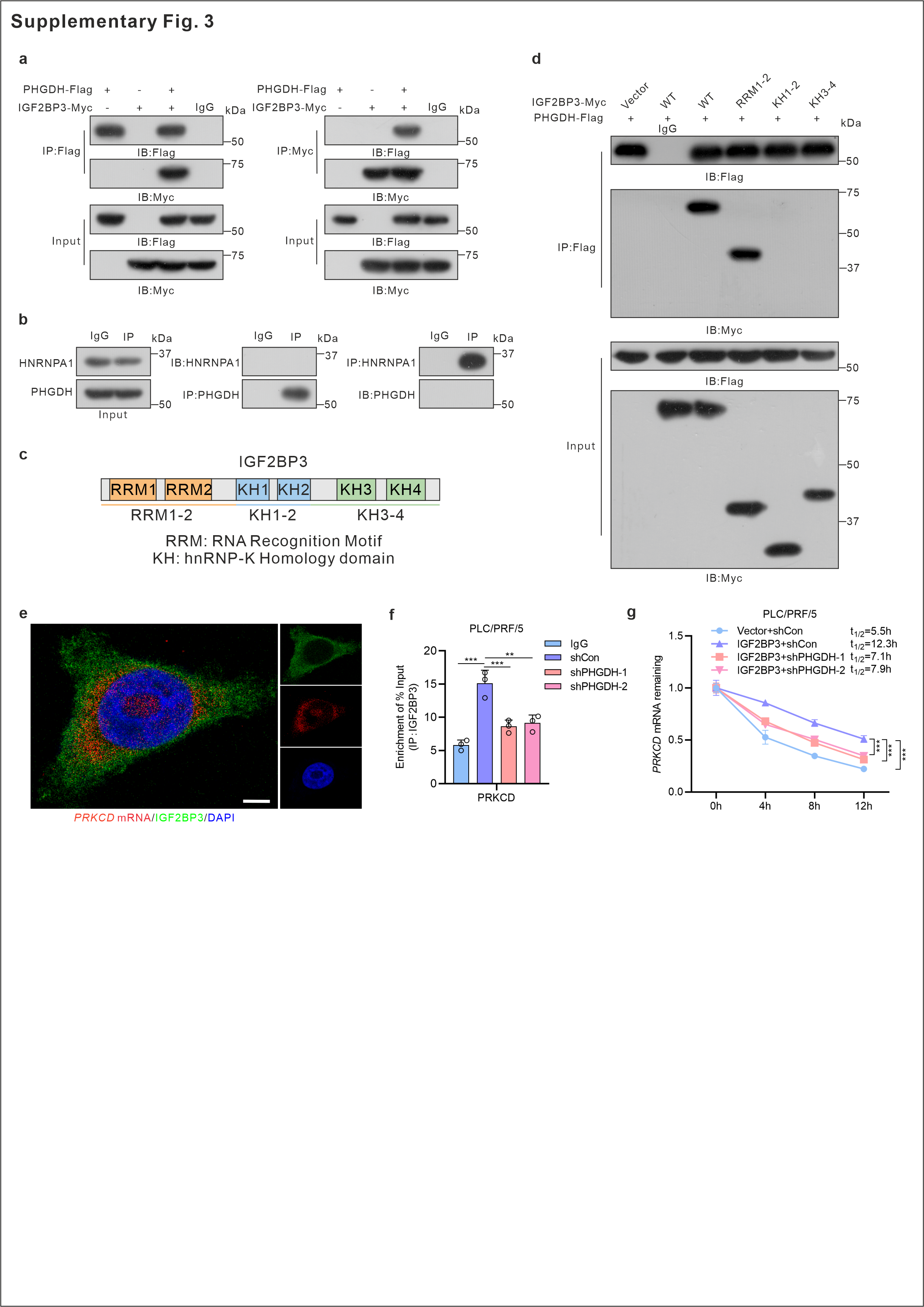


**Supplementary Fig. 3** **| PHGDH interacts with the RRM1-2 domains of IGF2BP3. a,** Co-IP assays of PHGDH-Flag and IGF2BP3-Myc constructs in MHCC-97H cells. **b,** Co-IP assays of endogenous PHGDH and HNRNPA1 in PLC/PRF/5 cells. **c, d,** Co-IP assays of PHGDH and full-length or truncated IGF2BP3 (RRM1-2, KH1-2 or KH3-4) in HEK293 cells. **e,** Florescence in situ hybridization experiment of IGF2BP3 (green) and *PRKCD* mRNA (red) in PLC/PRF/5 cells, Scale bar = 5 μm. **f,** RIP-qPCR revealed the association of PRKCD transcripts with IGF2BP3 in PHGDH-knockdown PLC/PRF/5 cells. **g,** Half-life of *PRKCD* mRNA in PLC/PRF/5 cells overexpressing IGF2BP3. Cells were transfected with PHGDH shRNA lentiviral vector (n = 3 independent experiments). P-values were derived from one-way ANOVA followed by Tukey’s test. Data are represented as the mean ± SD, **P < 0.01, ***P < 0.001.


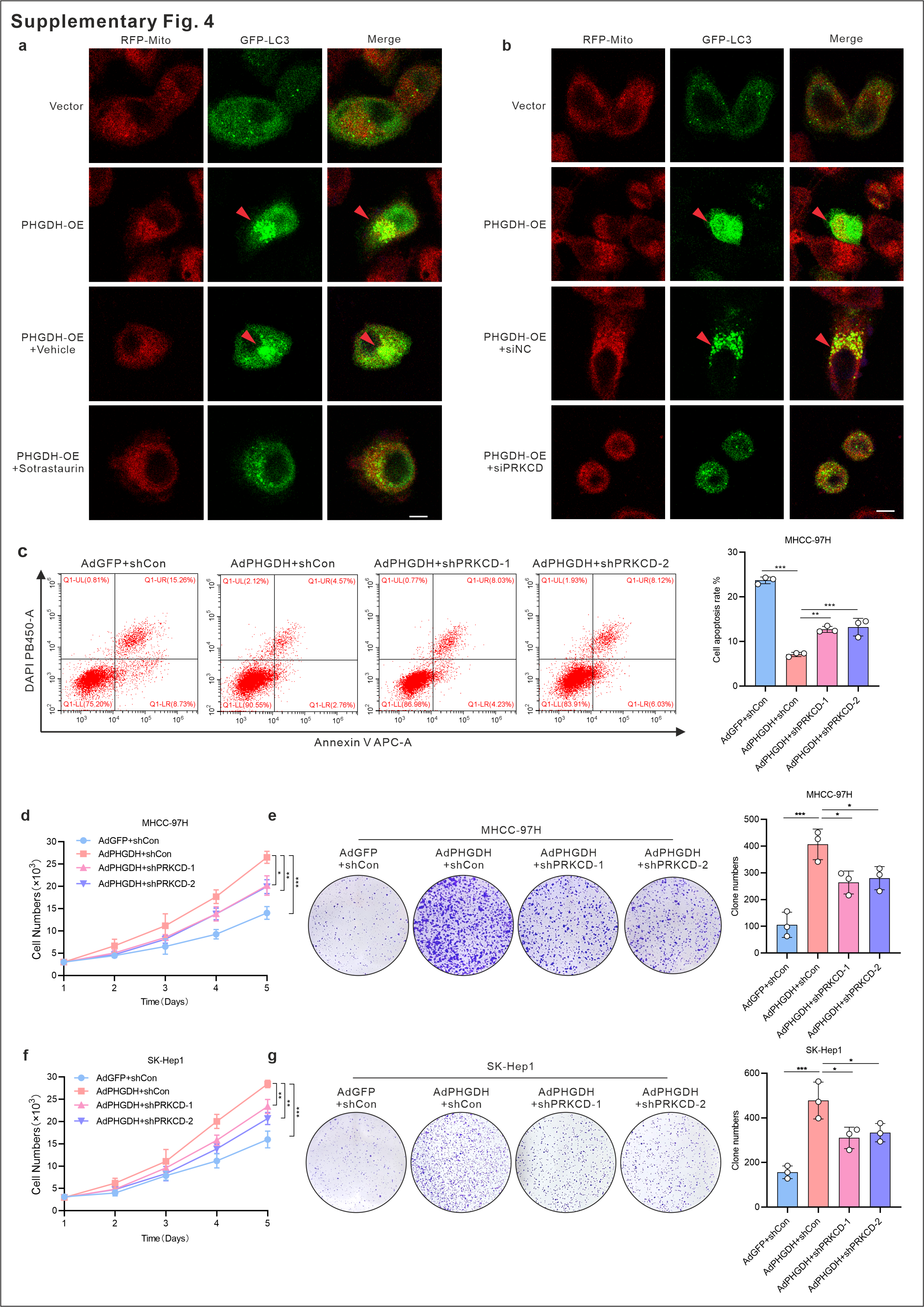


**Supplementary Fig. 4** **| PHGDH activates mitophagy and promotes proliferation of HCC cells through PRKCD. a,** Localization of LC3 (green) in mitochondria (red) in PHGDH-overexpressing MHCC-97H cells supplemented with or without sotrastaurin (2 μM, 24 h). Scar bar = 10 μm. **b,** Localization of LC3 (green) in the mitochondria (red) of PHGDH-overexpressing MHCC-97H cells transfected with PRKCD siRNA. Scar bar = 10 μm. **c,** Flow cytometry was used to detect the effect of PRKCD knockdown on the apoptosis in PHGDH-overexpressing MHCC-97H cells transfected with the PRKCD shRNA lentiviral vector. **d,** **f,** Cell proliferation curves of PHGDH-overexpressing MHCC-97H **(d)** and SK-Hep1 **(f)** cells transfected with the PRKCD shRNA lentiviral vector (n = 3 independent experiments). **e, g,** Colony formation capacity of PHGDH-overexpressing MHCC-97H **(e)** and SK-Hep1 **(g)** cells transfected with the PRKCD shRNA lentiviral vector (n = 3 independent experiments). P-values were derived from one-way ANOVA followed by Tukey’s test. Data are represented as the mean ± SD, *P < 0.05, **P < 0.01, ***P < 0.001.


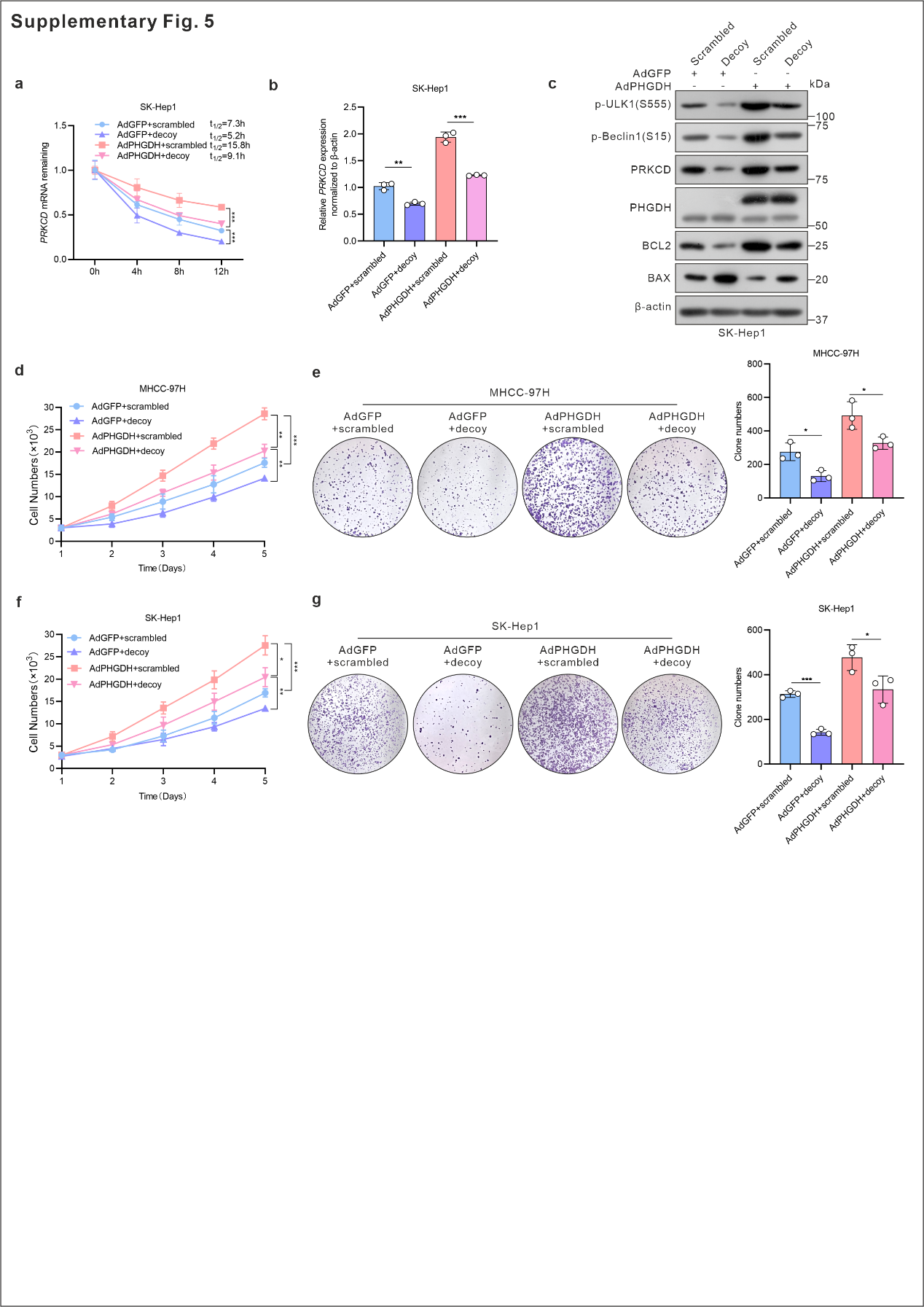


**Supplementary Fig. 5** **| Decoy oligonucleotides inhibit the RNA-binding activity and biological effects of PHGDH. a,** Half-life of *PRKCD* mRNA in PHGDH-overexpressing SK-Hep1 cells treated with PHGDH decoy or scrambled oligonucleotides (n = 3 independent experiments). **b,** Relative mRNA levels of PRKCD were determined in PHGDH-overexpressing SK-Hep1 cells under specified treatment (n = 3 independent experiments). **c,** Western blot of mitophagy and apoptosis markers in PHGDH- overexpressing SK-Hep1 cells treated as specified. **d, f,** Cell proliferation curve of PHGDH- overexpressing MHCC-97H **(d)** and SK-Hep1 **(f)** cells following the specified treatments (n = 3 independent experiments). **e, g,** Colony formation capacity of PHGDH-overexpressing MHCC-97H **(e)** and SK-Hep1 **(g)** cells following the specified treatments (n = 3 independent experiments). P-values were derived from one-way ANOVA followed by Tukey’s test. Data are represented as the mean ± SD, *P < 0.05, **P < 0.01, ***P < 0.001.


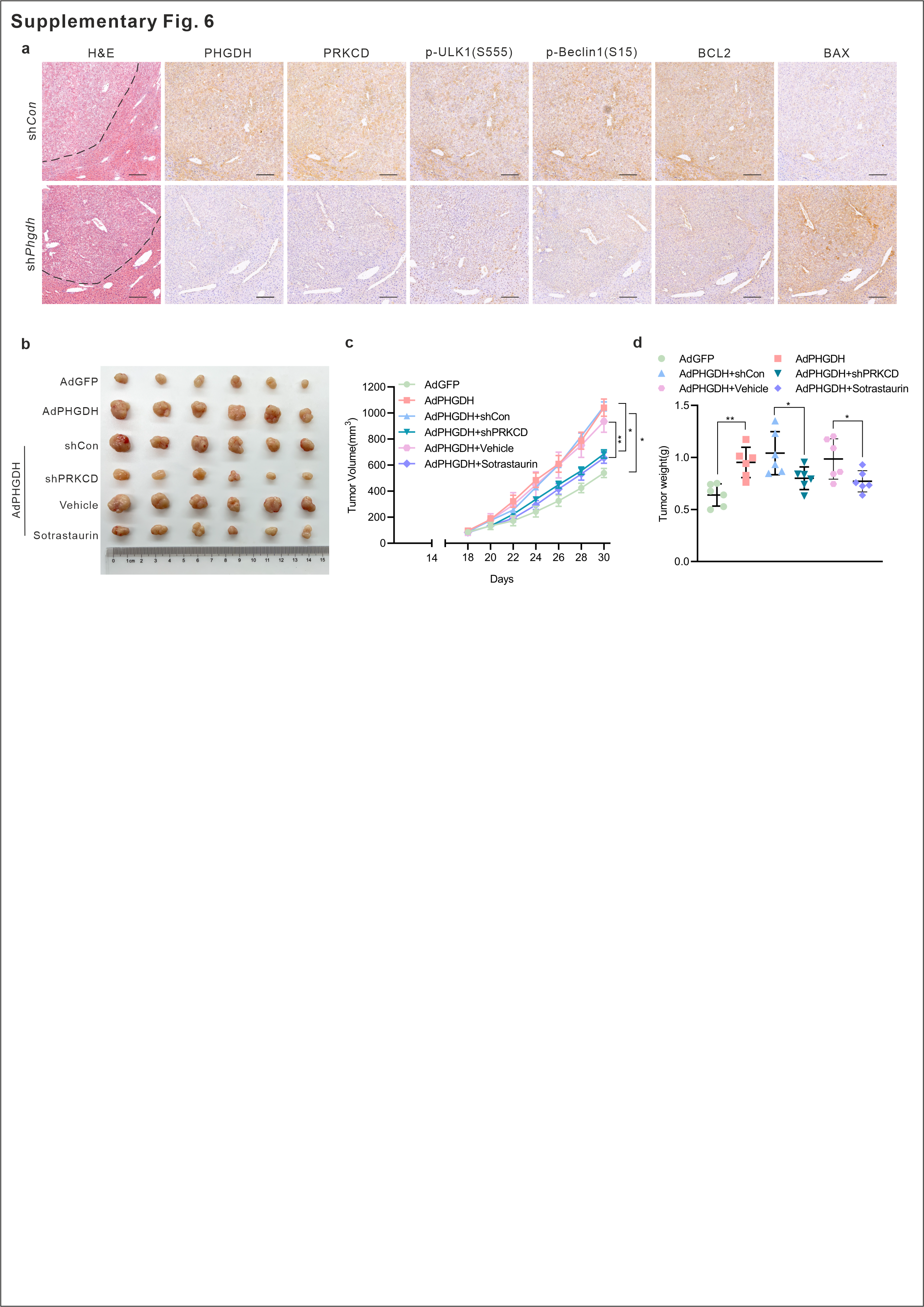


**Supplementary Fig. 6** **| Targeting PRKCD inhibit the proliferation-promoting effects of PHGDH on HCC. a** Representative IHC images of mitophagy and apoptosis makers in DEN/CCl_4_-induced mouse liver samples, Scar bar = 200 μm. All markers within the same experimental group were stained on consecutive sections. **b,** Representative images of subcutaneous tumors. MHCC-97H cells subjected to the indicated treatments were subcutaneously injected into nude mice (n = 6 per group). **c, d,** Tumor volume **(c)** and weight **(d)** of implantation tumors. P-values were derived from one-way ANOVA followed by Tukey’s test. *P < 0.05, **P < 0.01.

**
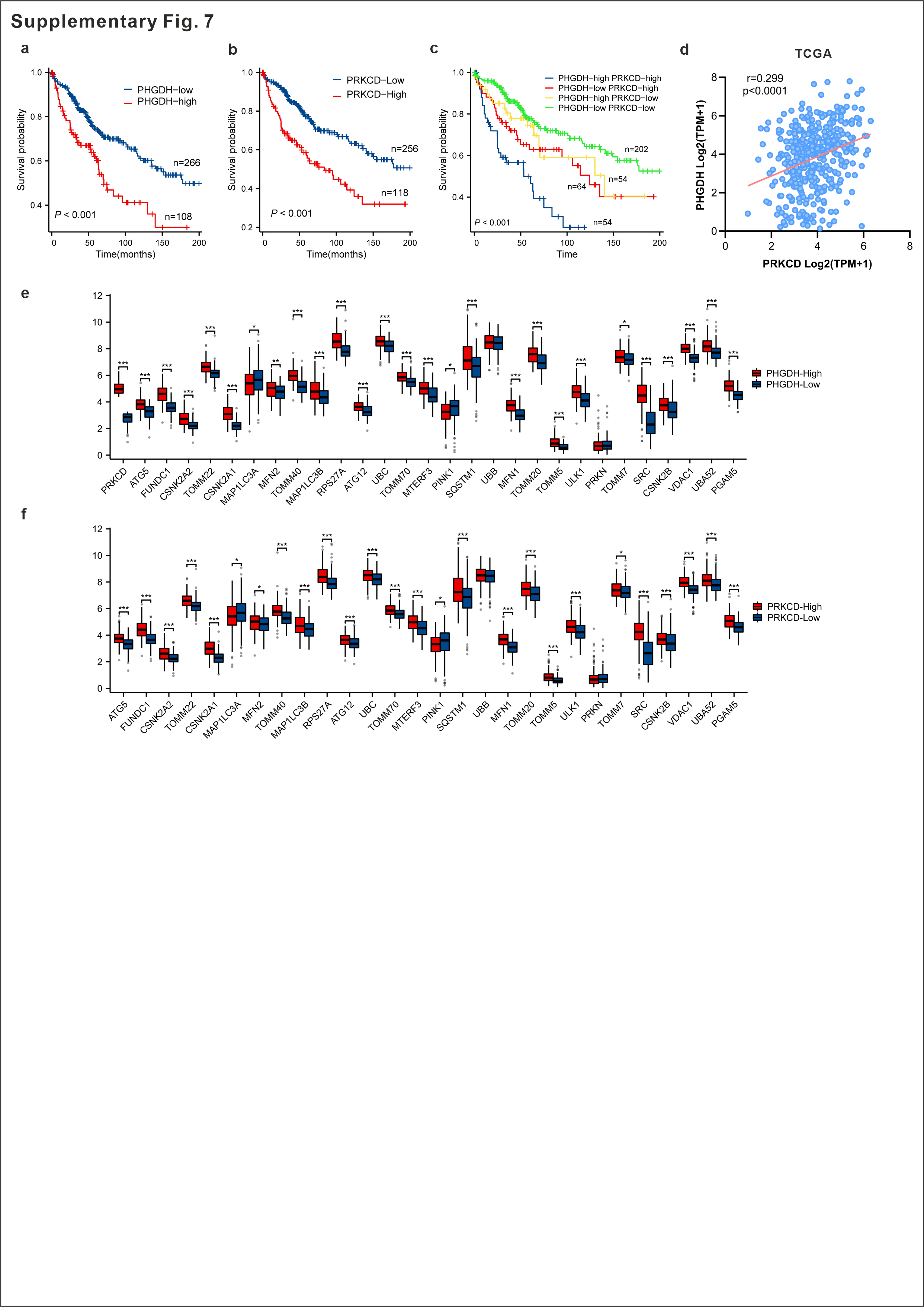
**

**Supplementary Fig. 7** **| PHGDH and PRKCD are closely associated with HCC prognosis and mitophagy molecules. a–c,** Kaplan–Meier survival analysis, based on The Cancer Genome Atlas (TCGA)-HCC dataset (n = 374), were performed to evaluate the overall survival rate among HCC patients with different expression levels of PHGDH **(a)**, PRKCD **(b)** or both PHGDH and PRKCD **(c)**. **d,** Correlation analysis of *PHGDH* and *PRKCD* mRNA level from TCGA. **e, f,** Correlation analysis of PHGDH (**e**), PRKCD (**f**) and mitophagy-related mRNA levels from the TCGA dataset. P-values were derived from one-way ANOVA followed by Tukey’s test in **(a-d)** and unpaired two-tailed Student’s t-test in (**e, f**). Data are represented as the mean ± SD, *P < 0.05, **P < 0.01, ***P < 0.001.

| **Table 1.** **Metabolic enzymes in RNA pull down-MS** | | | | |
| --- | --- | --- | --- | --- |
| GeneName | Accession | Coverage [%] | # Unique Peptides | Abundances (Grouped) |
| PCCB | A0A024RDF4 | 51 | 3 | 2086907141 |
| PC | P11498 | 51 | 49 | 524212896.3 |
| PKM | Q96EP5 | 27 | 7 | 64750630.63 |
| GANAB | P27694 | 23 | 15 | 54663364.56 |
| MCCC2 | A0A087X0X3 | 32 | 23 | 50381375.83 |
| CTPS1 | O43175 | 17 | 8 | 40530036.94 |
| MCCC1 | Q2TSD0 | 35 | 9 | 37799675.38 |
| PYGB | P67809 | 25 | 3 | 19059292.25 |
| NAMPT | A0A7P0TAE9 | 13 | 7 | 14566019.88 |
| NQO1 | Q53F91 | 14 | 10 | 10441789.59 |
| MARS1 | P38159 | 15 | 6 | 7758146.938 |
| UQCRC2 | P17812 | 16 | 8 | 7105774.344 |
| PHGDH | H0YLU7 | 27 | 6 | 6972036.625 |
| GFPT1 | O75874 | 11 | 4 | 6966018.375 |
| IDH3B | H0YDT6 | 13 | 4 | 5263179.438 |
| PCK2 | P54886 | 5 | 4 | 4373811.156 |
| RPN2 | B4DY96 | 9 | 4 | 4199564.313 |
| SUCLA2 | Q96IR1 | 10 | 3 | 4009872.188 |
| DDOST | Q15365 | 8 | 1 | 3710741.875 |
| ALDOA | B4DN41 | 8 | 5 | 3528754.953 |
| HSD17B4 | B4DUC5 | 6 | 4 | 3497554.75 |
| AK4 | Q53FB6 | 7 | 4 | 2790017.156 |
| IDH1 | Q53GR7 | 9 | 5 | 2540528.875 |
| ACSL4 | Q2TAK1 | 4 | 3 | 2343542.25 |
| ASNS | B4DP50 | 10 | 3 | 2227658.938 |
| PYGL | H7C463 | 5 | 3 | 2048858.438 |
| AHCY | A0A024R458 | 5 | 2 | 2012597.125 |
| MRI1 | B7Z9U2 | 8 | 5 | 1994458.906 |
| NDUFS3 | Q15293 | 5 | 1 | 1881361.5 |
| DLAT | P62280 | 9 | 2 | 1808838.875 |
| GLS | D9HTE9 | 7 | 2 | 1751069.063 |
| ATP5F1A | B2R853 | 40 | 1 | 1700011.625 |
| AKR1C1 | Q9Y678 | 6 | 4 | 1592034.719 |
| PCYT1A | C9JH19 | 2 | 1 | 1535116.125 |
| PGD | Q01581 | 8 | 2 | 1494924.281 |
| HMGCS1 | O60701 | 5 | 2 | 1408209.125 |
| PFKL | Q14974 | 2 | 2 | 1319504.531 |
| GCLM | Q12904 | 5 | 1 | 1213498.875 |
| FASN | B4DX93 | 5 | 2 | 1179065.125 |
| NDUFV1 | Q7L0Y3 | 4 | 2 | 1176995.219 |
| MAN1A1 | B2RDN9 | 1 | 1 | 1112263.125 |
| UGDH | Q59GP7 | 4 | 2 | 1097384.469 |
| GSTP1 | H0YHA7 | 8 | 1 | 897614.8125 |
| ALDH3A2 | P51648 | 4 | 2 | 817913.0469 |
| AGK | H7C5E8 | 5 | 1 | 799470.8125 |
| FH | B4DNL7 | 5 | 1 | 709403.875 |
| PCCA | P04350 | 56 | 0 | 602609.6875 |
| MAT2A | P16989 | 10 | 1 | 564091.625 |
| TALDO1 | M0QZK8 | 22 | 2 | 552127.4375 |
| NDUFS2 | Q99832 | 1 | 1 | 539496.3125 |
| GFUS | Q02978 | 5 | 1 | 517810.6563 |
| AGPS | O60930 | 6 | 1 | 451842.1563 |
| SUCLG1 | B0QYT5 | 12 | 1 | 400975.5313 |
| STT3A | B4DSN5 | 2 | 1 | 384980.125 |
| GPI | A0A0J9YYH3 | 8 | 2 | 361893.5625 |
| MDH2 | J3KRX5 | 9 | 1 | 312242.8125 |
| FADS2 | Q63HL4 | 1 | 1 | 296485.625 |
| HSD17B10 | A0A7I2YQD8 | 2 | 1 | 268554.6875 |

| **Table 2. Primer sequences used in this study** | | |
| --- | --- | --- |
| **Name** | **Sense Primer (5'-3')** | **Antisense Primer (5'-3')** |
| **Primer sequences for molecular cloning** | | |
| pAd-Track-TO4-3Flag-PHGDH | TGGGGTACCATGGCTTTTGCAAATCTGC | TCCAAGCTTGAAGTGGAACTGGAAGGCTT |
| pAd-Track-TO4-3Flag-PHGDH-ΔRBD | CAGATTCCCCAGGCGTTTGGGATGAAGACTATAGG | ATAGTCTTCATCCCAAACGCCTGGGGAATCTGC |
| pAd-Track-TO4-3Flag-PHGDH V425M | AATGCCTCCTGGCCATGGCCCTGGCAGG | CTGCCAGGGCCATGGCCAGGAGGCATTC |
| pGL3-Basic-PRKCD-5' UTR | CGGGGTACCGCACTTCCGTGTGCCG | CCCAAGCTTGGTGGGGCCTGCAGTG |
| pGL3-Basic-PRKCD-3' UTR | CGGGGTACCGGTTCCTGGACAGATCAGG | CCCAAGCTTTTGGTACTATTTGGTGTTTATTT |
| pBudCE4.1-3HA-PHGDH-ΔRD | TGGGGTACCATGGCTTTTGCAAATCTGC | CCGCTCGAGTCCCCCGTGAGAGATTTCCC |
| pBudCE4.1-3HA-PHGDH-ΔSBD1 | TGGGGTACCATGAGTGCCGCAGAACTCACTT | CCGCTCGAGTCGAAGTGGAACTGGAAGGCTT |
| pBudCE4.1-3HA-PHGDH-ΔSBD2+RD | TGGGGTACCATGGCTTTTGCAAATCTGC | CCGCTCGAGTCGGCACCCAGGTGGGGAC |
| pBudCE4.1-3HA-PHGDH-ΔSBD1+NBD | TGGGGTACCATGAGCACCAAGGAGGCTCAGA | CCGCTCGAGTCGAAGTGGAACTGGAAGGCTT |
| pET-28a-PHGDH | TGCGGATCCGCTTTTGCAAATCTGCG | TCCAAGCTTTTAGAAGTGGAACTGGAAGG |
| **Primer sequences for knockdown** | | |
| shPHGDH-1 | TGCAGGACTGTGAAGGCCTTATTTCAAGAGAATAAGGCCTTCACAGTCCTGCTTTTTTC | TCGAGAAAAAAGCAGGACTGTGAAGGCCTTATTCTCTTGAAATAAGGCCTTCACAGTCCTGCA |
| shPHGDH-2 | TGCTGAATGACAACACCTTTGCTTCAAGAGAGCAAAGGTGTTGTCATTCAGCTTTTTTC | TCGAGAAAAAAGCTGAATGACAACACCTTTGCTCTCTTGAAGCAAAGGTGTTGTCATTCAGCA |
| shPRKCD-1 | TGCAAGAAGAACAATGGCAAGGTTCAAGAGACCTTGCCATTGTTCTTCTTGCTTTTTTC | TCGAGAAAAAAGCAAGAAGAACAATGGCAAGGTCTCTTGAACCTTGCCATTGTTCTTCTTGCA |
| shPRKCD-2 | TGCTGCCATCCACAAGAAATGCTTCAAGAGAGCATTTCTTGTGGATGGCAGCTTTTTTC | TCGAGAAAAAAGCTGCCATCCACAAGAAATGCTCTCTTGAAGCATTTCTTGTGGATGGCAGCA |
| **Primer sequences for real-time PCR** | | |
| NRAS | GAAAAGCGCACTGACAATCC | CACCACACATGGCAATCCC |
| STK11 | GAGCTGATGTCGGTGGGTATG | CACCTTGCCGTAAGAGCCT |
| TFE3 | ATTGGGTCCAGCTCGAAAA | TGATAGCTGGGGTAGCCACT |
| RRAS2 | AGGAGAAGTACCGGCTCGT | CGCCAGTCCTCATATACTGTTC |
| PRKCD | CCTGACTATATCGCCCCTGA | GTCCTTGGACTCCTTGGTGA |
| β-actin | AGGCCAACCGCGAGAAGATGACC | GAAGTCCAGGGCGACGTAGCAC |

| **Table 3. Antibodies used for Western blot, IP, IF and IHC** | | | |
| --- | --- | --- | --- |
| **Antibodies** | **Source** | **Antibody dilution** | **Cat no.** |
| Mouse anti-Flag | Sigma, Germany | 1:4000 for WB | F3165 |
| Mouse anti-β-actin | ZSGB-BIO, China | 1:2000 for WB | TA-09 |
| Mouse anti-His | Abcam, USA | 1:1000 for WB | ab18184 |
| Goat anti-mouse, secondary | Abcam, USA | 1:10000 for WB | ab6789 |
| Rabbit anti-PHGDH | Proteintech, China | 1:1000 for WB; 1:200 for IHC; 1:500 for IF | 14719-1-AP |
| Rabbit anti-PRKCD | Proteintech, China | 1:1000 for WB; 1:200 for IHC | 19132-1-AP |
| Rabbit anti-HNRNPA1 | Proteintech, China | 1:1000 for WB; | 11176-1-AP |
| Rabbit anti-IGF2BP3 | Proteintech, China | 1:1000 for WB; 1:500 for IF | 14642-1-AP |
| Mouse anti-β-actin | ZSGB-BIO, China | 1:2000 for WB | TA-09 |
| Goat anti-rabbit, secondary | Abcam, USA | 1:5000 for WB | ab6721 |
| Mouse anti-Myc | CellSignalingTechnology, USA | 1:2000 for WB | 2276 |
| Mouse anti-HA | CellSignalingTechnology, USA | 1:2000 for WB | 3724 |
| Rabbit anti-p-ULK1 (S555) | Cell Signaling Technology, USA | 1:1000 for WB; 1:100 for IHC | 5869 |
| Rabbit anti-p-Beclin1 (S15) | Cell Signaling Technology, USA | 1:1000 for WB; 1:100 for IHC | 84966 |
| Rabbit anti-BCL2 | Cell Signaling Technology, USA | 1:1000 for WB; 1:100 for IHC | 4223 |
| Rabbit anti-BAX | Cell Signaling Technology, USA | 1:1000 for WB; 1:100 for IHC | 2772 |
| Goat anti-Rabbit IgG, Alexa Fluor ® 488 | Invitrogen, USA | 1:500 for IF | A-11034 |
| Goat anti-Mouse IgG, Alexa Fluor ®594 | Invitrogen, USA | 1:500 for IF | A-21125 |

| **Table 4: Chemicals, Critical Commercial Assays and Experimental Models.** | | |
| --- | --- | --- |
| **Name** | **Source** | **Identifier** |
| **Chemicals** | | |
| DMEM | HyClone | SH30243.01 |
| Mayer’s Hematoxylin Stain Solution | Solarbio | G1080 |
| Penicillin-Streptomycin Solution | HyClone | SV30010 |
| Diethylnitrosamine (DEN) | Sigma-Aldrich | N0756 |
| Carbon tetrachloride (CCl_4_) | Macklin | C805332 |
| 2×Taq Master Mix | Novoprotein | E005-01A |
| NP-40 lysis buffer | Beyotime Biotechnology | P0013F |
| Cell lysis buffer for Western | Beyotime Biotechnology | P0013 |
| Pierce™ streptavidin agarose beads | Thermo Fisher Scientific | #20347 |
| TRIzol reagent | Invitrogen | 15596026 |
| cOmplete™, EDTA-free Protease Inhibitor Cocktail Tablets | Roche Diagnostics GmbH | 04693132001 |
| Actinomycin D | Selleck | S8964 |
| DAPI | Roche Diagnostics GmbH | 10236276001 |
| TRIzol™ Reagent | Invitrogen | 15596026 |
| iTaq™ Universal SYBR® Green Supermix | Bio-Rad | 1725121 |
| RNase inhibitor | MedChemExpress | HY-K1033 |
| Protein A/G Magnetic beads | MedChemExpress | HY-K0202 |
| Sotrastaurin | MedChemExpress | HY-10343 |
| Sorafenib | TargetMol | T0093L |
| Lipofectamine 3000 | ThermoFisher | L3000015 |
|  | | |
| **Critical Commercial Assays** | | |
| Magna RIP™ RNA-Binding Protein Immunoprecipitation Kit | Millipore | # 17-701 |
| PrimeScript™ RT Reagent Kit | TaKaRa | RR047A |
| Dual-Luciferase Reporter Assay System | Promega | E1910 |
|  | | |
| **Experimental Models** | | |
| BALB/c nude mice (male) | Experimental Animal Center of Chongqing Medical University | N/A |
| C57 BL/6J mice (male) | Experimental Animal Center of Chongqing Medical University | N/A |
