# Supplementary material for: Phosphoglycerate dehydrogenase stabilizes protein kinase C delta type mRNA to promote hepatocellular carcinoma progression
Source: Signal Transduct Target Ther. 2025 Jul 18;10:236. doi: 10.1038/s41392-025-02304-w (PMC12274589; doi:10.1038/s41392-025-02304-w)

# Full unedited gels for Fig.1d

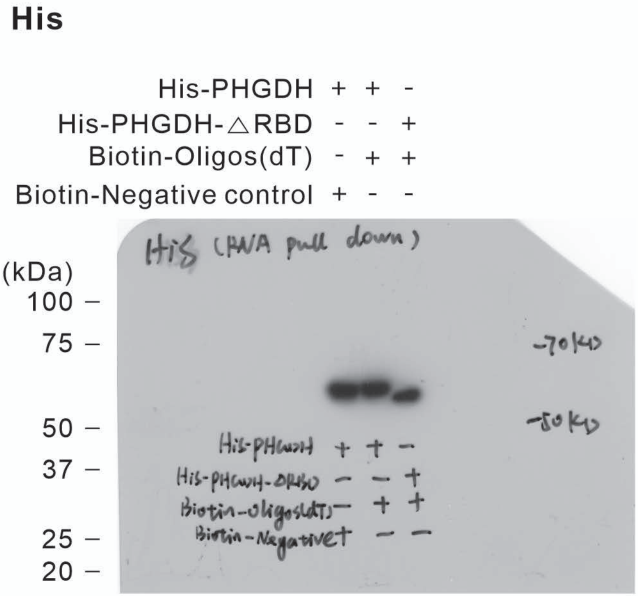


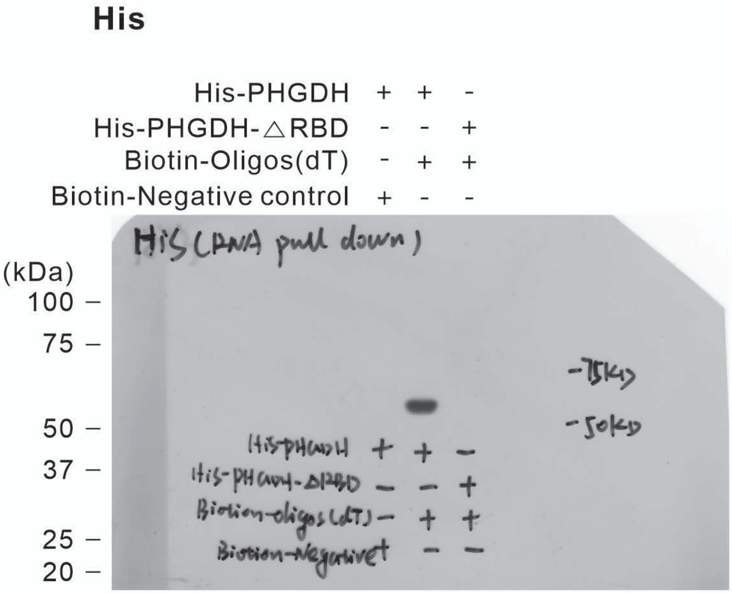

# Full unedited gels for Fig.2i

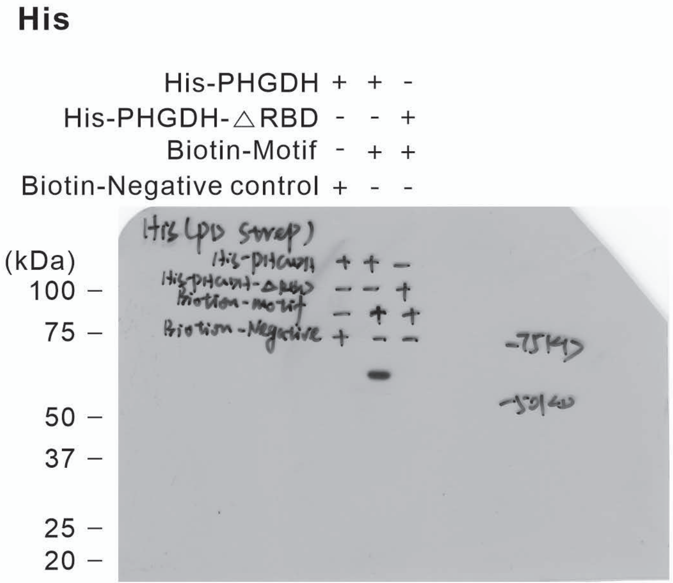


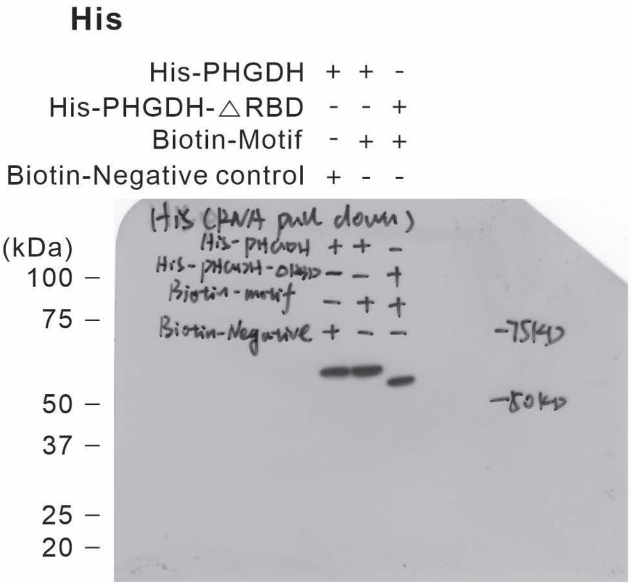

**Full unedited gels for Fig.2l**

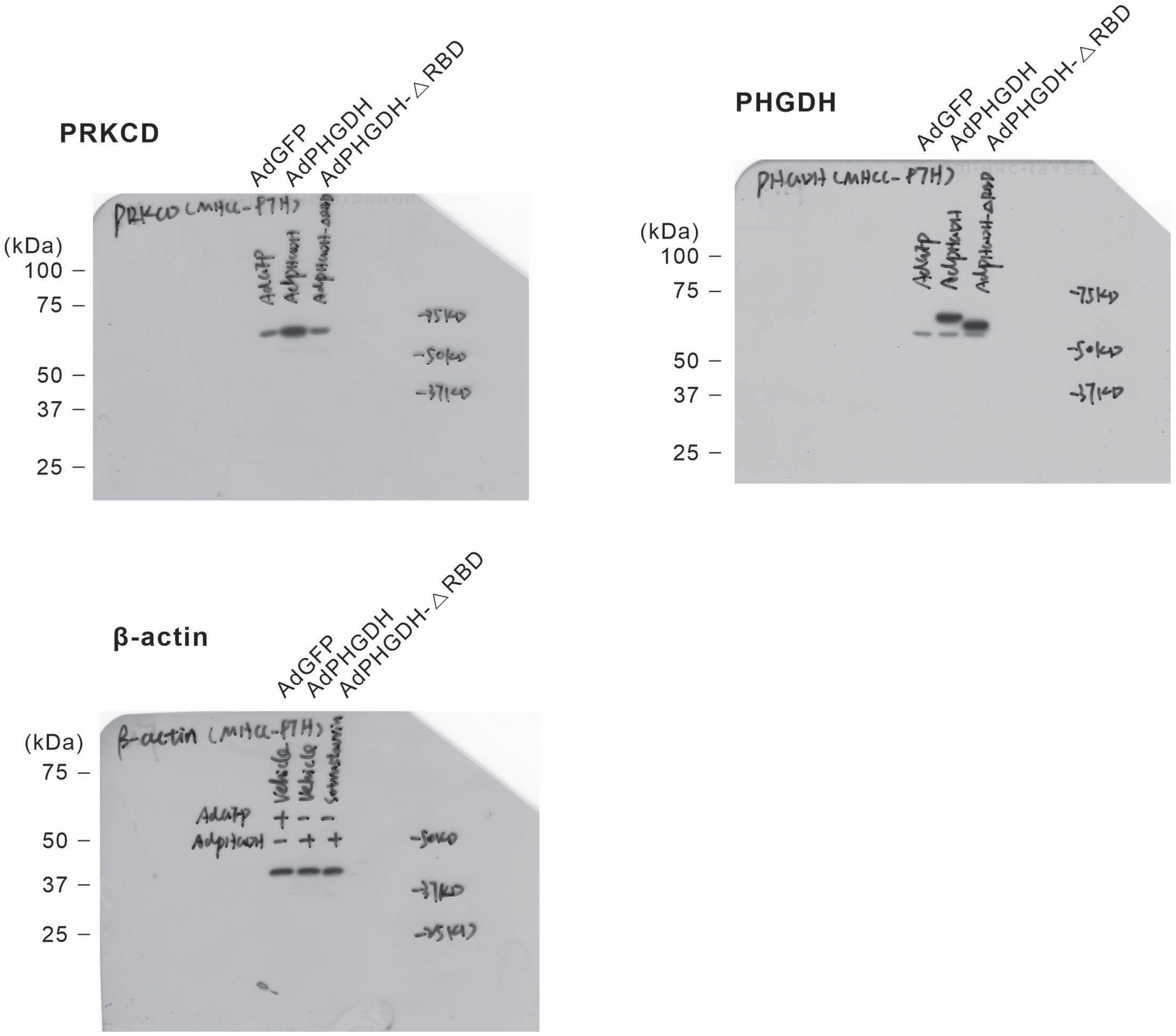

Full unedited gels for Fig.3a

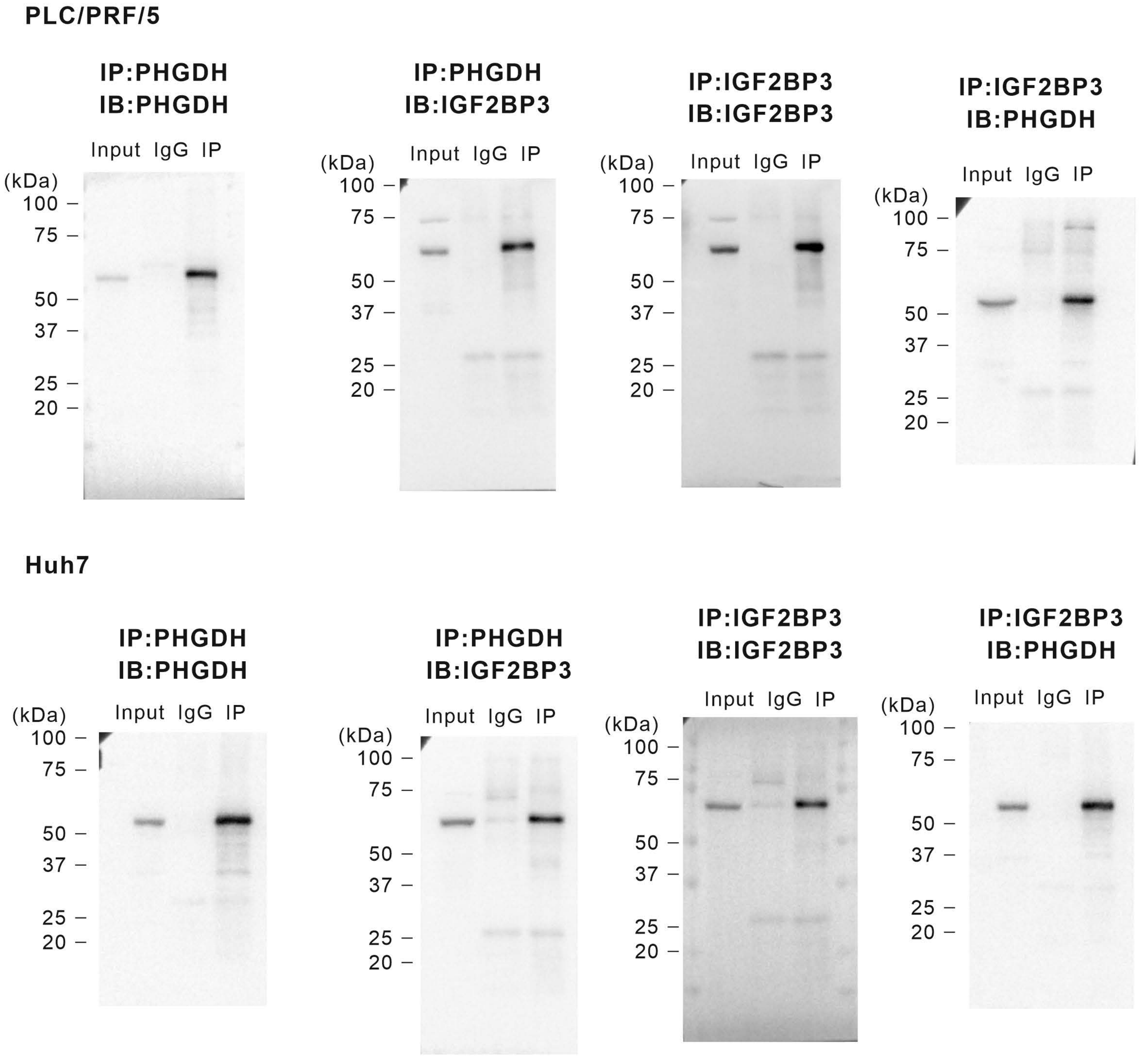

Full unedited gels for Fig.3b

**IP:Flag**
**IB:Myc**

| | | | | |
|---|---|---|---|---|
| PHGDH-Flag | + | - | + | |
| IGF2BP3-Myc | - | + | + | IgG |

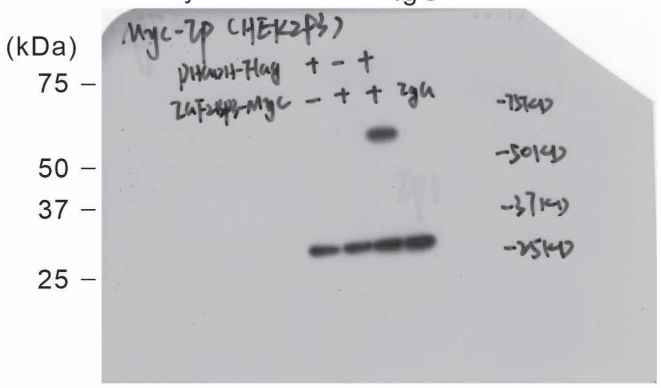


**IP:Flag**
**IB:Flag**

| | | | | |
|---|---|---|---|---|
| PHGDH-Flag | + | - | + | |
| IGF2BP3-Myc | - | + | + | IgG |

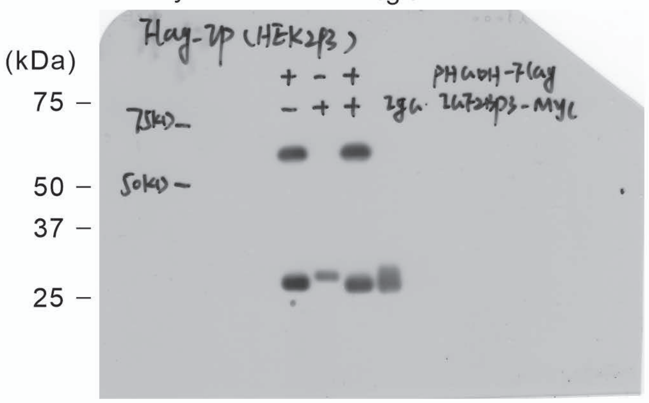


**Input**
**IB:Flag**

| | | | | |
|---|---|---|---|---|
| PHGDH-Flag | + | - | + | |
| IGF2BP3-Myc | - | + | + | IgG |

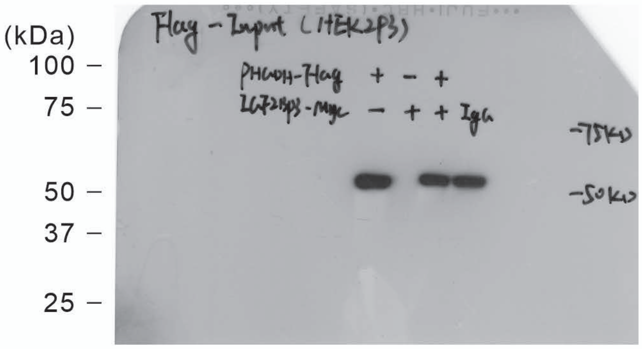


**Input**
**IB:Myc**

| | | | | |
|---|---|---|---|---|
| PHGDH-Flag | + | - | + | |
| IGF2BP3-Myc | - | + | + | IgG |

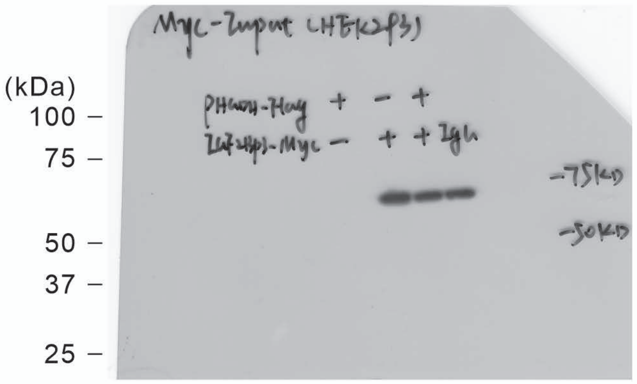


**IP:Myc**
**IB:Flag**

| | | | | |
|---|---|---|---|---|
| PHGDH-Flag | + | - | + | |
| IGF2BP3-Myc | - | + | + | IgG |

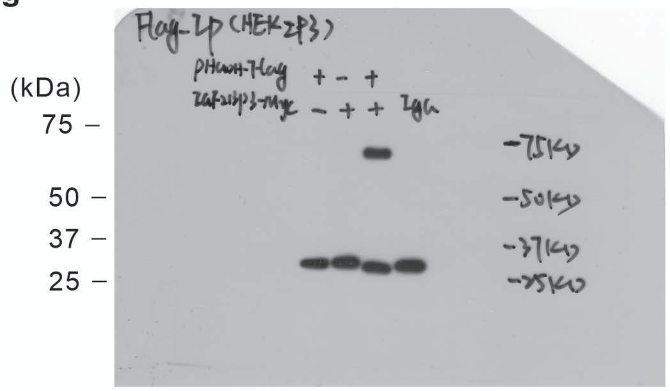


**IP:Myc**
**IB:Myc**

| | | | | |
|---|---|---|---|---|
| PHGDH-Flag | + | - | + | |
| IGF2BP3-Myc | - | + | + | IgG |

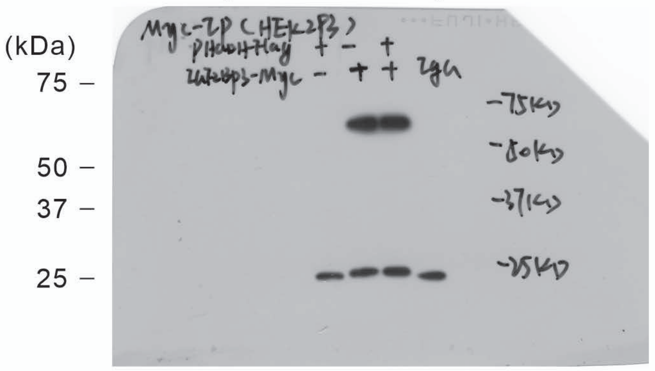


**Input**
**IB:Flag**

| | | | | |
|---|---|---|---|---|
| PHGDH-Flag | + | - | + | |
| IGF2BP3-Myc | - | + | + | IgG |

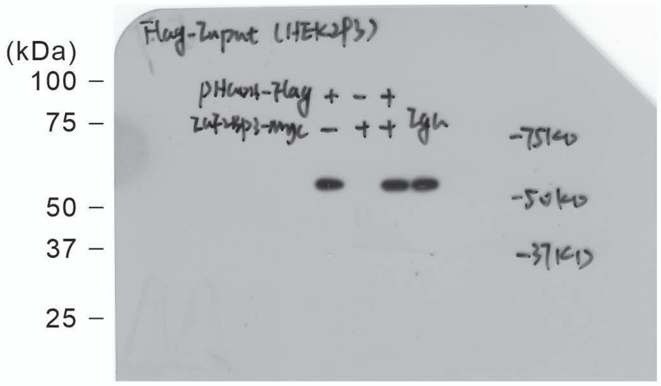


**Input**
**IB:Myc**

| | | | | |
|---|---|---|---|---|
| PHGDH-Flag | + | - | + | |
| IGF2BP3-Myc | - | + | + | IgG |

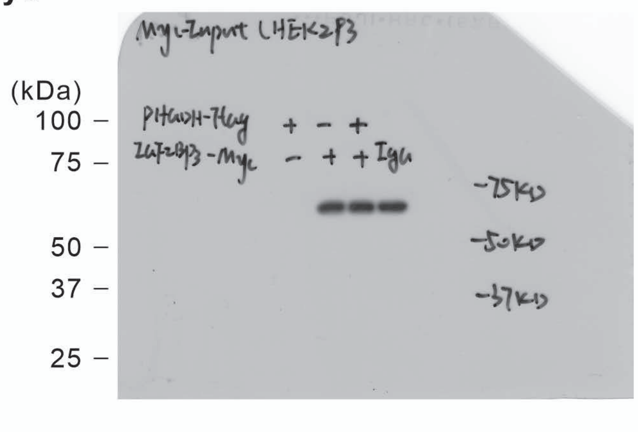

Full unedited gels for Fig.3e

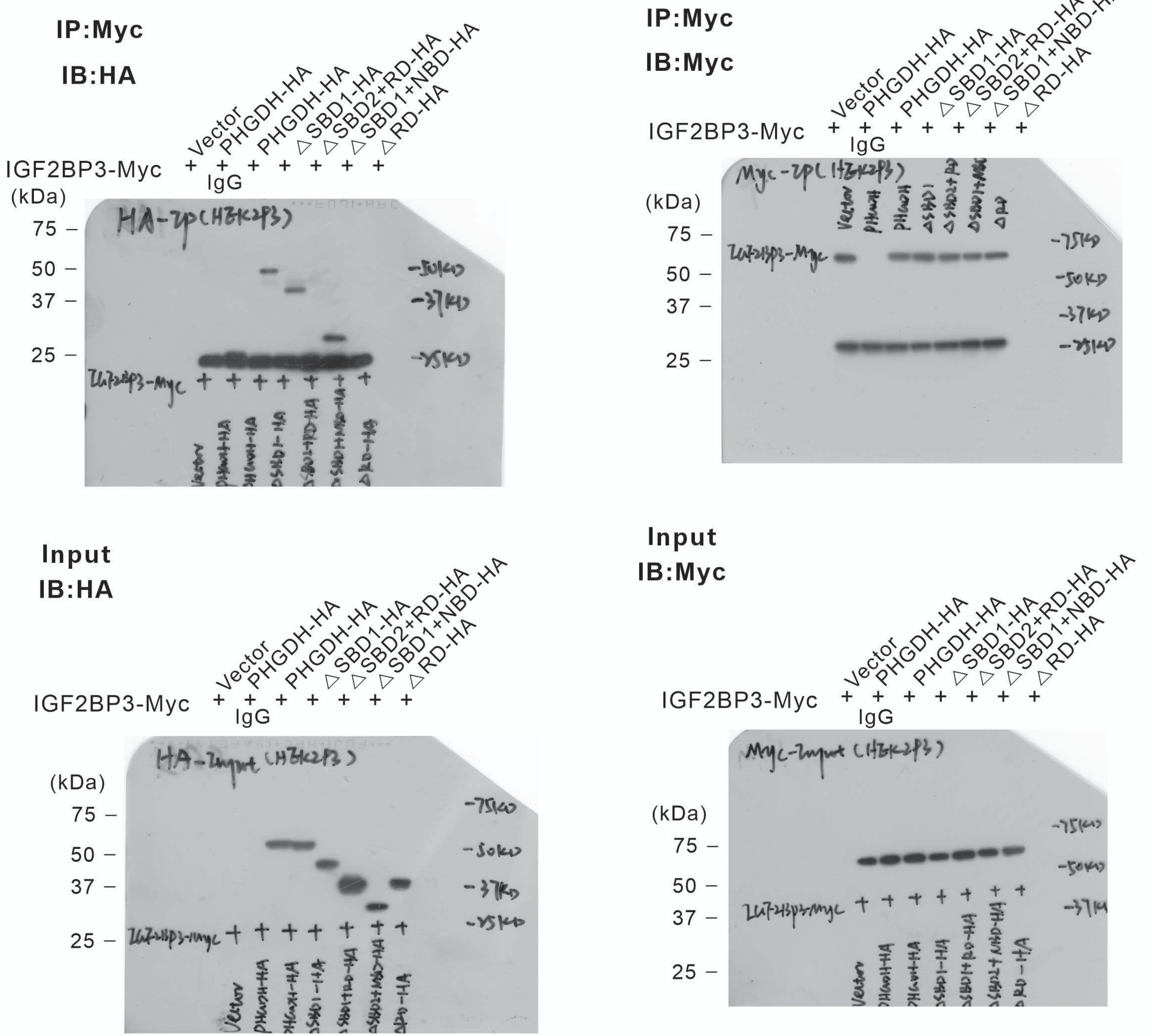

# Full unedited gels for Fig.3h

## PHGDH

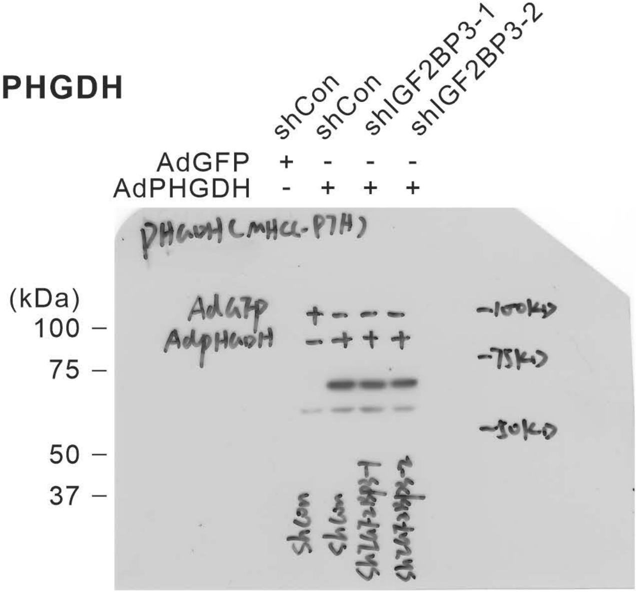


## IGF2BP3

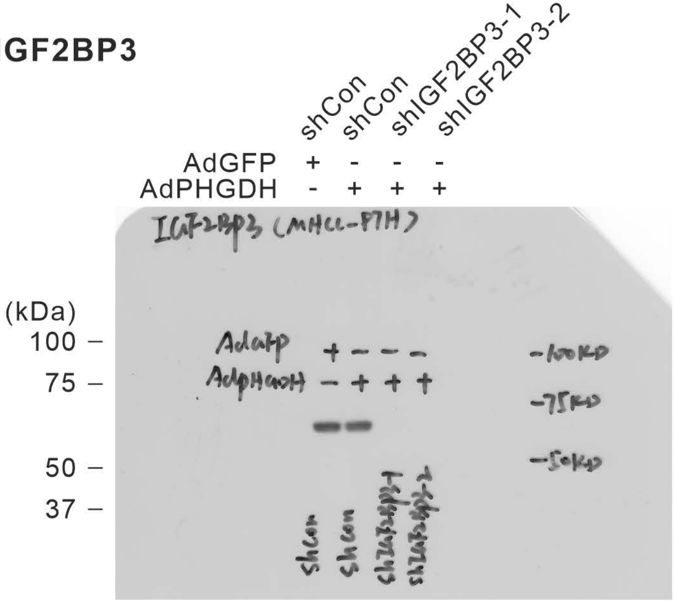


## PRKCD

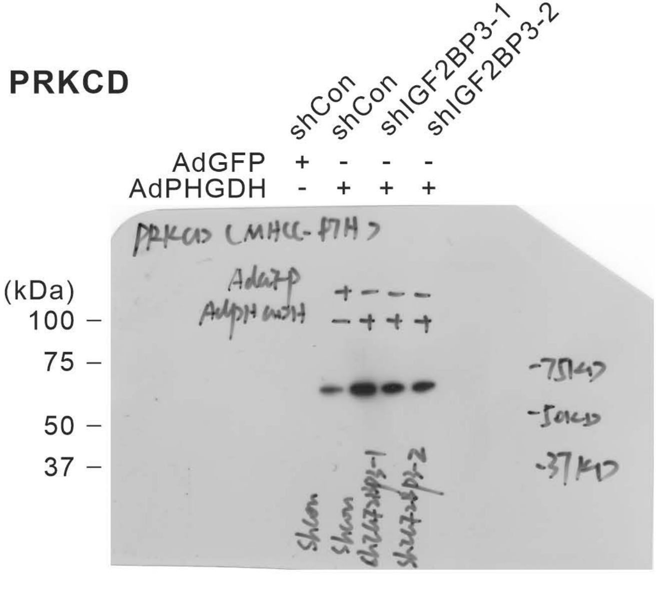


## β-actin

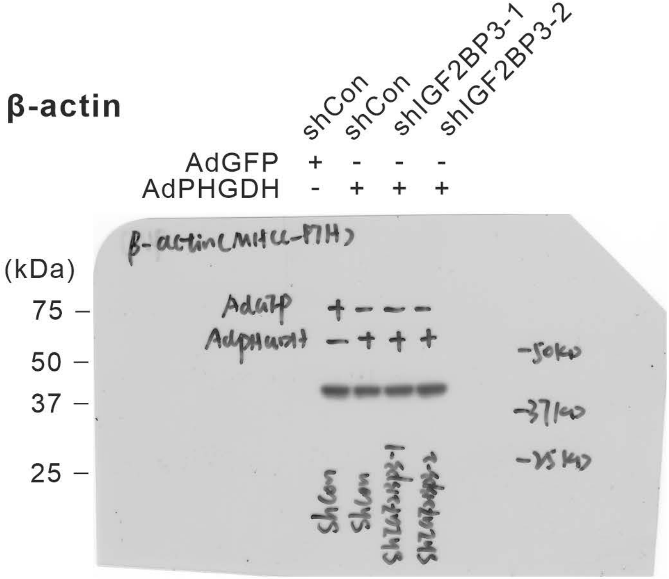

## Full unedited gels for Fig.4d-Left

### p-ULK1(S555)

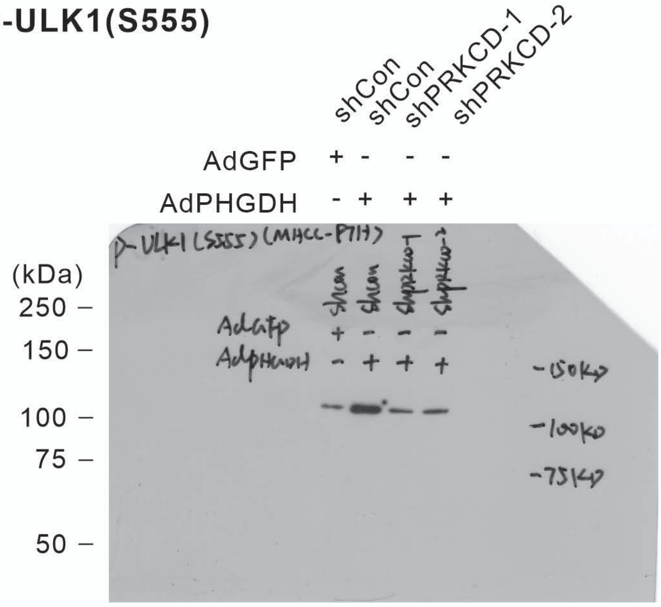


### p-Beclin1(S15)

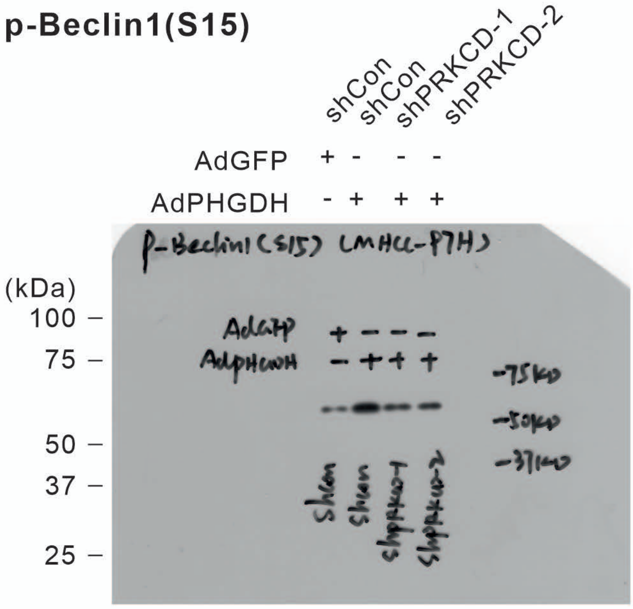


### PRKCD

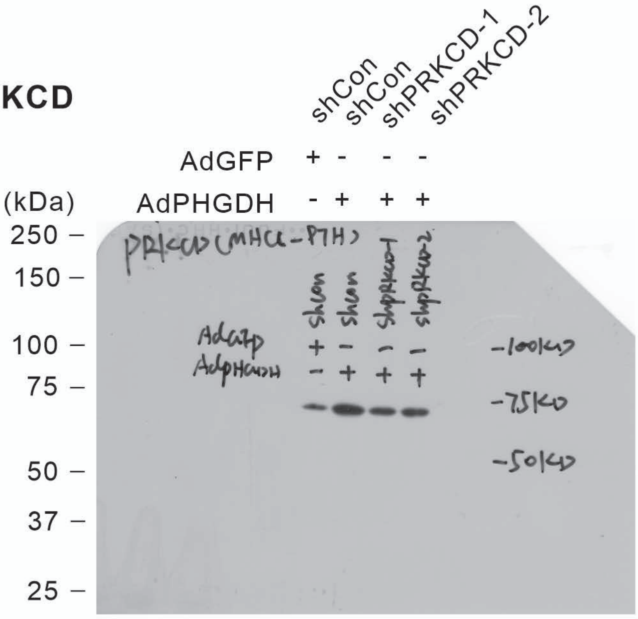


### PHGDH

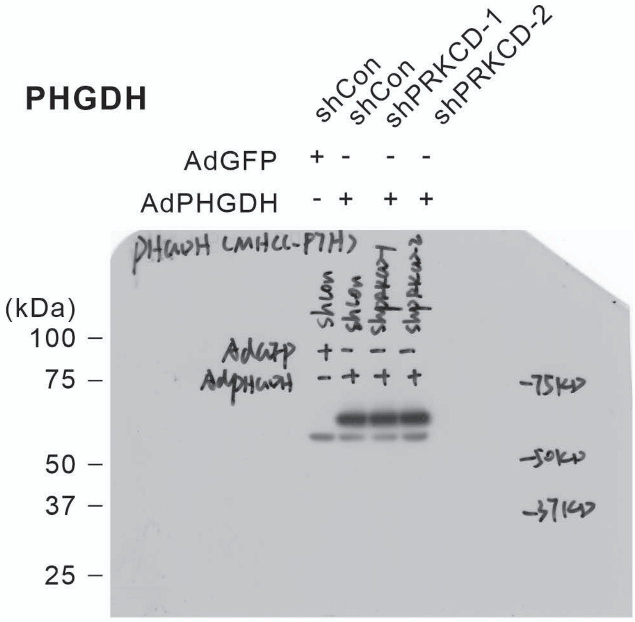


### BCL2

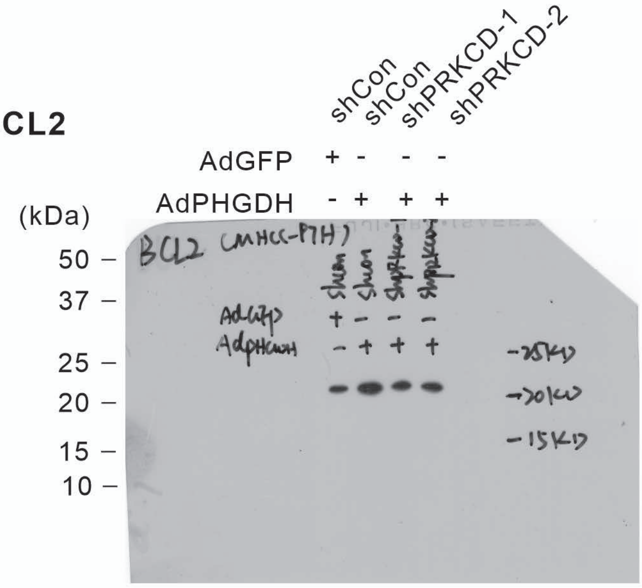


### BAX

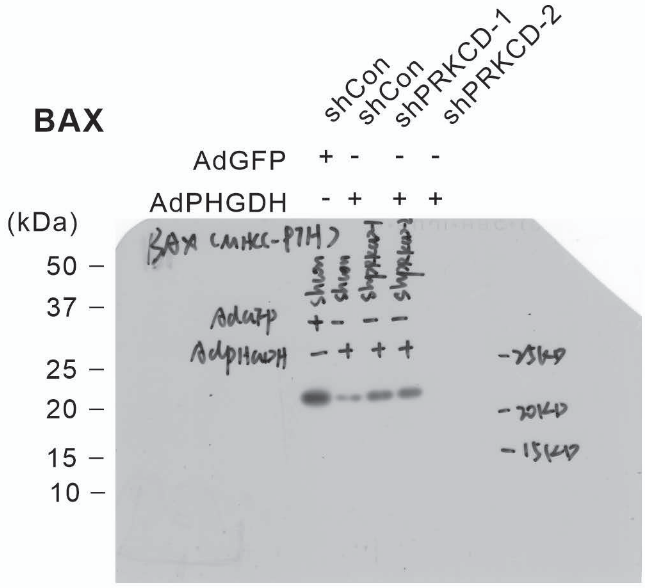


### β-actin

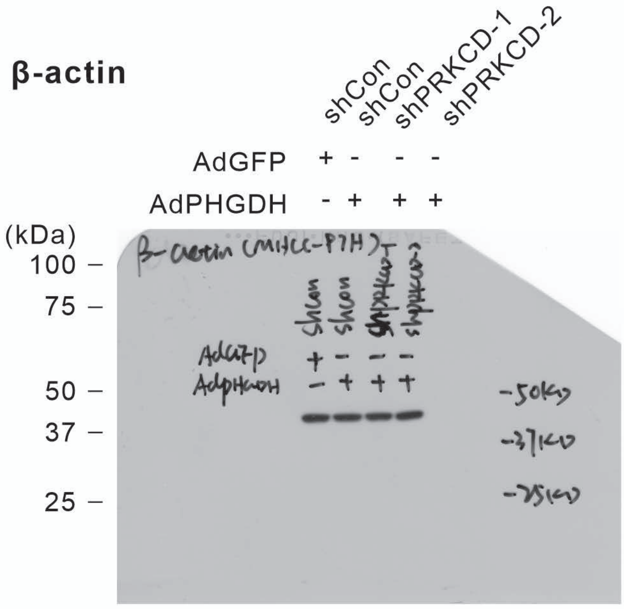

## Full unedited gels for Fig.4d-Right

### p-ULK1(S555)

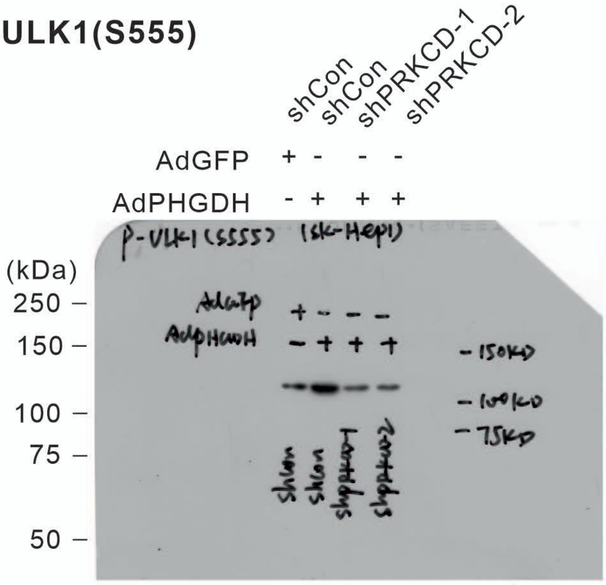


### p-Beclin1(S15)

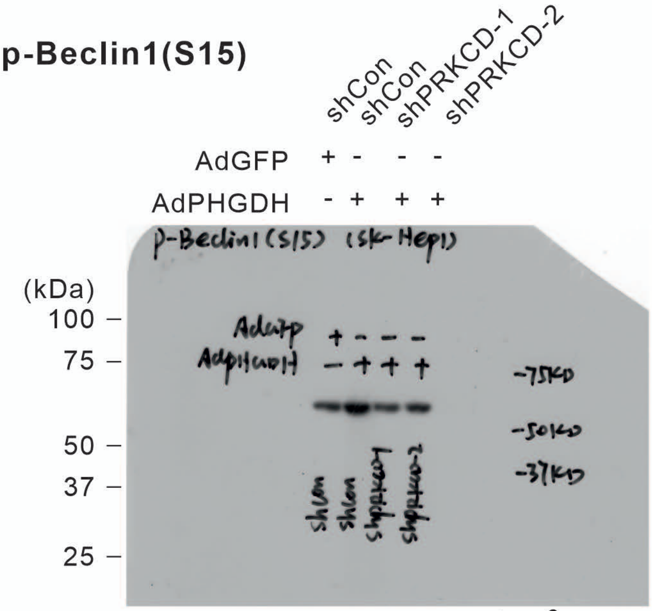


### PRKCD

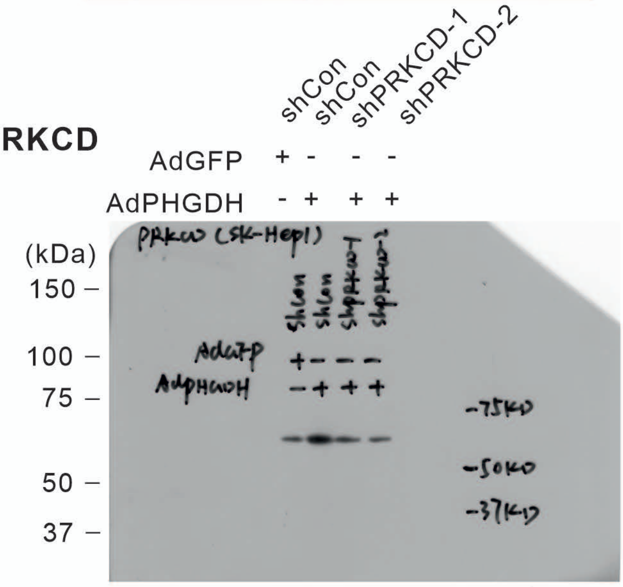


### PHGDH

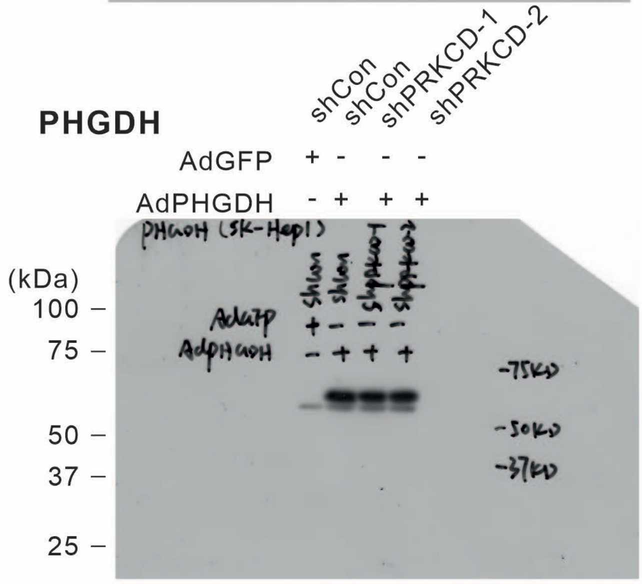


### BCL2

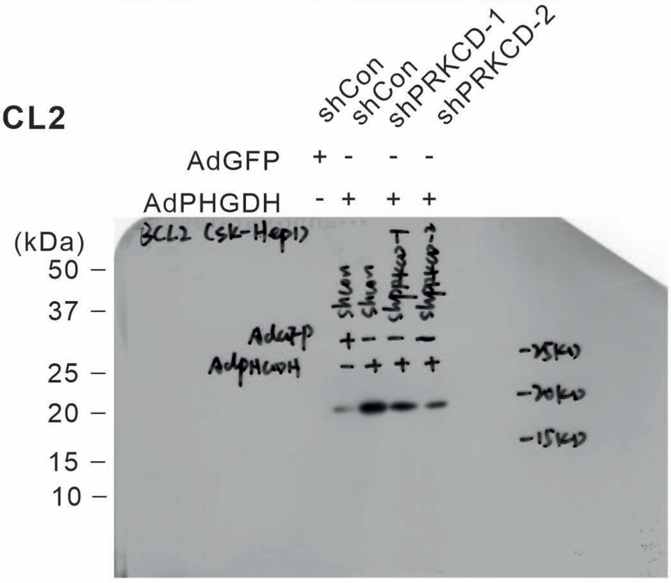


### BAX

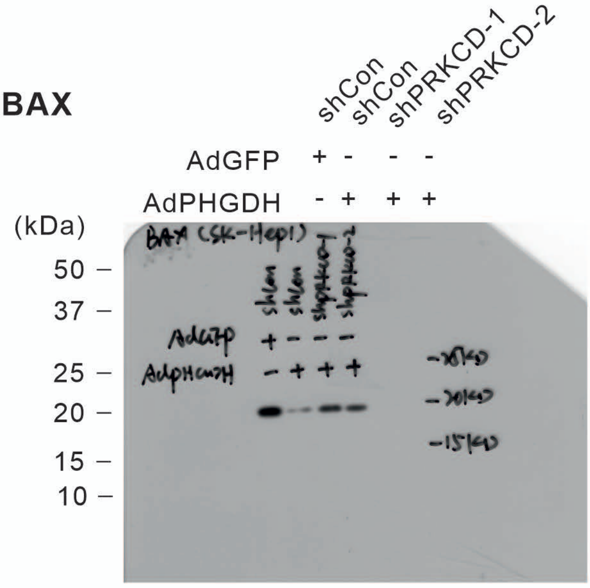


### β-actin

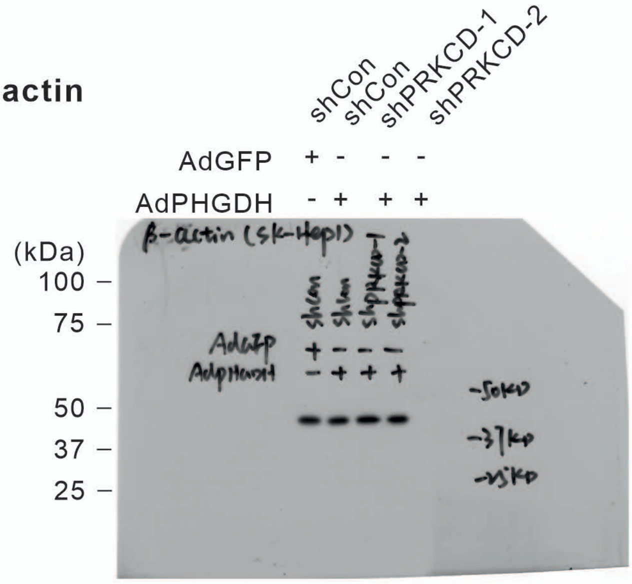

## Full unedited gels for Fig.4e-Left

### p-ULK1(S555)

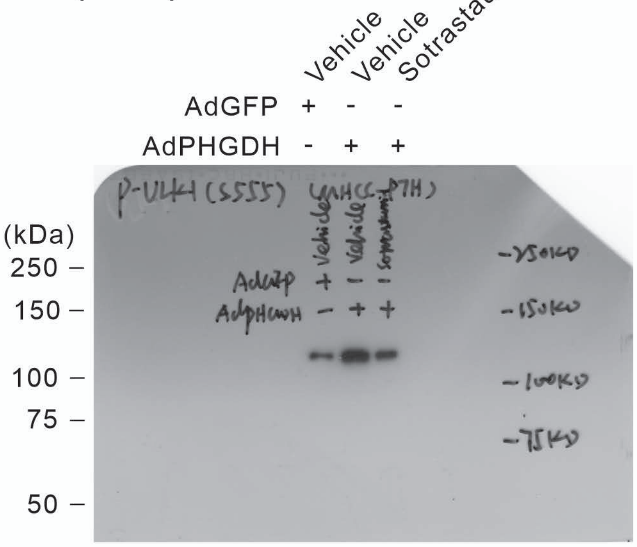


### p-Beclin1(S15)

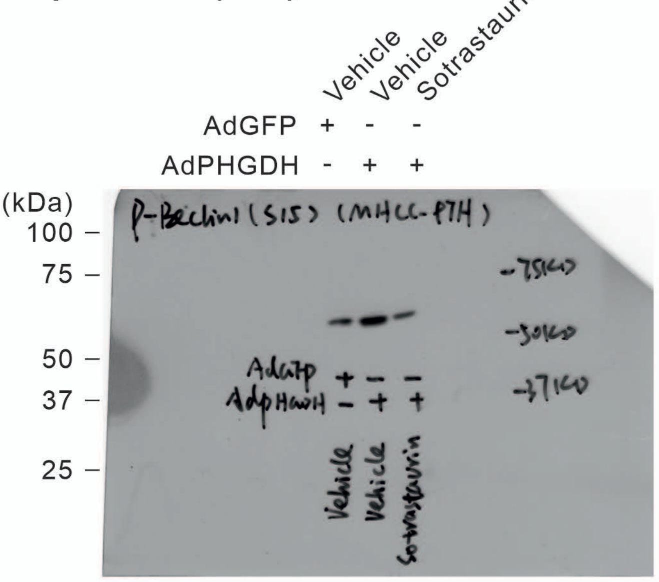


### PRKCD

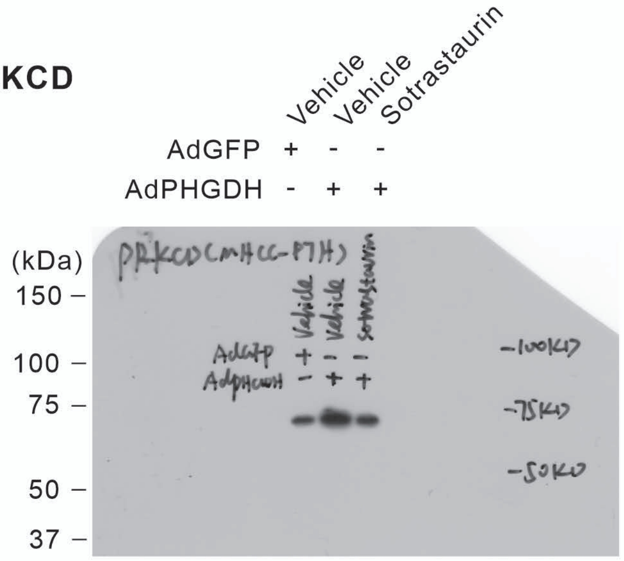


### PHGDH

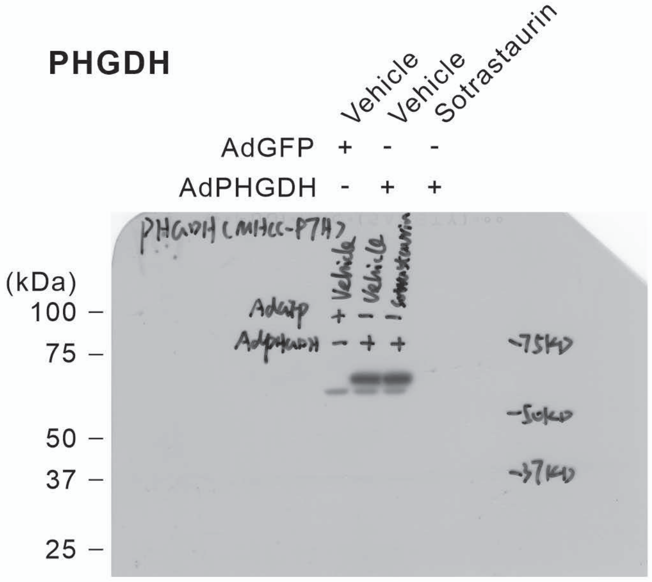


### BCL2

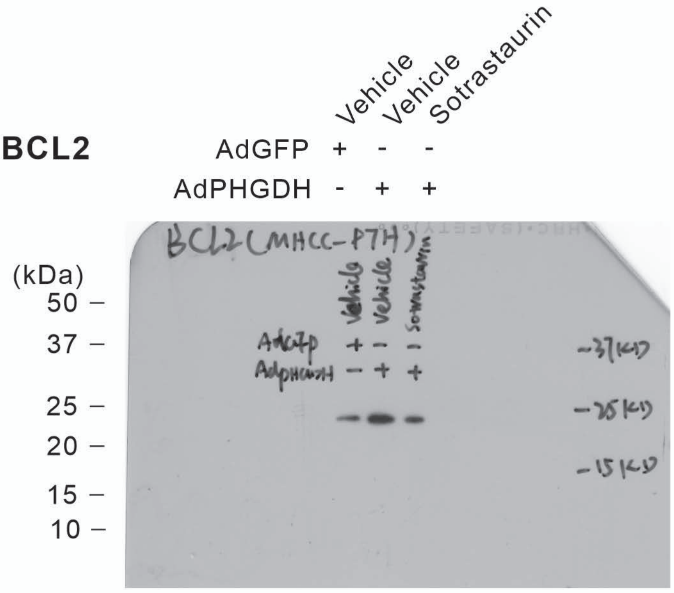


### BAX

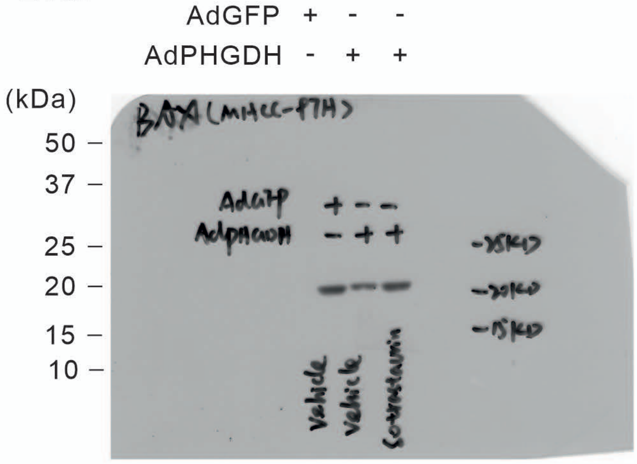


### β-actin

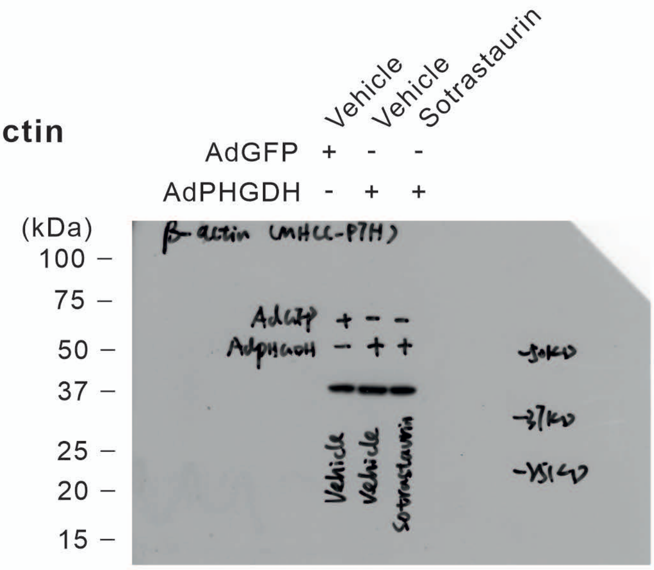

**Full unedited gels for Fig.4e-Right**

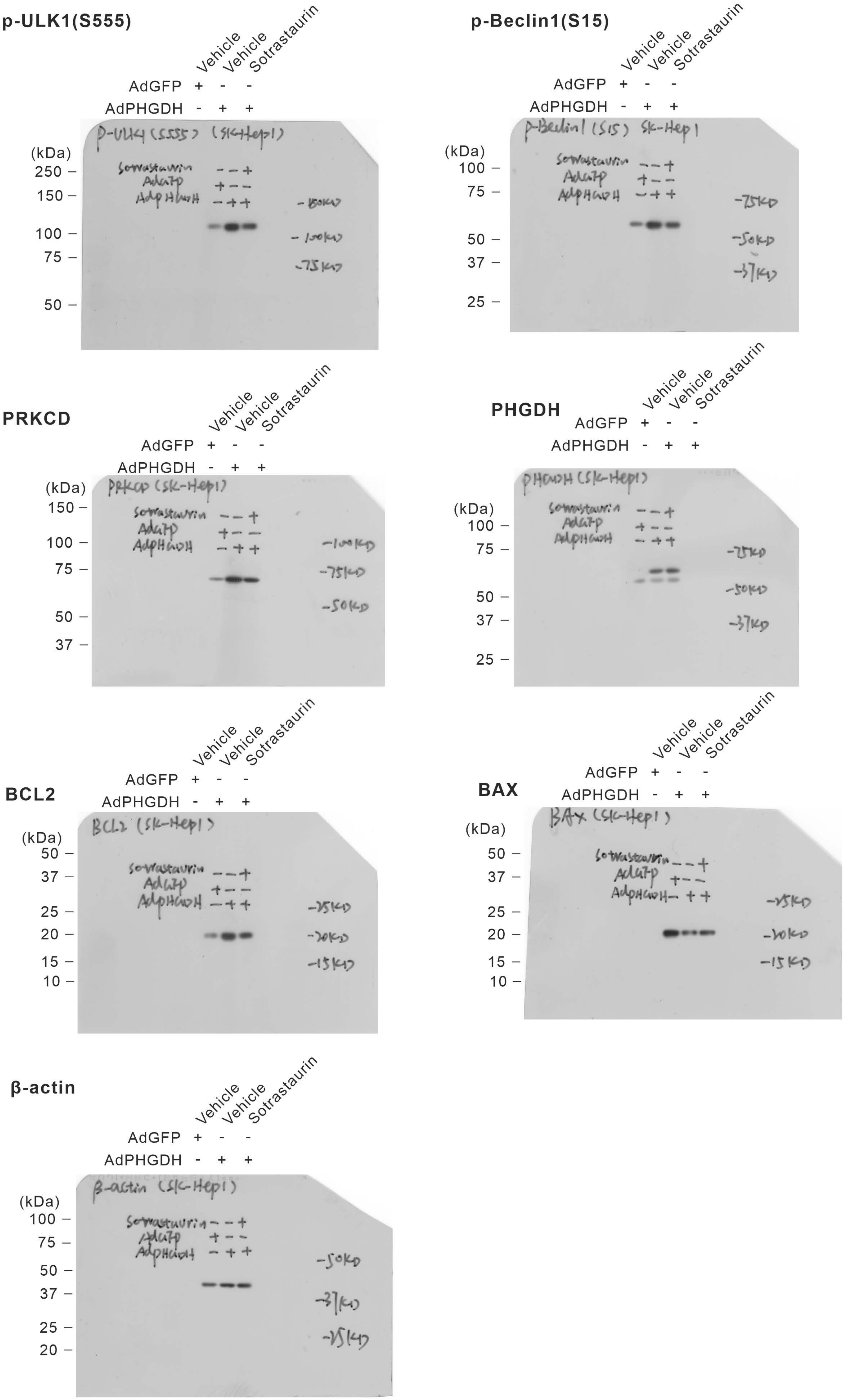

**Full unedited gels for Fig.5e**

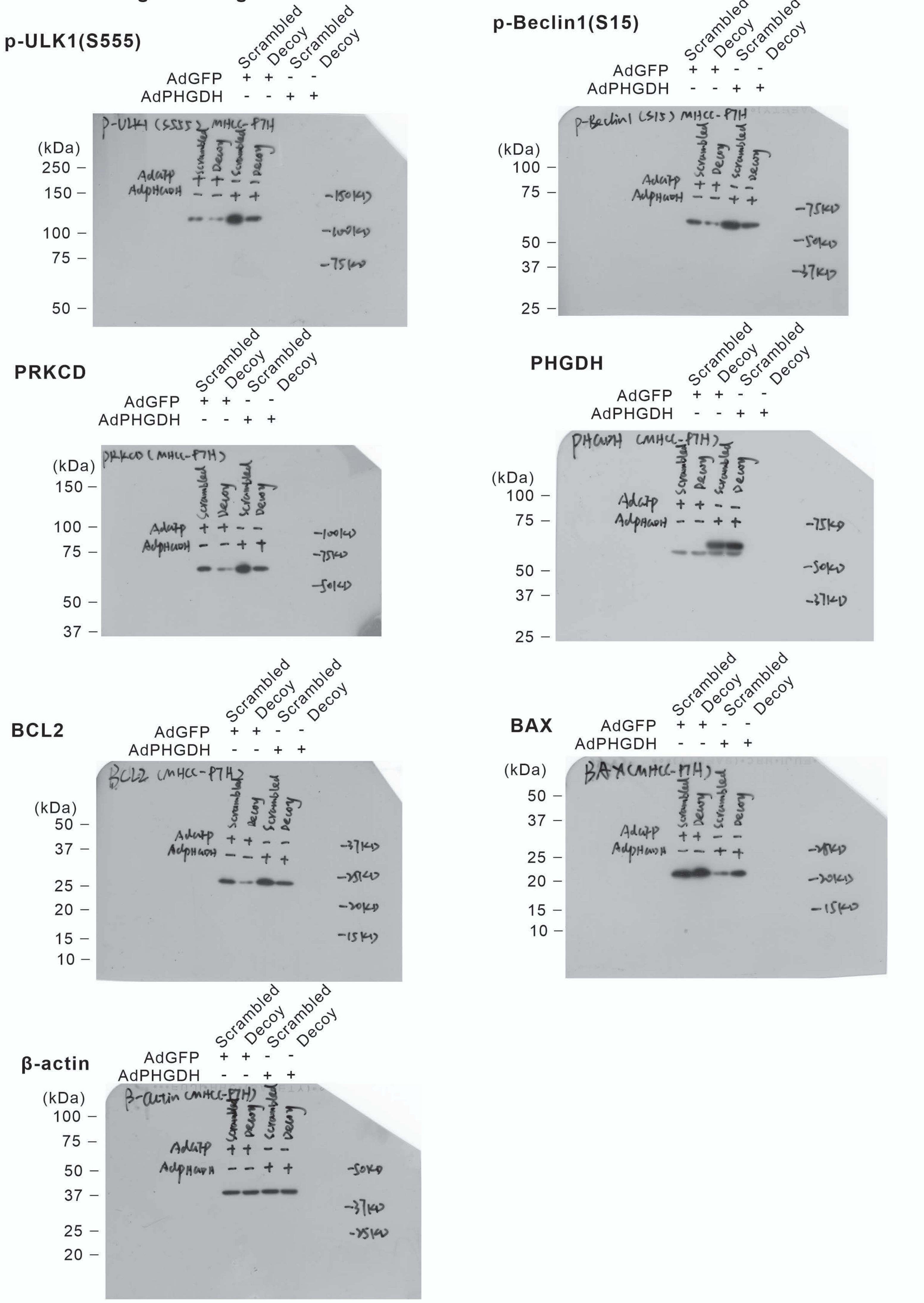

## Full unedited gels for Fig.6e

### p-ULK1(S555)

AAV-shCon: 1 2 3 4 5 6 | AAV-sh*Phgdh*: 1 2 3 4 5 6

(kDa) 250 – 150 – 100 – 75 – 50 –

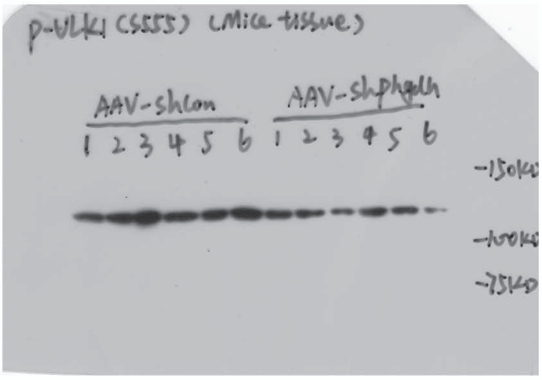

### p-Beclin1(S15)

AAV-shCon: 1 2 3 4 5 6 | AAV-sh*Phgdh*: 1 2 3 4 5 6

(kDa) 100 – 75 – 50 – 37 –

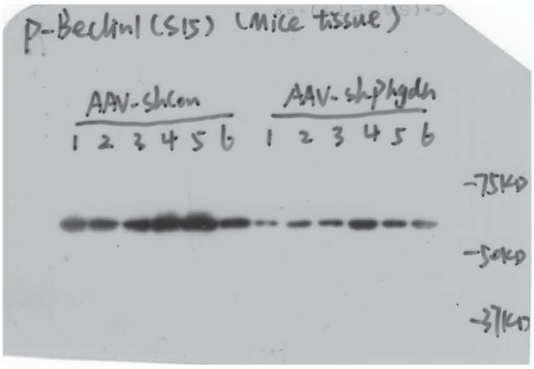

### PRKCD

AAV-shCon: 1 2 3 4 5 6 | AAV-sh*Phgdh*: 1 2 3 4 5 6

(kDa) 100 – 75 – 50 – 37 – 25 –

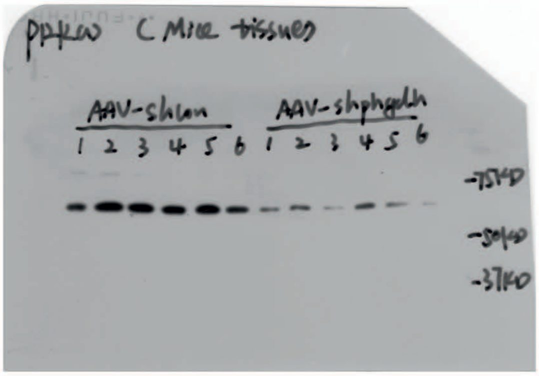

### PHGDH

AAV-shCon: 1 2 3 4 5 6 | AAV-sh*Phgdh*: 1 2 3 4 5 6

(kDa) 100 – 75 – 50 – 37 – 25 –

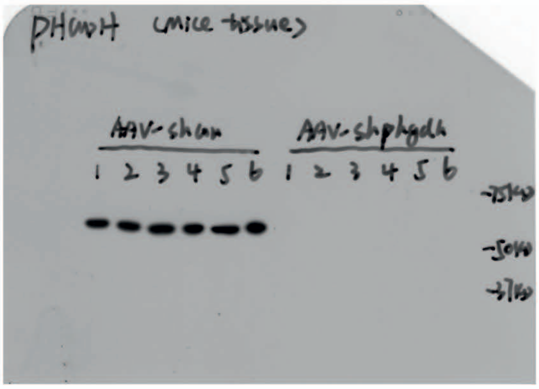

### BCL2

AAV-shCon: 1 2 3 4 5 6 | AAV-sh*Phgdh*: 1 2 3 4 5 6

(kDa) 50 – 37 – 25 – 20 – 15 – 10 –

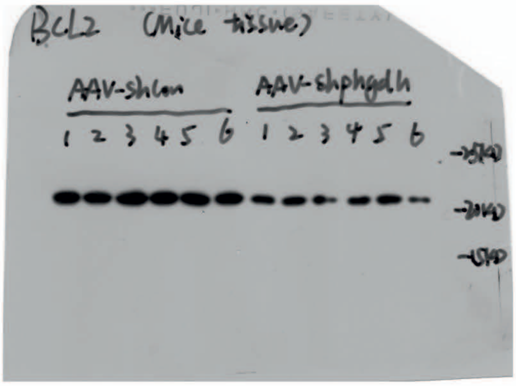

### BAX

AAV-shCon: 1 2 3 4 5 6 | AAV-sh*Phgdh*: 1 2 3 4 5 6

(kDa) 50 – 37 – 25 – 20 – 15 – 10 –

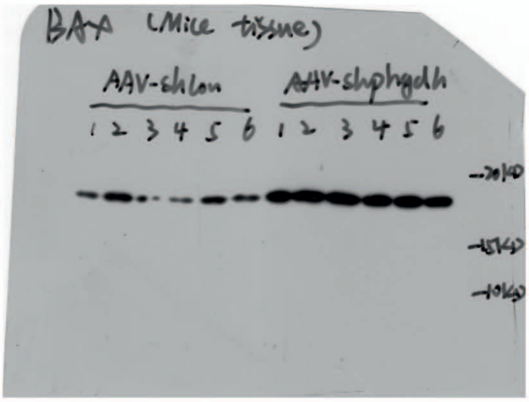

### β-actin

AAV-shCon: 1 2 3 4 5 6 | AAV-sh*Phgdh*: 1 2 3 4 5 6

(kDa) 100 – 75 – 50 – 37 – 25 –

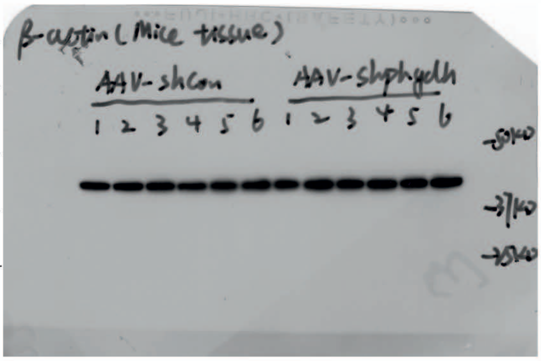

**Full unedited gels for Fig.6I**

**p-ULK1(S555)**

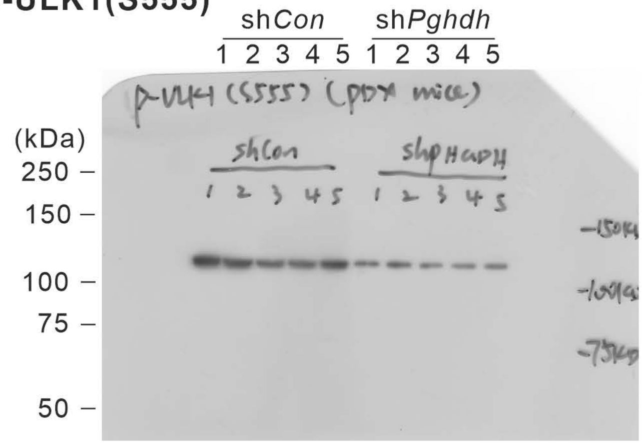


**p-Beclin1(S15)**

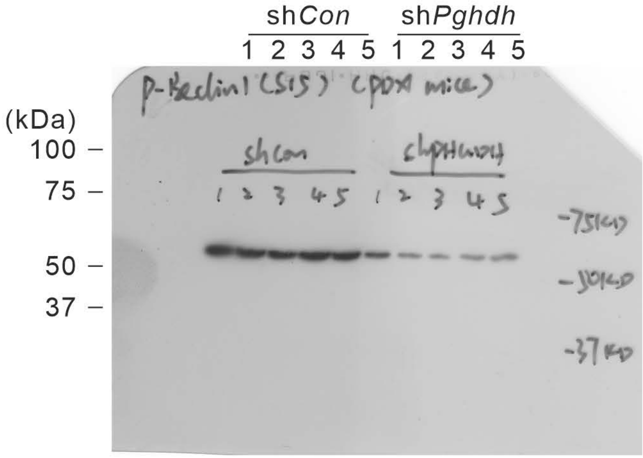


**PRKCD**

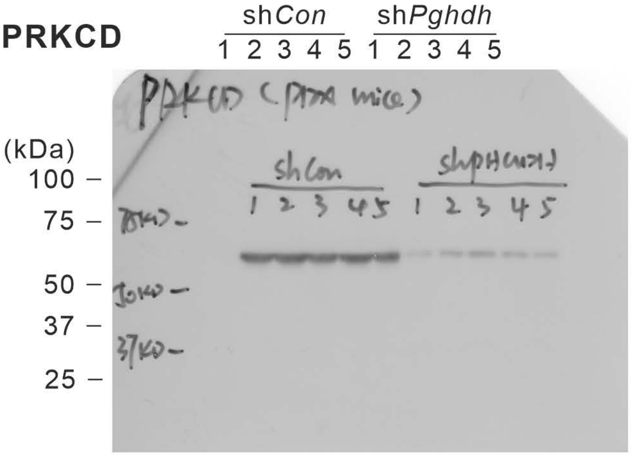


**PHGDH**

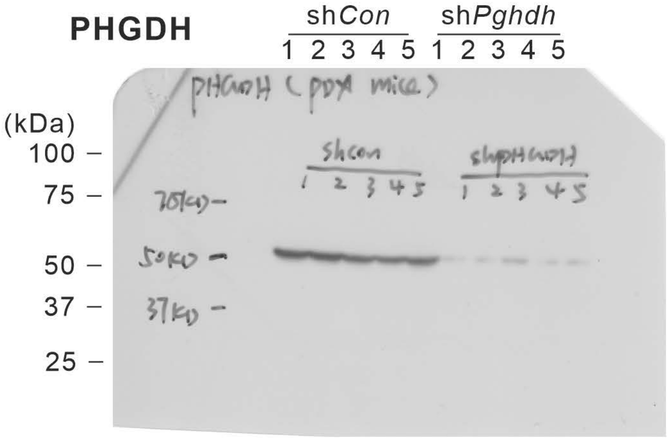


**β-actin**

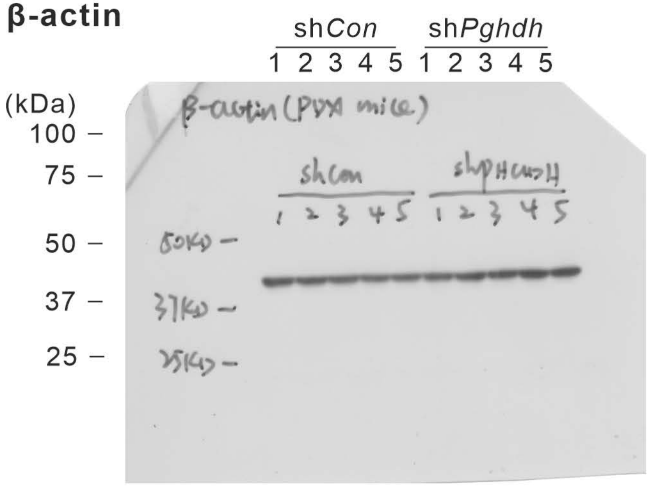

# Full unedited gels for Extended Data Fig.2l

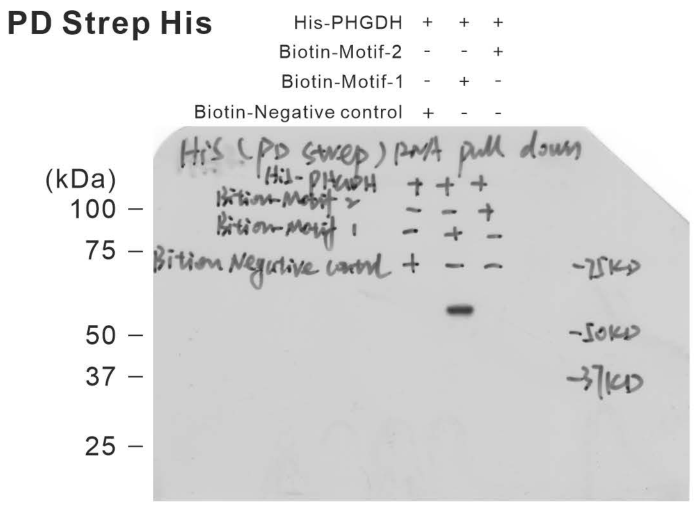


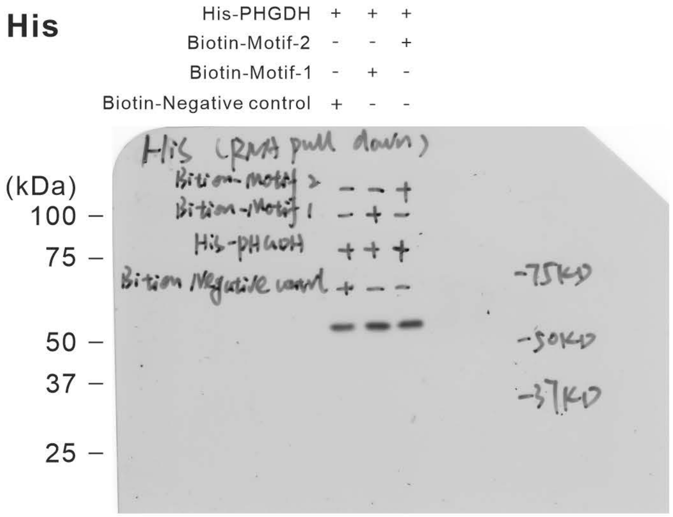

## Full unedited gels for Extended Data Fig.2p

### PRKCD

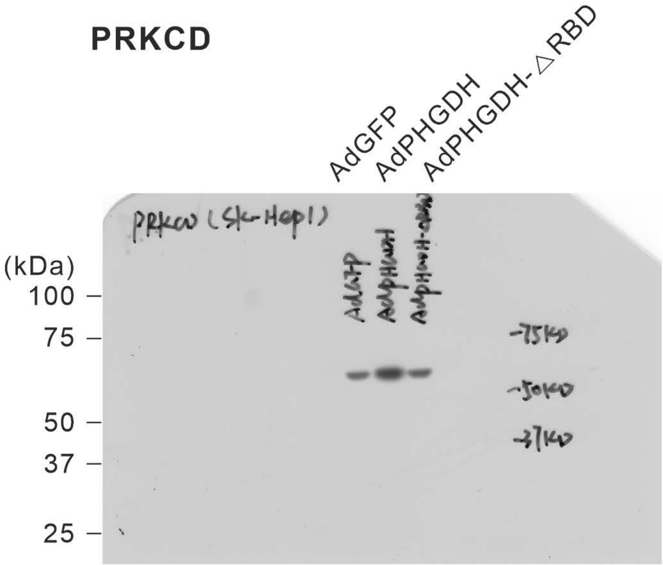


### PHGDH

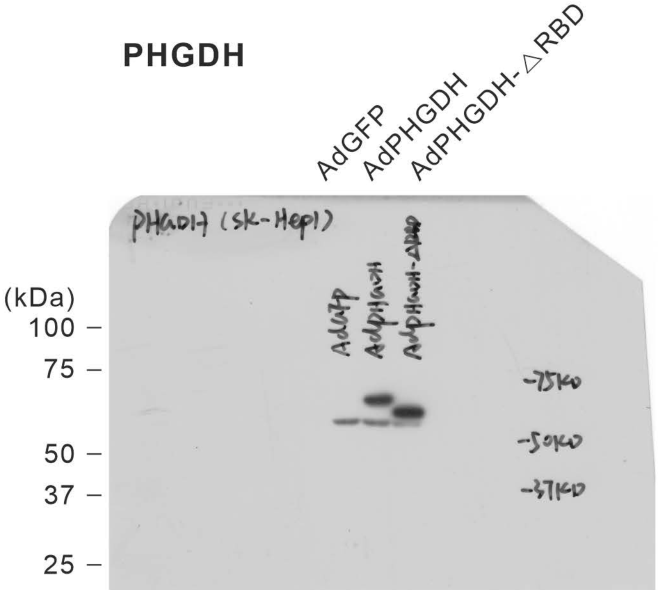


### β-actin

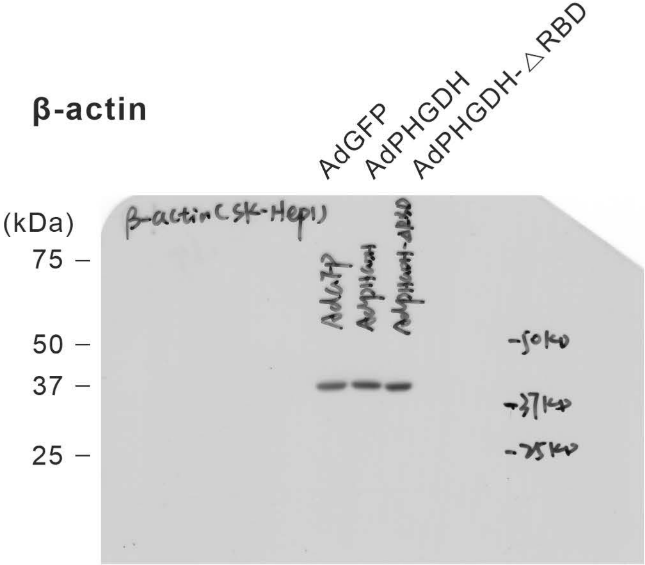

**Full unedited gels for Extended Data Fig.2q**

**PRKCD**

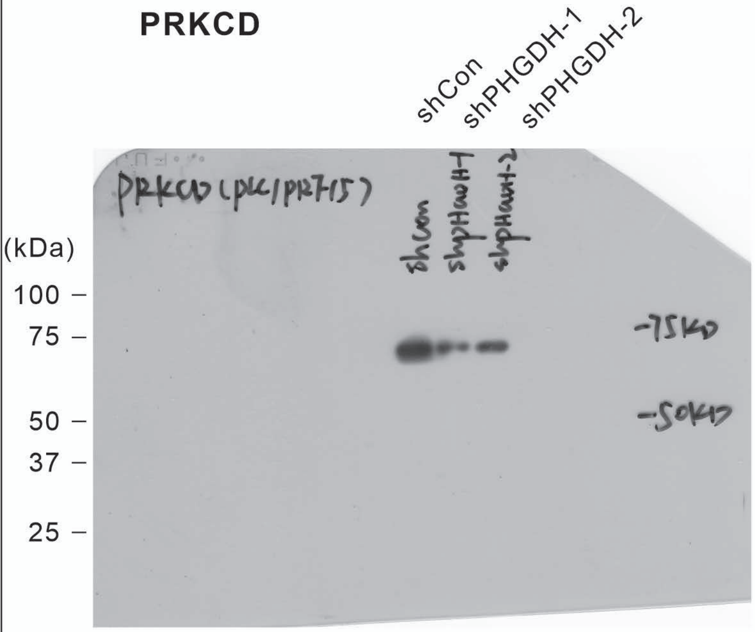


**PHGDH**

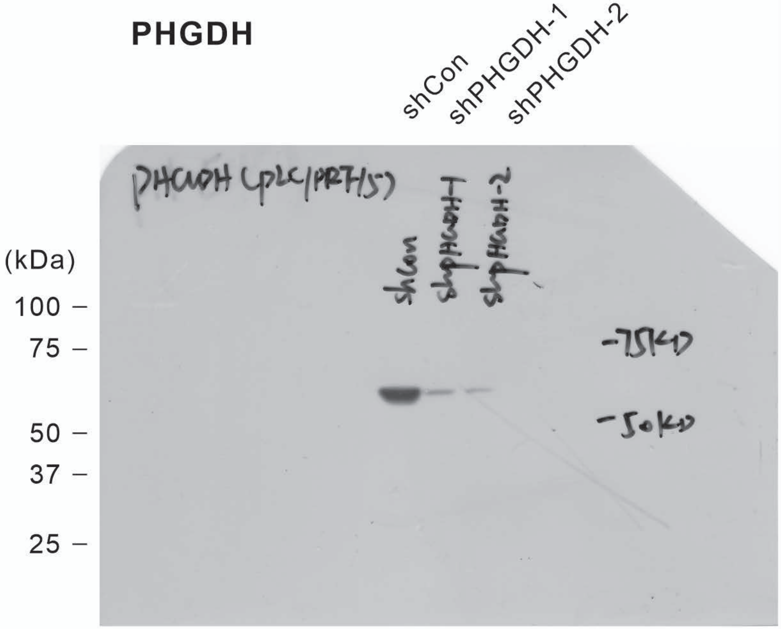


**β-actin**

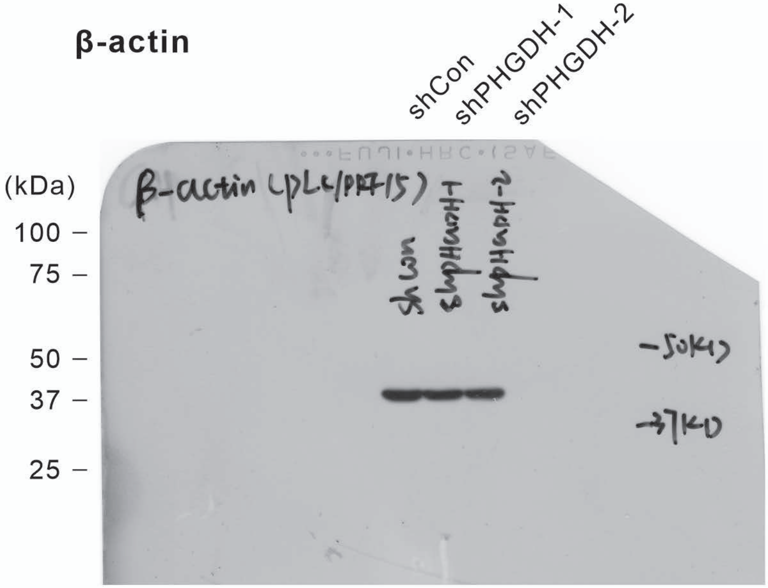

**Full unedited gels for Extended Data Fig.2r**

**PRKCD**

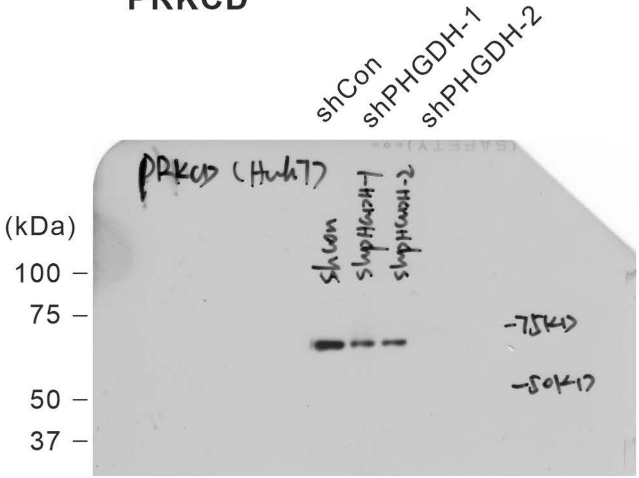


**PHGDH**

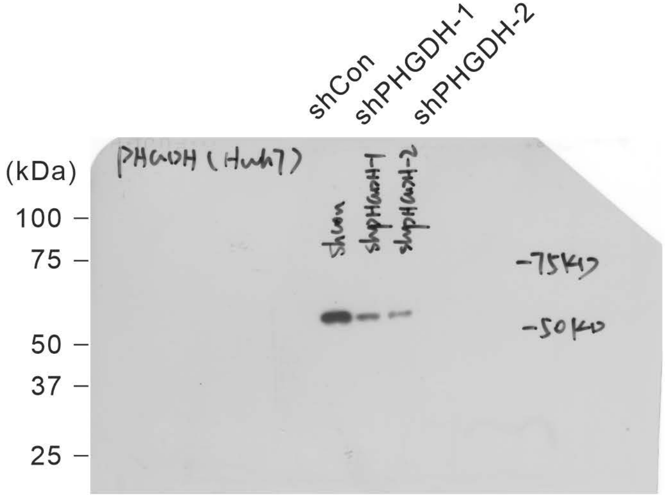


**β-actin**

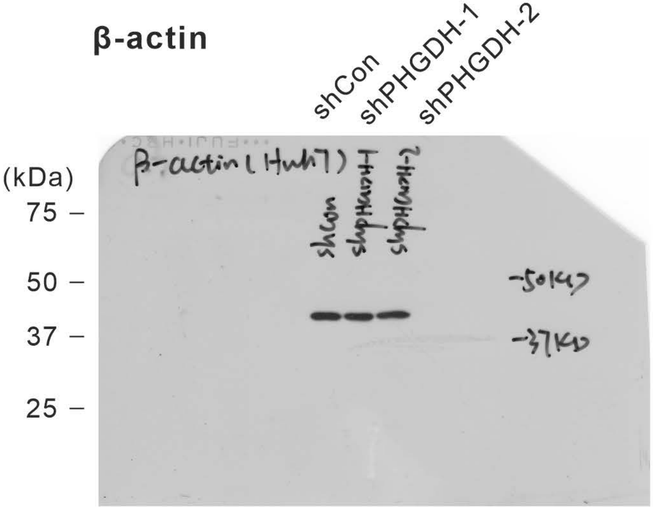

**Full unedited gels for Extended Data Fig.3a**

**IP:Flag**
**IB:Flag**

PHGDH-Flag + - +
IGF2BP3-Myc - + + IgG

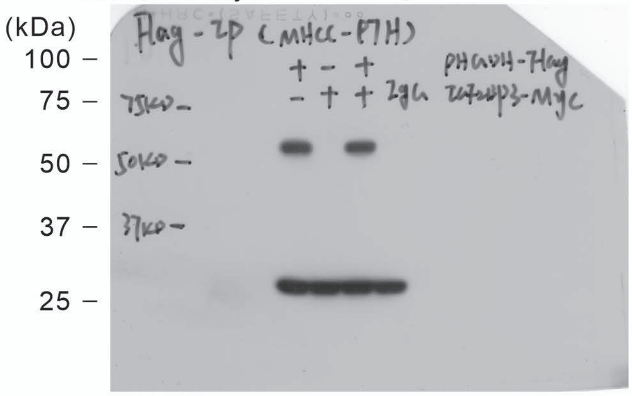


**IP:Flag**
**IB:Myc**

PHGDH-Flag + - +
IGF2BP3-Myc - + + IgG

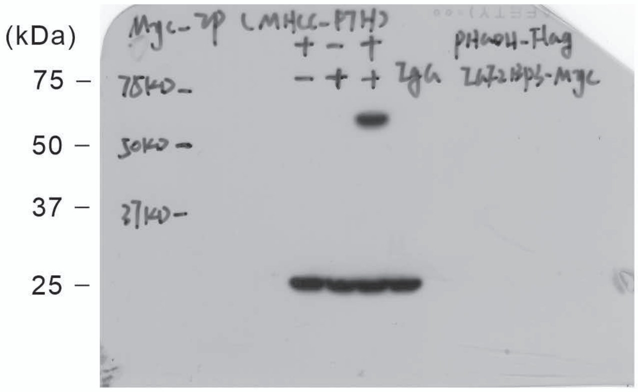


**Input**
**IB:Flag**

PHGDH-Flag + - +
IGF2BP3-Myc - + + IgG

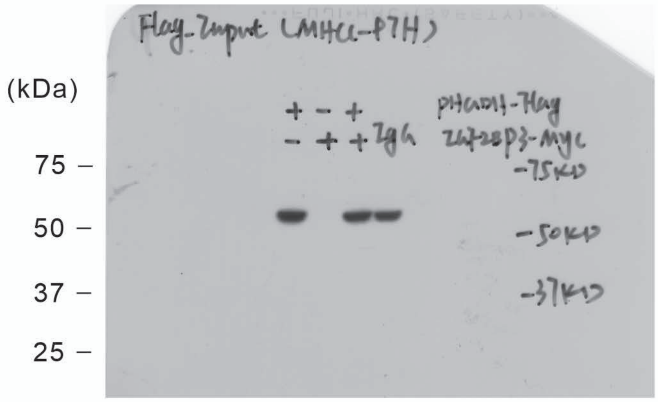


**Input**
**IB:Myc**

PHGDH-Flag + - +
IGF2BP3-Myc - + + IgG

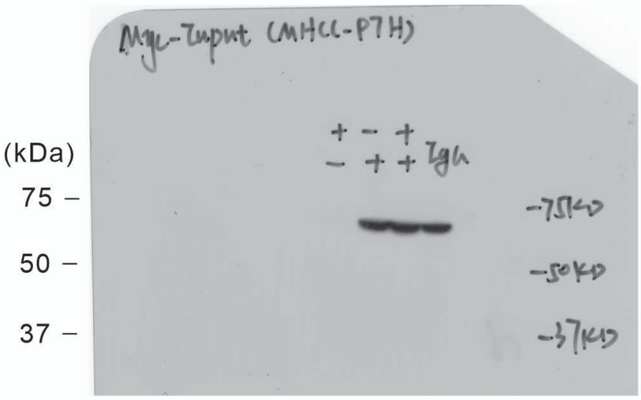


**IP:Myc**
**IB:Flag**

PHGDH-Flag + - +
IGF2BP3-Myc - + + IgG

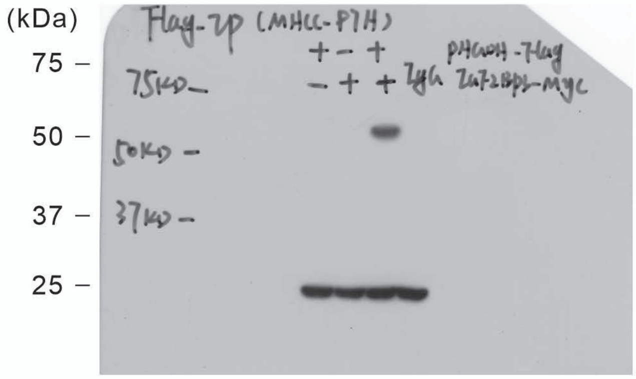


**IP:Myc**
**IB:Myc**

PHGDH-Flag + - +
IGF2BP3-Myc - + + IgG

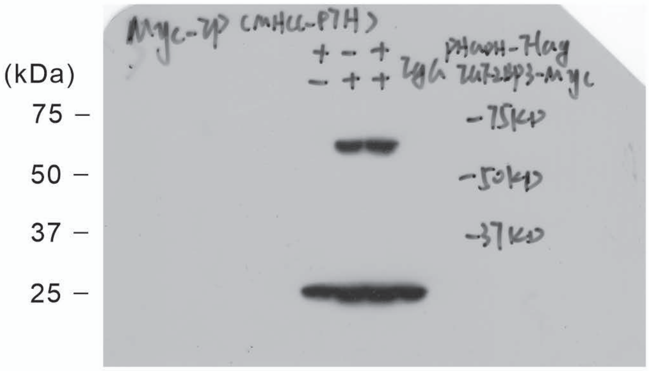


**Input**
**IB:Flag**

PHGDH-Flag + - +
IGF2BP3-Myc - + + IgG

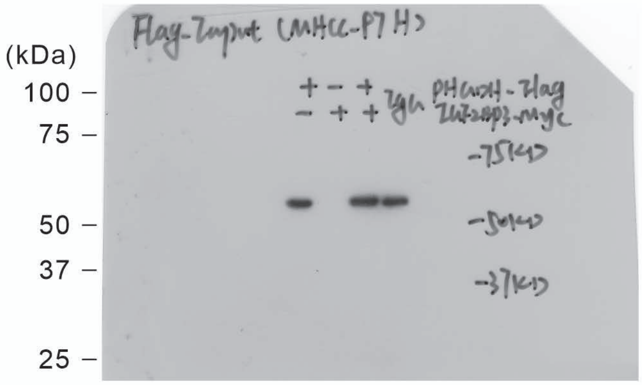


**Input**
**IB:Myc**

PHGDH-Flag + - +
IGF2BP3-Myc - + + IgG

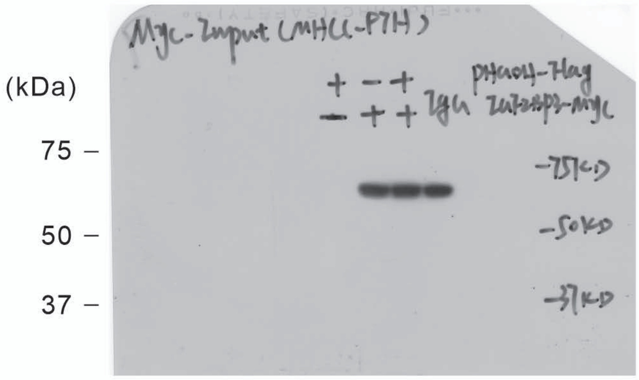

Full unedited gels for Extended Data Fig.3b

**Input**
**IB:HNRNPA1**

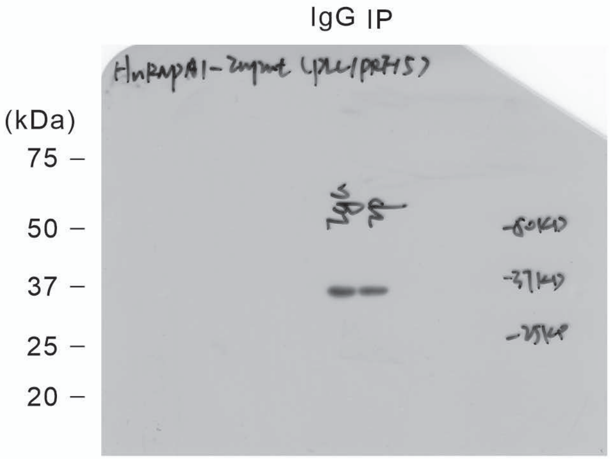


**Input**
**IB:PHGDH**

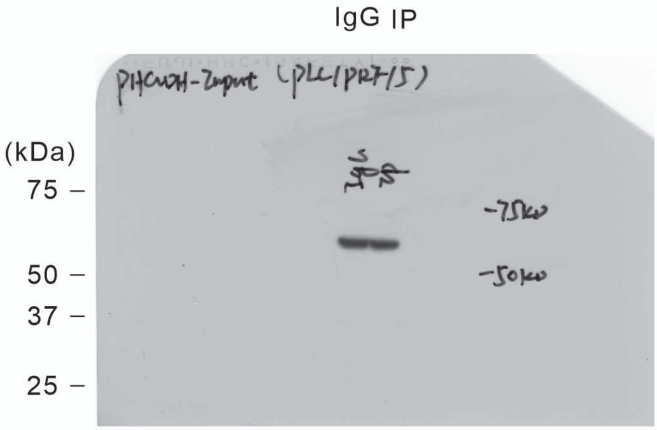


**IP:PHGDH**
**IB:HNRNPA1**

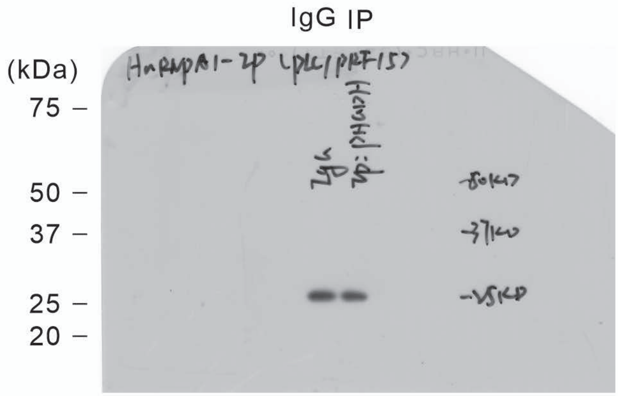


**IP:PHGDH**
**IB:PHGDH**

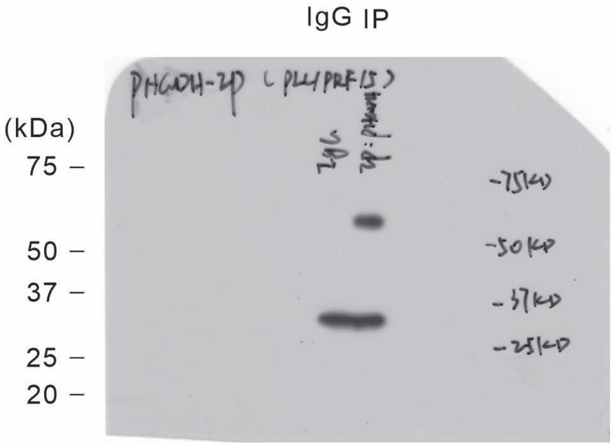


**IP:HNRNPA1**
**IB:HNRNPA1**

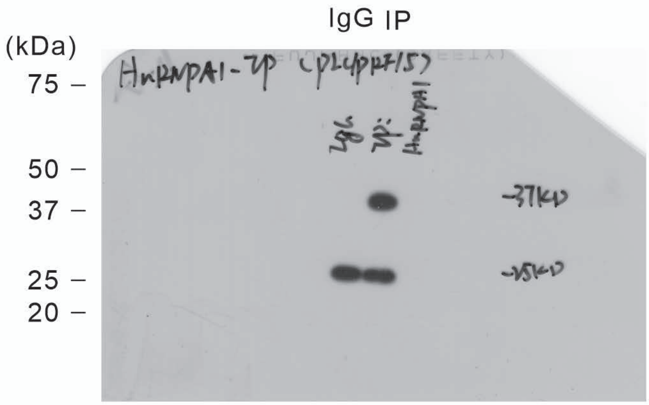


**IP:HNRNPA1**
**IB:PHGDH**

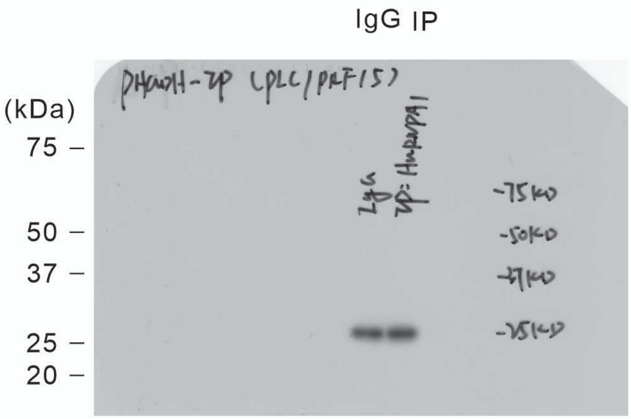

Full unedited gels for Extended Data Fig.3c

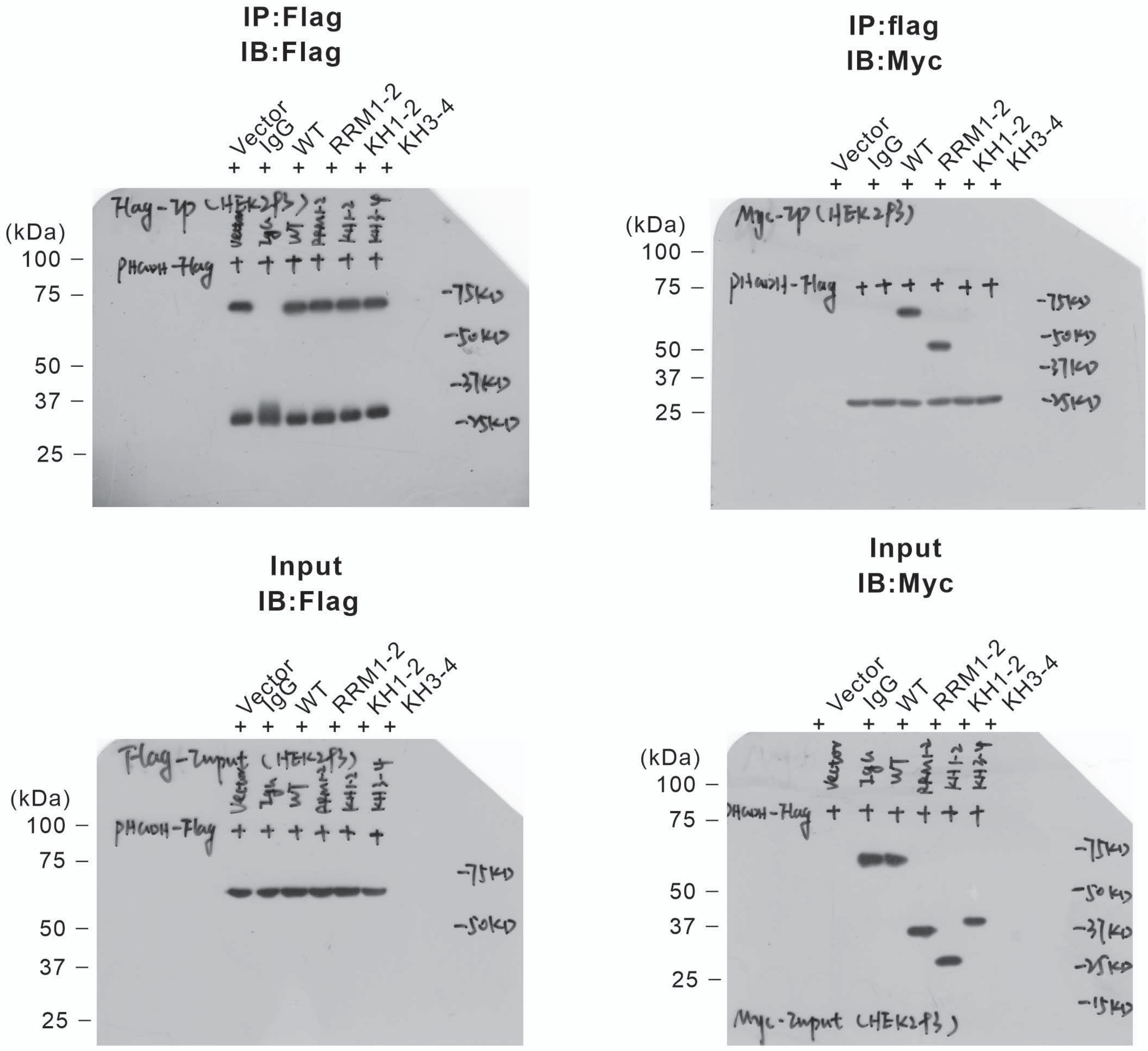

**Full unedited gels for Extended Data Fig.5c**

**p-ULK1(S555)**

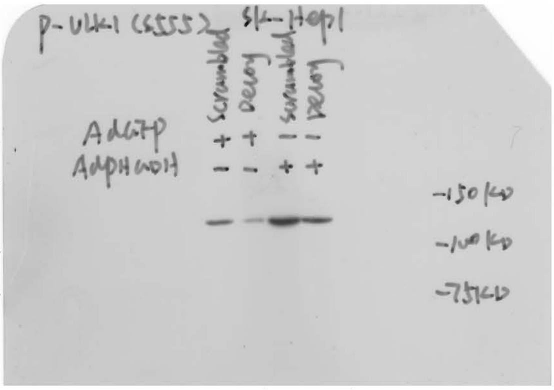


**p-Beclin1(S15)**

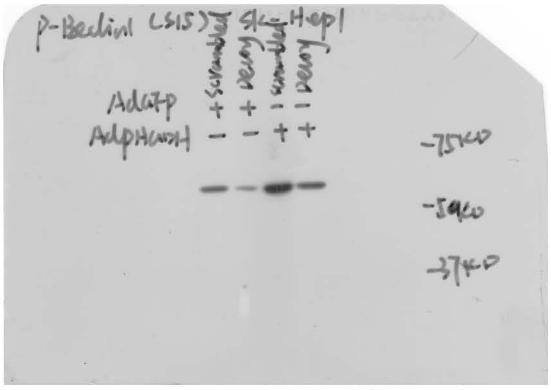


**PRKCD**

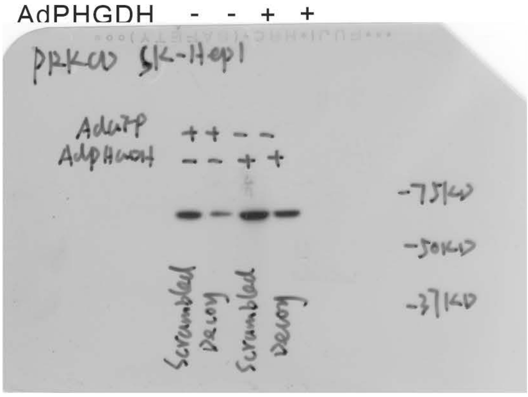


**PHGDH**

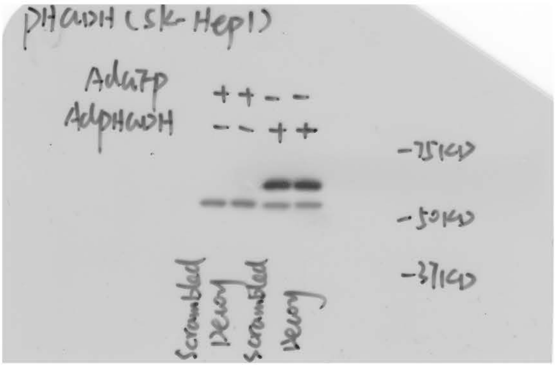


**BCL2**

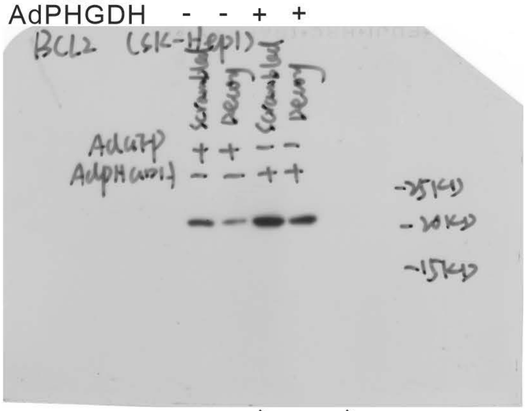


**β-actin**

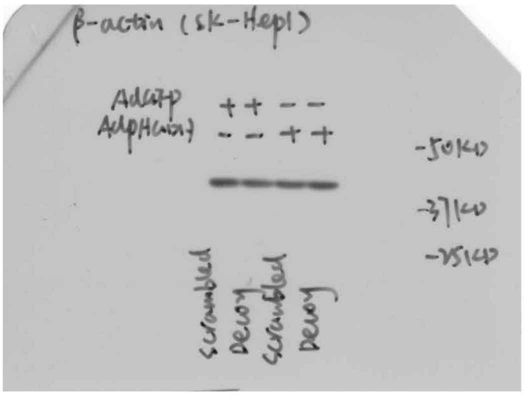


**BAX**

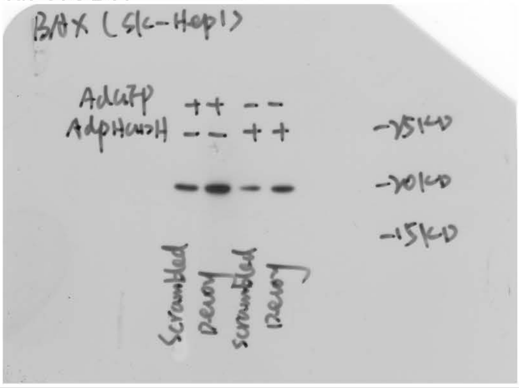

Supplement: Supplementary file 3 — Unprocessed Western blots [file 41392_2025_2304_MOESM3_ESM.pdf]
